# Three decades of population health changes in Japan, 1990–2021: a subnational analysis for the Global Burden of Disease Study 2021

**DOI:** 10.1016/S2468-2667(25)00044-1

**Published:** 2025-03-20

**Authors:** GBD 2021 Japan Collaborators, Shuhei Nomura, Michio Murakami, Santosh Kumar Rauniyar, Naoki Kondo, Takahiro Tabuchi, Haruka Sakamoto, Yasuharu Tokuda, Nishali Patel, Jose Navarro de Pablo, Joseph L Dieleman, Angela Y Chang, Vegard Skirbekk, Sarah K Abe, Norito Kawakami, Erika Ota, Scott D Glenn, Chimedsuren Ochir, Hiroaki Miyata, Manami Inoue, Kenji Shibuya, Isaac Yeboah Addo, Mohammed Ahmed Akkaif, Syed Mahfuz Al Hasan, Waad Ali, Mohammad Al-Wardat, Hany Aly, Anayochukwu Edward Anyasodor, Jalal Arabloo, Ahmed Y. Azzam, Kavita Batra, Sonu Bhaskar, Samuel Adolf Bosoka, Ester Cerin, Vijay Kumar Chattu, Dong-Woo Choi, Bryan Chong, Samuel Demissie Darcho, Nicole Davis Weaver, Kuldeep Dhama, Robert Kokou Dowou, Temitope Cyrus Ekundayo, Ibrahim Farahat El Bayoumy, Pietro Ferrara, Nuno Ferreira, Takeshi Fukumoto, Xiang Gao, Samer Hamidi, Simon I Hay, Yuta Hiraike, Mehdi Hosseinzadeh, Nayu Ikeda, Arit Inok, Md. Rabiul Islam, Masao Iwagami, Ammar Abdulrahman Jairoun, Mihajlo Jakovljevic, Inn Kynn Khaing, Mohammad Jobair Khan, Atulya Aman Khosla, Tea Lallukka, Thao Thi Thu Le, Munjae Lee, Seung Won Lee, Wei-Chen Lee, Raimundas Lunevicius, Medha Mathur, Hadush Negash Meles, Mohammadreza Mobayen, Jama Mohamed, Abdollah Mohammadian-Hafshejani, Yanjinlkham Munkhsaikhan, Christopher J L Murray, Ganesh R Naik, Samidi Nirasha Kumari Navaratna, Phuong The Nguyen, Dieta Nurrika, Bogdan Oancea, Michael Safo Oduro, Takayoshi Ohkubo, Osaretin Christabel Okonji, Sok King Ong, Mahesh Padukudru P A, Jagadish Rao Padubidri, Romil R Parikh, Sungchul Park, Mahmoud Mohammed Ramadan, Shakthi Kumaran Ramasamy, Sheena Ramazanu, Elrashdy M. Moustafa Mohamed Redwan, Taeho Gregory Rhee, Cameron John Sabet, Vijaya Paul Samuel, Jennifer Saulam, Mohammad Ali Shamshirgaran, Premalatha K Shetty, Mika Shigematsu, Aminu Shittu, Emmanuel Edwar Siddig, Zhong Sun, Chandan Kumar Swain, Ruri Syailendrawati, Sree Sudha T Y, Jabeen Taiba, Masayuki Teramoto, Ngoc Ha Tran, Nguyen Tran Minh Duc, Dominique Vervoort, Muhammad Waqas, Kazumasa Yamagishi, Yuichiro Yano, Yuichi Yasufuku, Dong Keon Yon, Naohiro Yonemoto, Iman Zare, Zhiqiang Zhang, Hanqing Zhao, Claire Chenwen Zhong, Mohsen Naghavi

## Abstract

**Background:**

Given Japan's rapidly ageing demographic structure, comprehensive and long-term evaluations of its national and subnational health progress are important to inform public health policy. This study aims to assess Japan's population health, using the Global Burden of Diseases, Injuries, and Risk Factors Study (GBD) 2021 to analyse the country's evolving disease patterns.

**Methods:**

GBD 2021 used Japanese data from 1474 sources, covering 371 diseases, including COVID-19, and 88 risk factors. The analysis included estimates of life expectancy, mortality, and disability-adjusted life-years (DALYs). Estimates were generated using the standardised GBD methodology, which incorporates various data sources through statistical modelling, including the Cause Of Death Ensemble Model for mortality, Bayesian Meta-Regression Disease Model for non-fatal outcomes, and risk factor estimation frameworks to quantify attributable burdens. Life expectancy decomposition by cause of death and annualised rates of change of age-standardised mortality and DALYs were calculated for 1990–2005, 2005–15, and 2015–21.

**Findings:**

Between 1990 and 2021, life expectancy in Japan rose from 79·4 years (95% uncertainty interval 79·3–79·4) to 85·2 years (85·1–85·2), with prefecture-level disparities widening. Gains were primarily driven by reduced mortality from stroke (adding 1·5 years to life expectancy), ischaemic heart disease (1·0 years), and neoplasms, particularly stomach cancer (0·5 years), with variation across prefectures. Leading causes of death in 2021 were Alzheimer's disease and other dementias (135·3 deaths [39·5–312·3] per 100 000 population), stroke (114·9 [89·8–129·3] per 100 000), ischaemic heart disease (96·5 [77·7–106·7] per 100 000), and lung cancer (72·1 [61·8–77·5] per 100 000). Age-standardised mortality for major non-communicable diseases declined, but the pace of this decline has slowed. All-cause annualised rate of change in mortality rate decreased from –1·6% for 2005–15 to –1·1% for 2015–21. Age-standardised COVID-19 mortality rates were 0·8 deaths (0·7–0·9) per 100 000 population (accounting for 0·3% of all deaths) in 2020 and 3·0 (2·5–3·7) per 100 000 population in 2021 (1·0% of deaths). Age-standardised DALY rates for diabetes worsened, with annualised rate of change increasing from 0·1% for 2005–15 to 2·2% for 2015–21. This change parallels worsening trends in major risk factors, particularly high fasting plasma glucose (annualised rate of change of attributable DALYs –0·8% for 2005–15 and 0·8% for 2015–21) and high BMI (0·2% and 1·4%, respectively). Age-standardised DALYs attributable to other major risk factors continued to decrease, albeit slower.

**Interpretation:**

Japan's health gains over the past 30 years are now stalling, with rising regional disparities. The increasing burdens of Alzheimer's disease and other dementias and diabetes, alongside high fasting plasma glucose and high BMI, highlight areas needing focused attention and action.

**Funding:**

Gates Foundation.

## Introduction

Over the past 30 years, Japan has made remarkable strides in population health, emerging as one of the world's leading nations in longevity. Older Japanese individuals, in particular, have better health outcomes compared with those in countries with similar socioeconomic status.^1^ Our previous analysis up to 2015, however, revealed a notable slowdown in these advances since 2000.^2^ This slowdown coincides with persistent health disparities across prefectures and a surge in non-communicable diseases (NCDs) associated with an ageing population. The multiphase, long-term, national health promotion initiative, Health Japan 21, implemented by the Ministry of Health, Labour and Welfare, has had a crucial role in addressing these challenges. An assessment of its second phase, using pre-COVID-19 pandemic data, showed notable decreases in age-standardised mortality from major NCDs such as cancer and cardiovascular diseases over nearly a decade.^3^ Nevertheless, there has been little progress in other chronic conditions and in narrowing the health disparities across prefectures. Additionally, challenges such as the scarcity of data on non-fatal disease burdens associated with longevity^4^ and the absence of comprehensive health metrics, which encompass a wide range of health aspects and enable comparative analysis, continue to obstruct the prioritisation of health policies and interventions necessary for revitalising Japan's health progress in a rapidly ageing society where many health issues intersect.^3^

**Research in context**

**Evidence before this study**

Japan has one of the highest life expectancies globally. Analyses up to 2015 indicated continuous health improvements, albeit with a marked deceleration since 2000. Health disparities and non-communicable diseases (NCDs) also increased during this period due to increased longevity and an ageing population. To understand the current landscape, we did a comprehensive search of PubMed Central for articles published from Jan 1, 2020, to July 1, 2024, using the following keywords: “Japan”[abstract] AND “population”[All Fields] AND “diseases”[All Fields] AND “risks”[All Fields] AND “public health”[All Fields] AND (“healthcare system”[All Fields] OR “health care system”[All Fields] OR “health system”[All Fields]). This search yielded 391 articles. These articles revealed a growing interest in understanding the health challenges faced by Japan, particularly in relation to NCDs and the effects of an ageing population. However, existing studies often lack comprehensive, long-term analyses of health trends and disparities across different regions of Japan. There is a clear need for studies that provide a more detailed view of the evolving public health landscape, which this study aims to address.

**Added value of this study**

This study leverages data from the Global Burden of Diseases, Injuries, and Risk Factors Study 2021 to provide an unprecedented overview of the impact of diseases, injuries, and risk factors in Japan and its prefectures from 1990 to 2021, including the initial years of the COVID-19 pandemic. By analysing comprehensive health outcomes and risk factors over three decades, this research offers key insights into mortality and morbidity trends. This study is particularly valuable for policy makers and health-care professionals, as it identifies key areas for intervention and policy development aimed at mitigating disease burden and promoting equitable health improvements across Japan. It underscores the necessity of tailored public health strategies to address the evolving health challenges in an ageing society.

**Implications of all the available evidence**

The findings of this study reveal crucial insights for public health policy and strategy in Japan and other ageing societies. The study highlights a growing burden of NCDs, with notable increases in disability-adjusted life-years for conditions such as Alzheimer's disease and other dementias and diabetes. Persistent health disparities across prefectures and the rising impact of high fasting plasma glucose and as an important risk factor for various health issues are also prominent. Importantly, the study reveals a notable slowdown in health improvements since 2015, particularly in reducing mortality rates for major conditions such as stroke and ischaemic heart disease. This deceleration signals the need for renewed efforts and innovative strategies to revitalise health gains. Socioenvironmental interventions and localised, targeted health strategies tailored to the unique characteristics and needs of each locality are necessary to sustain and enhance health improvements in Japan, addressing the multifaceted challenges posed by an ageing population and evolving health landscape.

With the launch of the third phase of Health Japan 21 in 2024, national and local governments in Japan are formulating new action plans to revamp public health efforts on NCDs and health disparities, particularly adapting to the challenges posed by an increasingly super-aged society. Using the comprehensive framework provided by the Global Burden of Diseases, Injuries, and Risk Factors Study (GBD) 2021, which analysed the impact of diseases and injuries across Japan and its 47 prefectures from 1990 to 2021, we can evaluate various health challenges including those during the early phase of the COVID-19 pandemic. Such an assessment will also be important in objectively considering Japan's response to the pandemic.

This study aims to provide an extensive and long-term assessment of Japan's population health, examining both mortality and morbidity along with broader health trends over the past two decades. By analysing these patterns within the context of Japan's ageing population and evolving health landscape, this study offers valuable insights that will guide the identification of important areas for future interventions and policy enhancements aimed at reducing disease burden and promoting equitable health improvements nationwide, and shares key lessons with the global health community.

This Article was produced as part of the GBD Collaborator Network and in accordance with the GBD Protocol.^5^

## Methods

### Overview

GBD 2021 methodology has been published previously.^6^, ^7^, ^8^, ^9^ We used GBD 2021 data to assess the burden of diseases and injuries in Japan's 47 prefectures from 1990 to 2021, including the COVID-19 pandemic. This latest GBD round represents substantial methodological improvements over previous iterations, including enhanced age-specific data collection for mortality in children younger than 5 years, refined estimation methods for rare causes of death, and, for the first time, the inclusion of COVID-19 burden estimates.^7^ Additionally, risk factor analyses underwent substantial methodological advancement through standardisation of relative risk estimation, implementation of new burden-of-proof risk function methods, improved specification of the mediation matrix, and re-evaluation of theoretical minimum risk exposure levels for 19 risk factors—primarily dietary risks, high systolic blood pressure, high LDL cholesterol, and high BMI.^9^ The analysis included 371 causes and 88 risk factors, using 1474 data sources for Japan. These sources included vital registration systems, national health surveys, surveillance databases, and peer-reviewed literature. Citations and metadata for all data sources used in this study are available in the GBD 2021 Sources Tool. The GBD framework organises causes into communicable, maternal, neonatal, and nutritional diseases; NCDs; and injuries. Risk factors are divided into metabolic, environmental and occupational, and behavioural risks.

We examined several metrics, including mortality, years of life lost (YLLs), years lived with disability (YLDs), disability-adjusted life-years (DALYs), life expectancy at birth, and healthy life expectancy (HALE). Unless noted otherwise, these estimates are reported as the mean value across 500 draws from the distribution, with 95% uncertainty intervals (UIs) calculated as the 2·5th and 97·5th percentile values of these draws, derived through a multistage modelling pipeline. Age-standardised rates were derived from the GBD 2021 standard population structure.^10^ Although our analysis spans 1990–2021, we particularly focus on the 2015–21 period to highlight recent trends and changes since our previous analysis. The three analytical periods (1990–2005, 2005–15, and 2015–21) were chosen to align with notable health policy implementations in Japan and to facilitate comparison with our earlier findings. We calculated the annualised rates of change for age-standardised mortality and DALYs for these periods by measuring the difference in the natural logarithms of the initial and final values over each study period.

This study adheres to the GATHER statement (appendix 1 pp 18–19).^11^

### Mortality estimation

National and prefectural mortality data from 1990 to 2021 were obtained from Japan's vital statistics system. Data were standardised to match the GBD 2021 cause classification, and ill-defined causes were redistributed using established algorithms.^7^ Mortality estimates were generated using the Cause of Death Ensemble model (CODEm), which combines multiple models to enhance predictive validity.^7^ YLLs were calculated by multiplying each death by the GBD 2021 reference life expectancy.^10^

### Non-fatal health outcome estimation

Non-fatal health outcomes were estimated using various data sources, including observational studies, government surveys, and COVID-19 epidemiological studies. Prevalence and incidence were estimated using Bayesian Meta-Regression Disease Model (DisMod-MR 2.1) or spatiotemporal Gaussian process regression (ST-GPR).^8^ DisMod-MR 2.1 integrates data variability and uncertainty, while ST-GPR handles spatial and temporal correlations.^8^ YLDs were calculated by multiplying the prevalence of sequelae by their respective disability weights.^12^

### Calculation of DALYs and HALE

DALYs were computed by summing YLLs and YLDs for each cause. HALE was estimated using age-specific mortality rates and YLDs per capita, following the Sullivan method.^6^

### Effect of the COVID-19 pandemic on disease burden

GBD 2021 estimated several new causes, such as COVID-19 (including post-COVID-19 condition, also known as long COVID) and other COVID-19 pandemic-related outcomes. Drawing from multiple data sources—with Japanese mortality statistics specifically derived from the Ministry of Health, Labour and Welfare's excess mortality research group reports^13^ and non-fatal outcomes from various surveys and peer-reviewed literature—COVID-19 deaths and morbidity were estimated using a susceptible-exposed-infectious transmission model, accounting for factors such as vaccination and antiviral treatments.^8^ YLLs were calculated by multiplying each COVID-19 death by the reference life expectancy.^10^ Non-fatal outcomes included acute sequelae of COVID-19 and long COVID (ie, post-acute sequelae). Daily infections and hospital admissions were tracked to estimate YLDs for acute sequelae, applying corresponding disability weights.^8^ Long COVID prevalence was estimated using DisMod-MR 2.1, and YLDs were calculated based on symptom clusters.^8^ Estimates for other COVID-19 pandemic-related outcomes were derived from an analysis of all-cause excess mortality from 2020 to 2021, using a counterfactual approach.^7^

### Comparative risk assessment

The comparative risk assessment framework estimated the relative risks of outcomes as a function of risk factor exposure. Data were sourced from scientific literature and government statistics. Theoretical minimum risk exposure levels were used to calculate population attributable fractions, which were then applied to the disease burden to estimate attributable burden.^9^

### Decomposition of life expectancy

The decomposition of life expectancy was conducted using a three-step approach designed to quantify the contributions of specific causes of death, explained in detail elsewhere.^7^ The first step involves breaking down the difference in life expectancy by age. The second step further subdivides these contributions by age and cause. Finally, these cause-age-specific contributions are cumulated across age groups to assess the cause-specific impacts on the overall variance in life expectancy.

### Role of the funding source

The funder of the study had no role in study design, data collection, data analysis, data interpretation, or writing of the report.

## Results

All estimates, excluding annualised rate of change analyses, are available for visual exploration via the online visualisation tool GBD Compare, and downloadable format through the GBD Results Tool.

**Figure 1:**
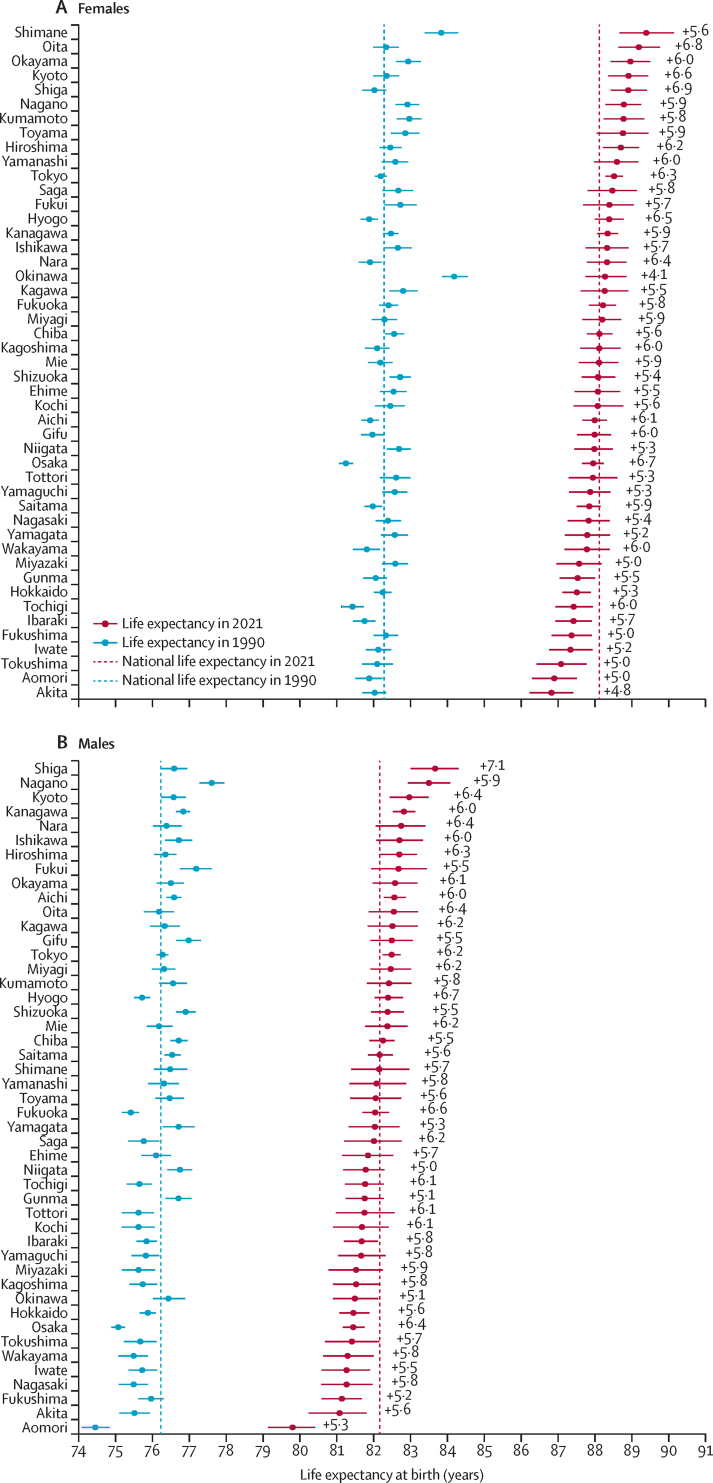
Comparison of life expectancy at birth across 47 prefectures in Japan by sex, 1990 and 2021 Error bars are 95% uncertainty intervals. Prefectures are ordered from top to bottom on the y-axis based on their 2021 life expectancy values (from highest to lowest). The values to the right of each 2021 datapoint show the gain in life expectancy (in years) between 1990 and 2021. See appendix (pp 22–25) for detailed life expectancy values and rankings.

In Japan, the life expectancy at birth in 2021 was 85·2 years (95% UI 85·1–85·2), representing an increase of 5·8 years compared with that in 1990 (79·4 years [79·3–79·4]; appendix 1 pp 3, 20–21). For females, life expectancy reached 88·1 years (88·0–88·2) in 2021, an increase of 5·8 years since 1990 (82·3 years [82·2–82·3]). For males, life expectancy was 82·2 years (82·1–82·2) in 2021, an increase of 5·9 years since 1990 (76·2 years [76·2–76·3]; figure 1; appendix 1 pp 22–25). The disparity in life expectancy between the prefectures with the highest and lowest values increased from 2·3 years in 1990 to 2·9 years in 2021 for all sexes combined; this trend was particularly pronounced in males (from 3·2 years in 1990 to 3·9 years in 2021), whereas in females, the gap slightly narrowed (2·9 years to 2·6 years in females).

**Figure 2:**
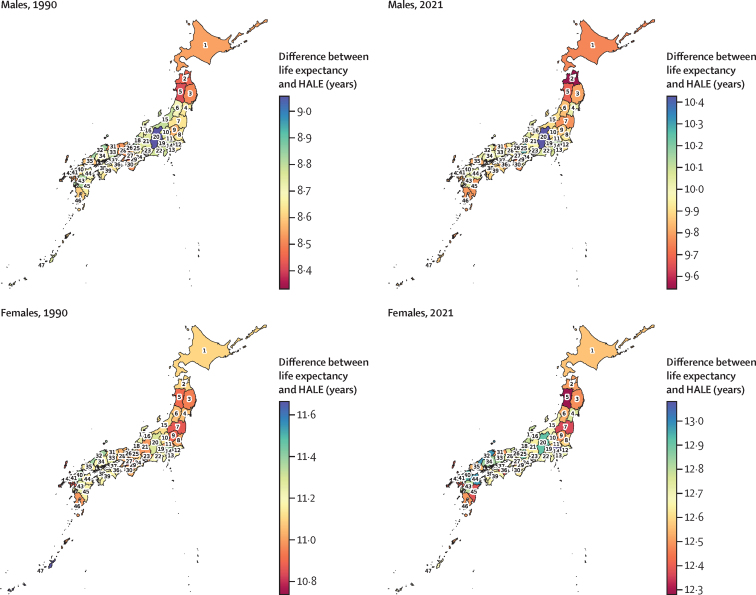
Difference between life expectancy and HALE across 47 prefectures in Japan for males and females, 1990 and 2021 Prefectures are numbered from 1 to 47 according to their ISO codes. 1=Hokkaido. 2=Aomori. 3=Iwate. 4=Miyagi. 5=Akita. 6=Yamagata. 7=Fukushima. 8=Ibaraki. 9=Tochigi. 10=Gunma. 11=Saitama. 12=Chiba. 13=Tokyo. 14=Kanagawa. 15=Niigata. 16=Toyama. 17=Ishikawa. 18=Fukui. 19=Yamanashi. 20=Nagano. 21=Gifu. 22=Shizuoka. 23=Aichi. 24=Mie. 25=Shiga. 26=Kyoto. 27=Osaka. 28=Hyogo. 29=Nara. 30=Wakayama. 31=Tottori. 32=Shimane. 33=Okayama. 34=Hiroshima. 35=Yamaguchi. 36=Tokushima. 37=Kagawa. 38=Ehime. 39=Kochi. 40=Fukuoka. 41=Saga. 42=Nagasaki. 43=Kumamoto. 44=Oita. 45=Miyazaki. 46=Kagoshima. 47=Okinawa. HALE=healthy life expectancy.

HALE rose by 4·4 years, from 69·4 years (66·6–72·0) in 1990 to 73·8 years (70·5–76·7) in 2021 (appendix 1 pp 4, 20–21). For females, HALE reached 75·4 years (71·6–78·7) in 2021, an increase of 4·3 years since 1990 (71·2 years [67·9–74·0]), and for males it reached 72·2 years (69·4–74·7), an increase of 4·7 years since 1990 (67·6 years [65·1–69·7]; appendix 1 pp 5, 22–25). The gap in HALE between the prefectures with the highest and lowest values widened from 1·8 years in 1990 to 2·3 years in 2021, particularly among males (from 2·6 years in 1990 to 3·1 years in 2021, compared with 2·0 years to 2·0 years among females). The difference between life expectancy and HALE increased from a mean of 9·9 years (SD 0·1) to 11·3 years (0·2) across the 47 prefectures during the same period for all sexes combined (appendix 1 p 6). This pattern was consistent in females (from 11·1 years [0·2] to 12·7 years [0·2]) and males (from 8·7 years [0·1] to 9·9 years [0·2]; figure 2).

Geographical patterns in 2021 revealed that western prefectures generally had higher life expectancy and HALE, with Shiga, Kyoto, and Nagano ranking in the top three for both metrics. By contrast, northern prefectures, such as Aomori and Akita, consistently had the lowest values for life expectancy and HALE (appendix 1 p 7). Similar geographical patterns were observed for males and females (appendix 1 pp 8–9).

**Figure 3:**
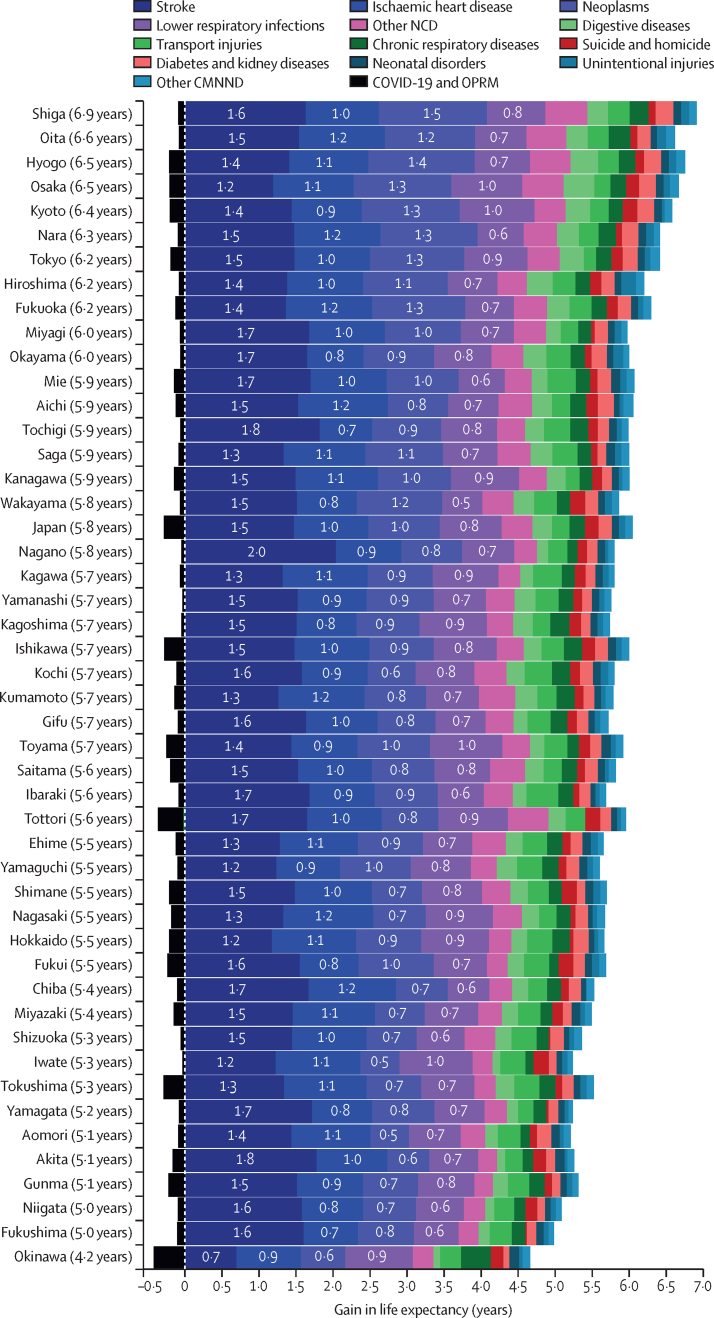
Decomposition of life expectancy changes in Japan by cause of death and prefecture, showing the number of years gained or lost due to each cause for all sexes combined between 1990 and 2021 Prefectures are ordered based on their total gain in life expectancy (shown in parentheses). NCD=non-communicable disease. CMNNDs=communicable, maternal, neonatal, and nutritional diseases. OPRM=other pandemic-related mortality.

The age-standardised mortality rate for all causes decreased by 41·2%, from 529·3 deaths (95% UI 527·5–531·0) per 100 000 population in 1990 to 311·1 (309·5–312·6) per 100 000 population in 2021 (appendix 1 pp 10, 26–27). The rate of decline varied across prefectures, ranging from –49·0% in Shiga to –29·1% in Okinawa. The increase in life expectancy also varied, from 6·8 years in Shiga to 4·2 years in Okinawa (appendix 1 pp 20–21). More than 70% of the increase in life expectancy was contributed by reductions in mortality due to stroke (adding 1·5 years to life expectancy), ischaemic heart disease (1·0 years), neoplasms (1·0 years), and lower respiratory infections (0·8 years; figure 3). Across prefectures, life expectancy gains ranged from 0·7 years to 2·0 years for stroke, from 0·7 years to 1·2 years for ischaemic heart disease, from 0·5 years to 1·5 years for neoplasms, and from 0·5 years to 1·0 years for lower respiratory infections.

**Figure 4:**
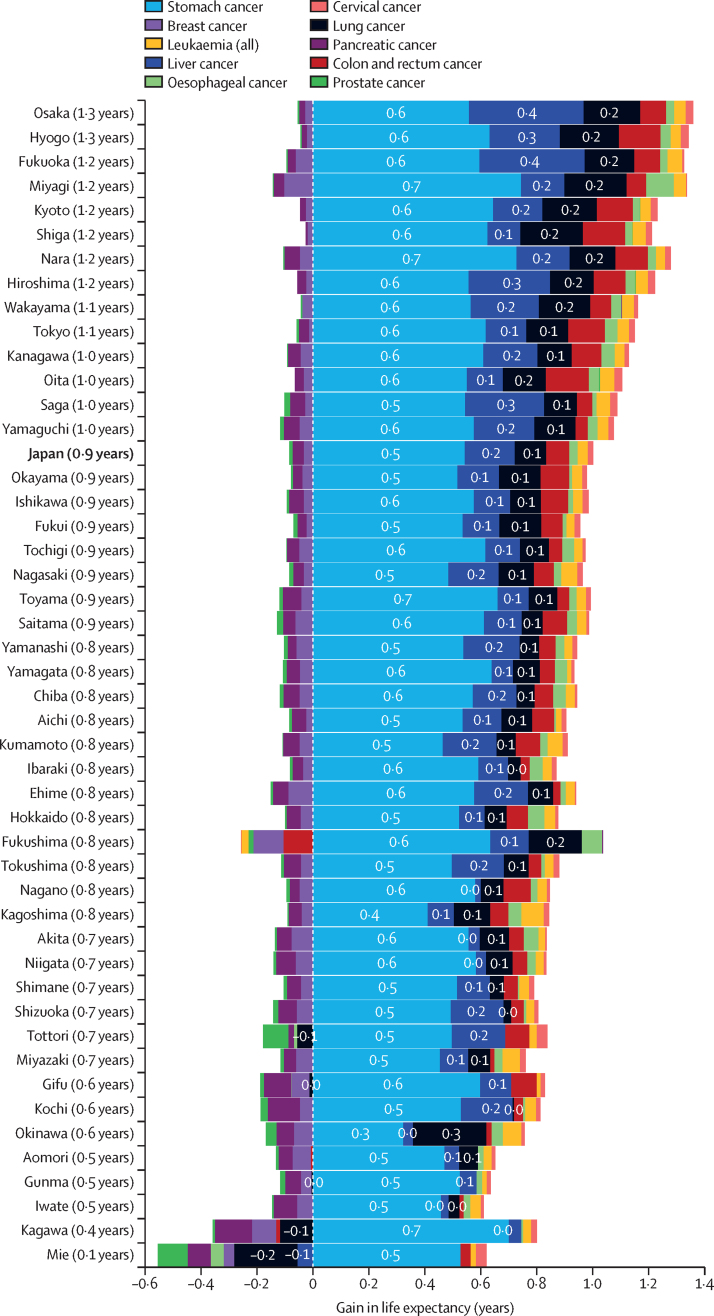
Decomposition of the effect of prevention and treatment of the top 10 cancers on life expectancy in Japan by prefecture for all sexes combined between 1990 and 2021 Prefectures are ordered based on their total gain in life expectancy (shown in parentheses).

Among neoplasms, changes in mortality due to stomach cancer made the largest contribution to reduced mortality, adding 0·5 years to life expectancy, followed by liver cancer (0·2 years) and lung cancer (0·1 years). Prefecture-level variations in these contributions ranged from 0·3 years to 0·7 years for stomach cancer, from –0·1 to 0·4 years for liver cancer, and from –0·2 to 0·3 years for lung cancer (figure 4). This substantial impact of stomach cancer reflects a significant reduction in its age-standardised mortality rate, which declined from 33·8 (95% UI 31·6–34·9) to 13·2 (11·7–14·0) per 100 000 population between 1990 and 2021.

**Figure 5:**
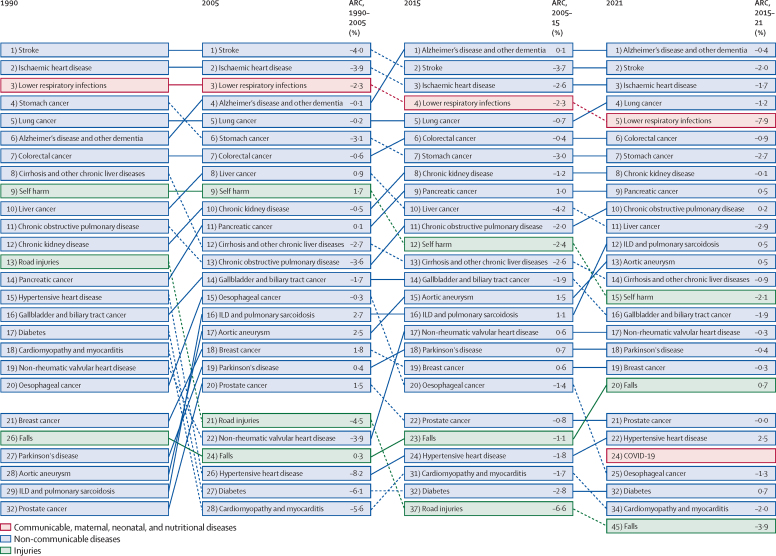
GBD Level 3 causes of death in Japan for the years 1990, 2005, 2015, and 2021 for all ages and sexes, with ARCs for age-standardised mortality rates Rankings are based on the number of deaths from each cause. Data on the number of deaths for each cause are available in GBD Compare. ARC=annualised rate of change. GBD=Global Burden of Diseases, Injuries, and Risk Factors Study. ILD=interstitial lung disease.

In 2021, the five leading causes of death in Japan were Alzheimer's disease and other dementias (135·3 deaths [95% UI 39·5–312·3] per 100 000 population), stroke (114·9 [89·8–129·3] per 100 000), ischaemic heart disease (96·5 [77·7–106·7] per 100 000), lung cancer (72·1 [61·8–77·5] per 100 000), and lower respiratory infections (62·3 [49·9–69·2] per 100 000). Mortality rates for other causes are available in GBD Compare. Four of these causes have maintained their position among the top five since 1990, while Alzheimer's disease and other dementias rose from sixth position to first position between 1990 and 2021 (figure 5). Of the total deaths in 2021, Alzheimer's disease and other dementias accounted for 12·0% (3·5–27·7), stroke for 10·2% (8·0–11·5), ischaemic heart disease for 8·6% (6·9–9·5), lung cancer for 6·4% (5·5–6·9), and lower respiratory infections for 5·5% (4·4–6·2). Data on the percentage of deaths by other causes are available in GBD Compare.

Analysis of temporal trends revealed a progressive deceleration of the decline in age-standardised mortality rate for all causes, with an annualised rate of change of –2·0% from 1990 to 2005, –1·6% from 2005 to 2015, and –1·1% from 2015 to 2021. A similar pattern of deceleration was observed for stroke (annualised rate of change –4·0% for 1990–2005, –3·7% for 2005–15, and –2·0% for 2015–21) and ischaemic heart disease (–3·9%, –2·6%, and –1·7%; figure 5). Conversely, the annualised rate of change for lung cancer showed progressive improvement over the same periods (–0·2%, –0·7%, and –1·2%). Decelerations in improvements in age-standardised mortality rate were observed in females (–2·5%, –1·5%, and –1·2%) and males (–1·6%, –1·9%, and –1·2%) across the three periods (appendix 1 pp 11–12).

COVID-19 was responsible for 0·3% of total deaths in 2020 (age-standardised mortality rate 0·8 deaths [95% UI 0·7–0·9] per 100 000 population), and 1·0% of total deaths in 2021 (3·0 [2·5–3·7] per 100 000). COVID-19 mortality rates varied among prefectures; in 2020, the age-standardised COVID-19 mortality rate ranged from 0·0 (0·0–0·0) in Shimane to 2·2 (1·0–3·4) in Hokkaido; and in 2021, the range extended from 0·2 (0·1–0·4) in Tottori to 8·8 (3·6–16·9) in Okinawa (appendix 1 pp 13, 28–29).

The age-standardised DALY rate for all causes decreased by 24·5%, from 21 449·6 DALYs (95% UI 19 032·9–24 332·4) per 100 000 population in 1990 to 16 186·7 (13 729·3–18 997·2) per 100 000 in 2021 (appendix 1 pp 26–27). The rate of decline varied among prefectures, ranging from –27·7% in Shiga to –19·6% in Okinawa. The proportion of the age-standardised DALY rate attributable to YLDs increased from 45·0% in 1990 to 58·8% in 2021.

In 2021, the leading causes of DALYs in Japan were stroke (2016·2 DALYs [95% UI 1758·8–2224·4] per 100 000 population), Alzheimer's disease and other dementias (1988·5 [947·3–3952·2] per 100 000), low back pain (1748·4 [1245·9–2345·5] per 100 000), ischaemic heart disease (1412·0 [1218·9–1519·6] per 100 000), and lung cancer (1166·3 [1040·5–1234·3] per 100 000). Of the total DALYs, stroke accounted for 6·6% (5·6–7·4), Alzheimer's disease and other dementias for 6·5% (3·1–13·1), low back pain for 5·7% (4·5–7·0), ischaemic heart disease for 4·6% (3·9–5·4), and lung cancer for 3·8% (3·2–4·4). Alzheimer's disease and other dementias rose in rank from 12th position in 1990 to second in 2021 (appendix 1 p 14). Data on DALY rates and percentages by other causes are available in GBD Compare.

The decline in age-standardised DALY rates in Japan nationally has also slowed, with an annualised rate of change of –1·0% for 1990–2005, –1·0% for 2005–15, and –0·5% for 2015–21. The declines in DALY rates for stroke (annualised rate of change –3·1%, –3·0%, and –1·3%) and ischaemic heart disease (–3·0%, –2·6%, and –1·6%) have also decelerated (appendix p 14). Conversely, the decline in DALY rates for Alzheimer's disease and other dementias (0·0%, 0·1%, and –0·5%), low back pain (–0·3%, –0·0%, and –0·7%), and lung cancer (–0·4%, –1·1%, and –1·7%) has accelerated in recent years. Age-standardised DALY rates for diabetes have worsened, with the rate of deterioration increasing (annualised rate of change 0·4%, 0·1%, and 2·2%). The deceleration in improvement in age-standardised DALY rate was observed in females (all-cause annualised rate of change –1·0%, –0·7%, and –0·3%) and males (–1·0%, –1·3%, and –0·7%) across the three periods (appendix 1 pp 15–16).

All 88 risk factors assessed in GBD 2021 explained 41·9% (95% UI 35·4–47·0) of total deaths in Japan nationally in 2021. Metabolic risk factors accounted for 24·9% (19·7–29·8) of total deaths, behavioural risk factors for 21·6% (15·7–27·0), and environmental and occupational risks for 9·1% (6·9–11·0). Similarly, all risk factors jointly explained 34·4% (30·3–38·4) of total DALYs. Nationally, metabolic risk factors contributed more to DALYs in 2021 (19·8% [15·9–23·4] of total DALYs) than behavioural risk factors (18·3% [14·4–21·9]) or environmental and occupational risks (6·9% [5·6–8·2]).

From 1990 to 2021, the age-standardised mortality rates and DALY rates attributable to metabolic risks, such as high systolic blood pressure, and behavioural risks, such tobacco use and dietary risks (eg, high sodium intake and low fruit consumption), have declined, although this trend has slowed recently. Conversely, the rates due to high BMI have worsened. For age-standardised mortality rate, the annualised rate of change related to high BMI was –2·3% for 1990–2005, –0·7% for 2005–15, and 0·7% for 2015–21. For age-standardised DALY rate, the annualised rates of change related to high BMI were –0·3%, 0·2%, and 1·4% for the respective periods. Additionally, while age-standardised mortality rates attributable to high fasting plasma glucose have stagnated (annualised rates of change –2·6%, –1·9%, and –0·6%), age-standardised DALY rates have recently worsened (–0·7%, –0·8%, and 0·8%; appendix 1 p 17).

## Discussion

Over the past three decades, Japan has made notable health gains, with life expectancy increasing by 5·8 years between 1990 and 2021, surpassing the 4·5 year increase in the high-income super-region.^10^ However, this gain has not translated proportionally to HALE, as shown by the widening gap between HALE and life expectancy. Disparities in life expectancy and HALE persist among prefectures and between sexes. While our 2015 analysis identified these regional variations,^2^ the current study, using GBD 2021's enhanced methodology, confirms their continuation throughout 2021. These complex regional variations are not solely attributable to health system inputs or risk factors,^2^ but reflect complex interactions between health system performance and social determinants of health,^14^, ^15^ including economic and educational disparities and health-care resource distribution.^14^ Addressing these disparities is essential for ensuring nationwide health equity.^16^

Our decomposition analysis reveals varying contributions of disease-specific mortality reductions to life expectancy gains across prefectures. The most substantial impacts came from reduced mortality in stroke, ischaemic heart disease, neoplasms, and lower respiratory infections, but their contributions differed markedly by prefecture. These persistent regional differences indicate the need for localised health interventions.

Among neoplasms, stomach cancer emerges as a major contributor to increased life expectancy, showing substantial mortality decline despite regional variations. This reduction stems from decreased *Helicobacter pylori* infection, reduced dietary salt intake and improved food preservation methods, enhanced preventive measures, and advanced early detection since 2000.^17^ Japan's progress in stomach cancer control is notable: Japan's age-standardised mortality rate dropped from 33·8 to 13·2 per 100 000 population between 1990 and 2021, approaching global (11·2 [95% UI 9·6–12·7] per 100 000) and high-income super-region (6·6 [5·9–7·0] per 100 000) rates.^7^ Now ranking third among cancer deaths in Japan, after lung cancer (21·3 per 100 000) and colorectal cancer (15·9 per 100 000), stomach cancer's age-standardised mortality rate continues to decline more rapidly than other major causes since 2015, suggesting further improvements ahead.

As Japan enters the third phase of Health Japan 21, a national long-term health strategy targeting NCDs that was initiated in 2024, reducing inter-prefectural health disparities takes priority. Our findings of persistent regional variations underscore the urgency of this focus. While maintaining support for individual lifestyle improvements, the new phase further strengthens its commitment to enhancing supportive social environments for health.^18^ It targets structural causes of health inequalities through comprehensive engagement of stakeholders, including local governments, the private sector, academia, educational institutions, and civil society organisations. The strategy emphasises locally tailored approaches and enhanced governance.

The third phase of Health Japan 21 strengthens corporate health management, supporting employee health and awareness through company-led initiatives with local government backing.^18^ This approach aims to extend health promotion throughout communities. It also advocates for improved nutrition management in institutional settings, such as workplaces, schools, health-care facilities.^18^ Japanese and international studies show that enhanced workplace meal quality improves dietary habits and can help prevent obesity and related conditions.^19^, ^20^

Prefectures should lead in strengthening stakeholder collaboration. Beyond individual behaviour indicators, it is essential to collect, analyse, and utilise data on health disparities caused by social determinants and on community health-supporting environments. Aligned with national goals, prefectures should formulate and implement health promotion plans using the Plan-Do-Check-Act cycle to address inter-prefectural and inter-municipal disparities.^21^

Previous research up to 2015 highlighted the slowdown in health improvements in Japan.^2^ Our study reveals that this trend intensified from 2015 to 2021 for all sexes, particularly for stroke and ischaemic heart disease. Underlying causes might include deteriorating conditions such as diabetes, high fasting plasma glucose, and high BMI (overweight and obesity), as well as regional health-care disparities. However, specific mechanisms remain unclear. While this slowdown could indicate an approach towards an equilibrium given current health science and technological capabilities, rather than system failure, future research should prioritise identifying factors contributing to slowing health improvement to inform policy decisions on preventive care and treatment.

Analyses of DALY rates between 2015 and 2021 reveal the persistent burden of NCDs. Dementia and diabetes show particularly concerning trends. The DALY rate due to Alzheimer's disease and other dementias rose 19·5% between 2015 and 2021 in Japan, becoming a pressing concern for health in adults. Projections indicate a 44·9% increase (2022–50), making it the leading cause of DALYs in Japan by 2050.^22^ Per capita health spending on these conditions in high-income countries was estimated at approximately US$207·3 in 2019, with projections suggesting it could potentially reach around $696·5 by 2050.^23^ Prevention and management require a multifaceted approach: promoting physical activity, social participation, early diagnosis and treatment, maintaining sensory functions,^24^ reducing caregiver strain, and enhancing family support.^25^

The growing burden of age-related conditions presents substantial challenges for health-care systems, particularly regarding resource allocation and service delivery. Given Japan's rapidly ageing demographic structure, the country has been developing innovative approaches to health-care delivery and financing. As a pioneering nation in addressing age-associated conditions, Japan's experiences could influence global practices.^26^ A key initiative has been the Community-based Integrated Care System (CICS), implemented by local authorities since 2006, which delivers a continuum of services within community settings.^27^ CICS encompasses tailored residential environments, social support, daily activity assistance, preventive health measures, medical treatment, and nursing care. This comprehensive system addresses ageing-related challenges through integrated health-care delivery and resource allocation. The model enables coordinated interventions for maintaining and enhancing health, promoting community participation to mitigate risks for Alzheimer's disease, and addressing age-related issues.^28^ Recent studies have shown the potential effectiveness of community organising approaches within CICS.^29^, ^30^ These approaches, involving intersectoral partnerships, show promising results in improving health outcomes and social participation among older adults.

Some cancers (eg, lung, stomach, and liver) show stable or improving disease burdens due to declining mortality rates, driven by infection prevention, enhanced early detection, and therapeutic advances.^17^ Conversely, the DALY rate for pancreatic cancer increased by 11·3% between 2015 and 2021 due to difficult early detection and limited treatment progress.^31^ Urgent needs include developing early diagnosis methods, mitigating modifiable risk factors (eg, smoking, high fasting plasma glucose, and high BMI), and exploring new treatments. Artificial intelligence shows promise for enhancing early detection, but requires strategic collaboration and funding.

Risk factor analysis revealed several concerning metabolic risks. While some risks (high systolic blood pressure, smoking, and unhealthy diet) are decreasing after age-standardisation, others, such as high fasting plasma glucose and high BMI, are increasing. Worsening DALYs for diabetes highlight the need to strengthen measures against lifestyle-related diseases, particularly blood glucose management.^32^ Our study revealed a notable 11·4% increase in DALY rate due to high BMI among adults aged 70 years and older from 2015 to 2021, underscoring the need to address age-specific nutritional challenges such as sarcopenic obesity. These challenges persist in Health Japan 21's third phase,^3^ with nutrition management crucial in preventing sarcopenia and frailty, affecting up to 47% of older hospitalised patients worldwide.^33^

Our results have highlighted the rising COVID-19 burden in Japan from 2020 to 2021. However, despite this burden, Japan maintained one of the lowest COVID-19 mortality rates globally by the end of 2021. The age-standardised mortality rate remained low at 3·0 (95% UI 2·5–3·7) per 100 000 population in 2021, markedly lower than the global rate of 94·0 (89·5–100·1) per 100 000 and high-income super-region rate of 48·1 (47·3–48·9) per 100 000.^12^

The reasons are debated. By late 2021, Japan, along with other east Asian countries such as South Korea and Singapore, maintained low COVID-19 mortality with high face mask usage (93% in Japan *vs* 59% globally)^34^ and extensive vaccination coverage (80% with two doses in Japan *vs* 49% globally).^35^, ^36^ Japan's healthier population profile, particularly its low obesity rates, and adherence to infection control measures might contribute.^37^ Notably, infection control was achieved without strict lockdowns or mandates. The culture of prosociality, which prioritises social conformity and collective welfare, might have facilitated adherence to pandemic measures.^38^ While our GBD 2021 data analysis provides valuable insights into the initial pandemic phase, assessing the full impact of COVID-19 in Japan would require data beyond 2021, given the substantial surge after 2022 driven by the omicron variant (B.1.1.529 and its descendent lineages).

Despite Japan's relatively effective containment of direct COVID-19 mortality throughout 2021, the pandemic had notable indirect consequences, particularly on mental health. GBD 2021 data reveal a substantial increase in mental disorders, especially among younger generations and women.^39^ Among people aged 10–54 years, DALYs rate due to mental disorders increased by 15·6% for women and 9·0% for men from 2019 to 2021.^8^ These trends likely reflect the psychological effects of social isolation and pandemic-related stressors,^40^ contributing to increased post-pandemic suicide rates.^41^

GBD DALY estimates for specific diseases and injuries have limitations, as explained elsewhere.^8^ GBD 2021 faced challenges in modelling COVID-19 due to varying data quality. A limitation is GBD 2021's omission of COVID-19 risk factor attribution, likely underestimating the overall risk burden. This is notable given the evidence linking pre-existing risk factors to higher COVID-19 mortality rates.^42^ Additionally, while emerging challenges such as antimicrobial resistance and the health impacts of climate change could substantially affect Japan's future health, the GBD 2021 framework does not classify these as distinct causes or risk factors, limiting comparative analysis.

Limitations from Japan's GBD 2015 study persist, despite methodological improvements.^2^ Data gaps remain at both the national and prefectural levels. GBD disease models compensate by using regional data and covariates, potentially resulting in minimal regional variation in certain diseases and risk factors due to limited Japanese data coverage.

Data quality issues affect non-fatal outcome measurements. For instance, Japanese depression and anxiety data primarily come from online surveys, which, while regionally representative, exclude individuals living in institutions. Furthermore, incomplete temporal coverage means 2021 estimates relied heavily on GBD disease models.

Differences between GBD estimates and national statistics stem from varying disease definitions, coding practices, and cause-of-death attribution methodologies.^7^ While Japan maintains robust national statistics, GBD's standardised approach enables consistent international comparisons and systematic uncertainty quantification.

GBD 2021's disability weights, crucial for YLD estimation, are globally uniform but based on limited sampling. The original disability weight study included surveys from nine countries and web-based responses primarily from non-East Asian nations.^43^, ^44^ Studies in Japan and China revealed cultural variations in disability weights compared with GBD values.^45^, ^46^ For instance, Japan showed lower weights for mental disorders and alcohol use disorders but higher weights for pain and sensory symptoms.^45^ The GBD 2021 does not account for this heterogeneity.

Japan's health achievements over the past three decades, marked by reductions in mortality from stroke, ischaemic heart disease, and cancers, including remarkable successes in preventing and treating stomach cancer, are notable. However, health improvements have recently slowed, alongside widening health disparities and rising burdens of Alzheimer's disease and other dementias and diabetes, compounded by increasing high fasting plasma glucose and high BMI. Addressing these challenges in the next decade is crucial to continue improving population health in Japan. Current policy is targeted to achieve that through socioenvironmental interventions and localised, targeted health strategies, which are essential to sustain and enhance health improvements in Japan.

### GBD 2021 Japan Collaborators

### Contributors

### Data sharing

Data used in the analyses of this manuscript are available publicly to download. Please visit the Global Health Data Exchange GBD 2021 website.

## Declaration of interests

S Bhaskar reports grants or contracts from the Japan Society for the Promotion of Science, Japanese Ministry of Education, Culture, Sports, Science and Technology, and from The Australian Academy of Science; leadership or fiduciary roles in board, society, committee, or advocacy groups (paid or unpaid) as the Visiting Director in the Department of Neurology at the National Cerebral and Cardiovascular Center, Suita (Osaka, Japan), as District Chair of diversity, equity and inclusion at the Rotary District 9675, as Chair and Manager of the Global Health and Migration Hub Community (Berlin, Germany), as an editorial member of *PLoS One, BMC Neurology, Frontiers in Neurology, Frontiers in Stroke, Frontiers in Aging, Frontiers in Public Health*, and *BMC Medical Research Methodology*, as a member of the College of Reviewers (Canadian Institutes of Health Research, Government of Canada), a member of the Scientific Review Committee at Cardiff University Biobank (UK), an expert advisor and reviewer with the Cariplo Foundation (Milan, Italy), and as Visiting Director at the National Cerebral and Cardiovascular Center, Department of Neurology, Division of Cerebrovascular Medicine and Neurology (Suita, Osaka, Japan); outside the submitted work. N Kawakami reports grants or contracts from Junpukai Foundation and the Department of Digital Mental Health (The University of Tokyo); consulting fees from SB@WORK; leadership or fiduciary role in other board, society, committee, or advocacy group (unpaid) with the Japan Society for Occupational Health; outside the submitted work. T Lallukka reports support for the present manuscript from the Research Council of Finland (330527). M Lee reports support for the present manuscript from the Ministry of Education of the Republic of Korea and the National Research Foundation of Korea (NRF-2023S1A3A2A05095298). S Nomura reports support for the present manuscript from Precursory Research for Embryonic Science and Technology from the Japan Science and Technology Agency (JPMJPR22R8) and the National Cancer Center Research and Development Fund (2024-A-14). B Oancea reports grants or contracts from the MRID, project PNRR-I8 number 842027778, contract number 760096; outside the submitted work. T Tabuchi reports grants or contracts from Daiichi Sankyo Healthcare and consulting fees from Daiichi Sankyo Healthcare, outside the submitted work. Y Yasufuku reports grants or contracts from Shionogi & Co, outside the submitted work.
