# Supplementary material for: Three decades of population health changes in Japan, 1990–2021: a subnational analysis for the Global Burden of Disease Study 2021
Source: Lancet Public Health. 2025 Mar 20;10(4):e321–32. doi: 10.1016/S2468-2667(25)00044-1 (PMC11959113; doi:10.1016/S2468-2667(25)00044-1)
Supplement: Supplementary appendix 1 [file mmc1.pdf]

THE LANCET
Public Health

Supplementary appendix 1

This appendix formed part of the original submission and has been peer reviewed. We post it as supplied by the authors.

Supplement to: GBD 2021 Japan Collaborators. Three decades of population health changes in Japan, 1990–2021: a subnational analysis for the Global Burden of Disease Study 2021. *Lancet Public Health* 2025; published online March 20. https://doi.org/10.1016/S2468-2667(25)00044-1.

Table of Contents

Supplementary Figures **3**

Figure S1: Comparison of life expectancy across 47 prefectures in Japan in 1990 and 2021 for both sexes 3

Figure S2: Comparison of healthy life expectancy across 47 prefectures in Japan in 1990 and 2021 for both sexes 4

Figure S3: Comparison of healthy life expectancy across 47 prefectures in Japan in 1990 and 2021 for (A) females and (B) males 5

Figure S4: Difference between life expectancy and healthy life expectancy across the 47 prefectures in (A) 1990 and (B) 2021 for both sexes 6

Figure S5: Maps of life expectancy and healthy life expectancy across 47 prefectures in Japan in 1990 and 2021 for both sexes 7

Figure S6: Maps of life expectancy and healthy life expectancy across 47 prefectures in Japan in 1990 and 2021 for females 8

Figure S7: Maps of life expectancy and healthy life expectancy across 47 prefectures in Japan in 1990 and 2021 for males 9

Figure S8: Comparison of all-cause age-standardised mortality rates across 47 prefectures in Japan in 1990 and 2021 for both sexes 10

Figure S9: GBD level 3 causes of death in Japan for the years 1990, 2005, 2015, and 2021 for all ages and females, with annualised rates of change (ARC) for age-standardised mortality rates 11

Figure S10: GBD level 3 causes of death in Japan for the years 1990, 2005, 2015, and 2021 for all ages and males, with annualised rates of change (ARC) for age-standardised mortality rates 12

Figure S11: Comparison of COVID-19 age-standardised mortality rates across 47 prefectures in Japan in (A) 2020 and (B) 2021 for both sexes 13

Figure S12: GBD level 3 causes of disability-adjusted life years (DALYs) in Japan for the years 1990, 2005, 2015, and 2021 for all ages and both sexes, with annualised rates of change (ARC) for age-standardised DALY rate 14

Figure S13: GBD level 3 causes of disability-adjusted life years (DALYs) in Japan for the years 1990, 2005, 2015, and 2021 for all ages and females, with annualised rates of change (ARC) for age-standardised DALY rate....15

Figure 14: GBD level 3 causes of disability-adjusted life years (DALYs) in Japan for the years 1990, 2005, 2015, and 2021 for all ages and males, with annualised rates of change (ARC) for age-standardised DALY rate....16

Figure S15: GBD level 3 risk factors for (A) mortality and (B) DALYs in Japan for the years 1990, 2005, 2015, and 2021 for all ages and both sexes, with annualised rates of change (ARC) for age-standardised rates 17

Supplementary Tables 18

Table S1: GATHER checklist....18

Table S2: Life expectancy and healthy life expectancy at birth (years) in Japan and 47 prefectures, 1990 and 2021, for both sexes 20

Table S3: Life expectancy and healthy life expectancy at birth (years) in Japan and 47 prefectures, 1990 and 2021, for females 22

Table S4: Life expectancy and healthy life expectancy at birth (years) in Japan and 47 prefectures, 1990 and 2021, for males....24

Table S5: Age-standardised mortality and DALY rates per 100,000 population in Japan and 47 Prefectures: 1990 and 2021 values with percent change (%), for both sexes....26

Table S6: COVID-19 mortality rate, percentage of total deaths, and COVID-19 age-standardised mortality rate in Japan and 47 prefectures, 2020 and 2021, for both sexes....28

Table S7: COVID-19 DALY rate, percentage of total DALYs, and COVID-19 age-standardised DALY rate in Japan and 47 prefectures, 2020 and 2021, for both sexes....29

Supplementary Figures

Figure S1: Comparison of life expectancy across 47 prefectures in Japan in 1990 and 2021 for both sexes

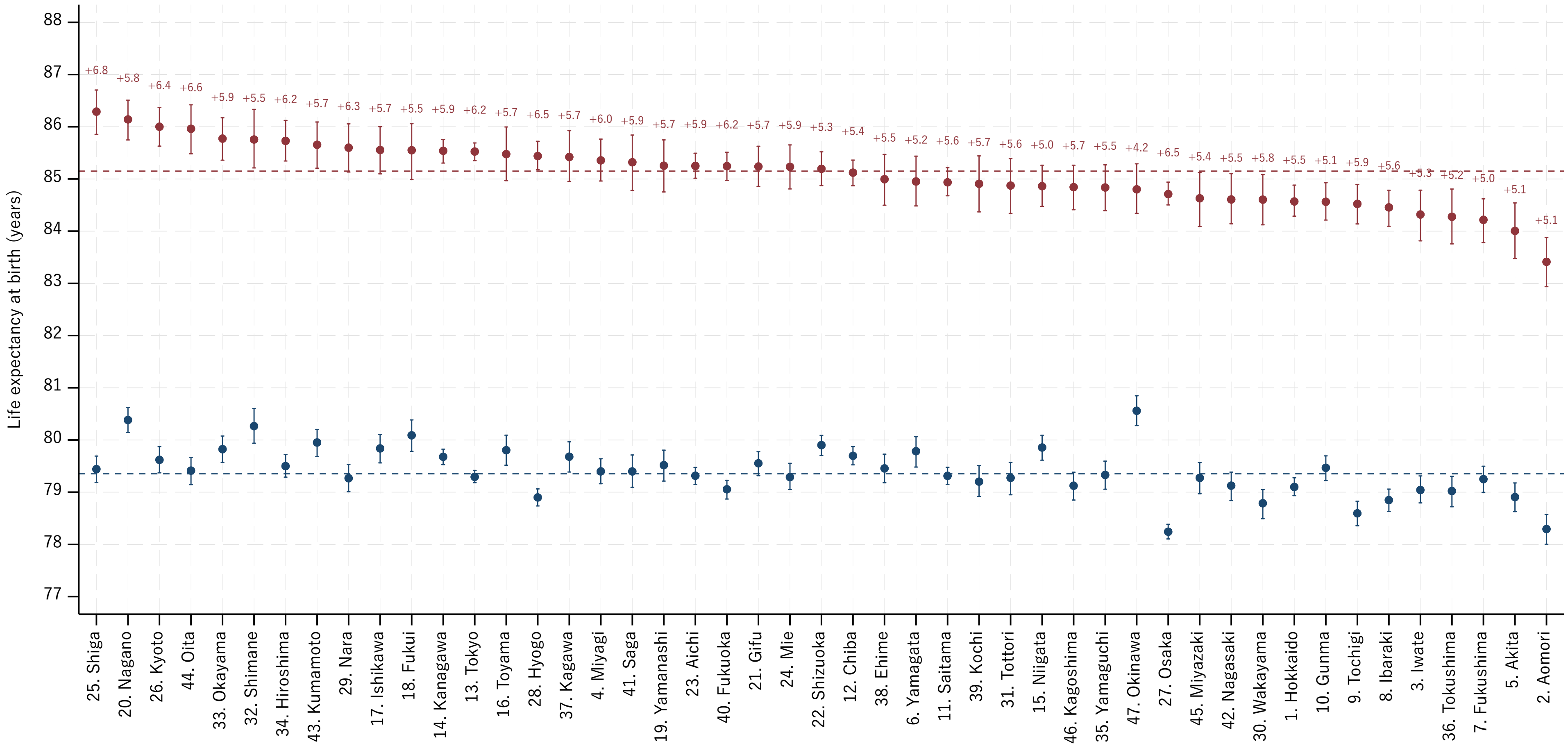

The error bars represent 95% uncertainty intervals. The red line indicates data for 2021, while the blue line represents 1990. The dotted lines show the national life expectancy at birth (79.4 years in 1990 and 85.2 years in 2021). The prefectures are ordered based on their 2021 life expectancy values, from highest to lowest, and are numbered from 1 to 47 according to their ISO codes. The positive values within the figure represent the gain between 1990 and 2021.

Figure S2: Comparison of healthy life expectancy across 47 prefectures in Japan in 1990 and 2021 for both sexes

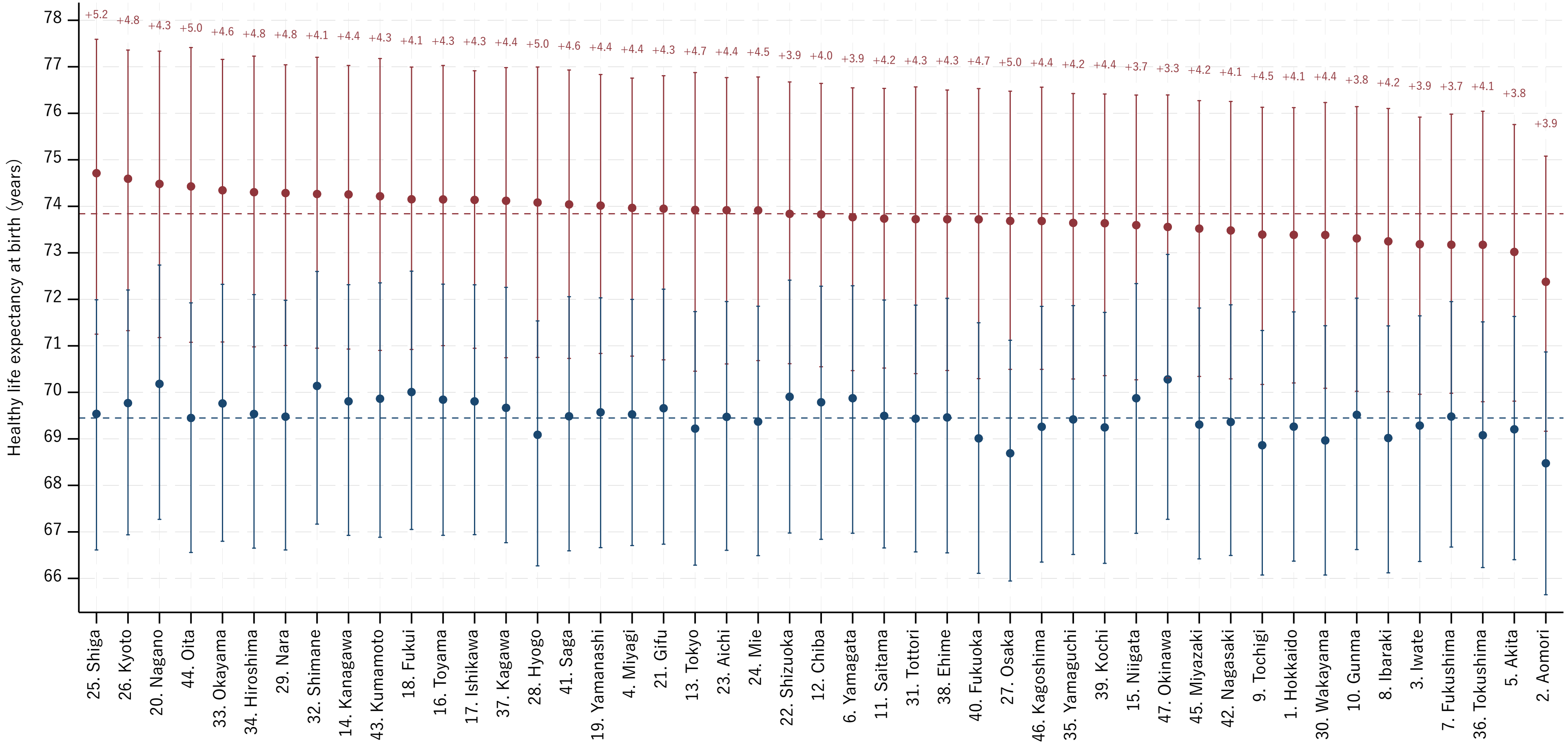

The error bars represent 95% uncertainty intervals. The red line indicates data for 2021, while the blue line represents 1990. The dotted lines show the national healthy life expectancy at birth (69.5 years in 1990 and 73.8 years in 2021). The prefectures are ordered based on their 2021 healthy life expectancy values, from highest to lowest, and are numbered from 1 to 47 according to their ISO codes. The positive values within the figure represent the gain between 1990 and 2021.

Figure S3: Comparison of healthy life expectancy across 47 prefectures in Japan in 1990 and 2021 for (A) females and (B) males

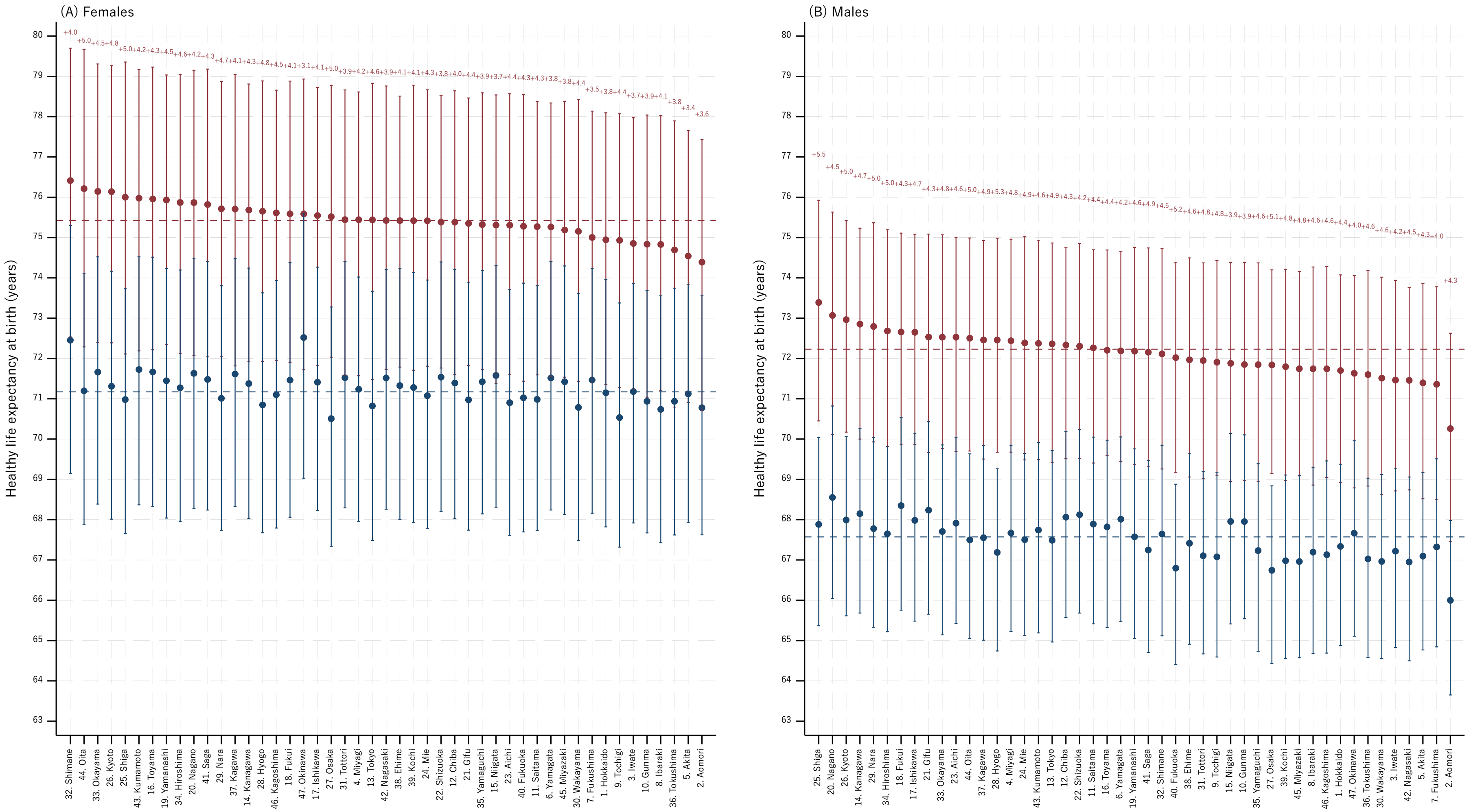

The error bars represent 95% uncertainty intervals. The red line indicates data for 2021, while the blue line represents 1990. The dotted lines show the national healthy life expectancy at birth (females: 71.2 years in 1990 and 75.4 years in 2021; males: 67.6 years in 1990 and 72.2 years in 2021). The prefectures are ordered based on their 2021 healthy life expectancy values, from highest to lowest, and are numbered from 1 to 47 according to their ISO codes. The positive values within the figure represent the gain between 1990 and 2021.

Figure S4: Difference between life expectancy and healthy life expectancy across the 47 prefectures in (A) 1990 and (B) 2021 for both sexes

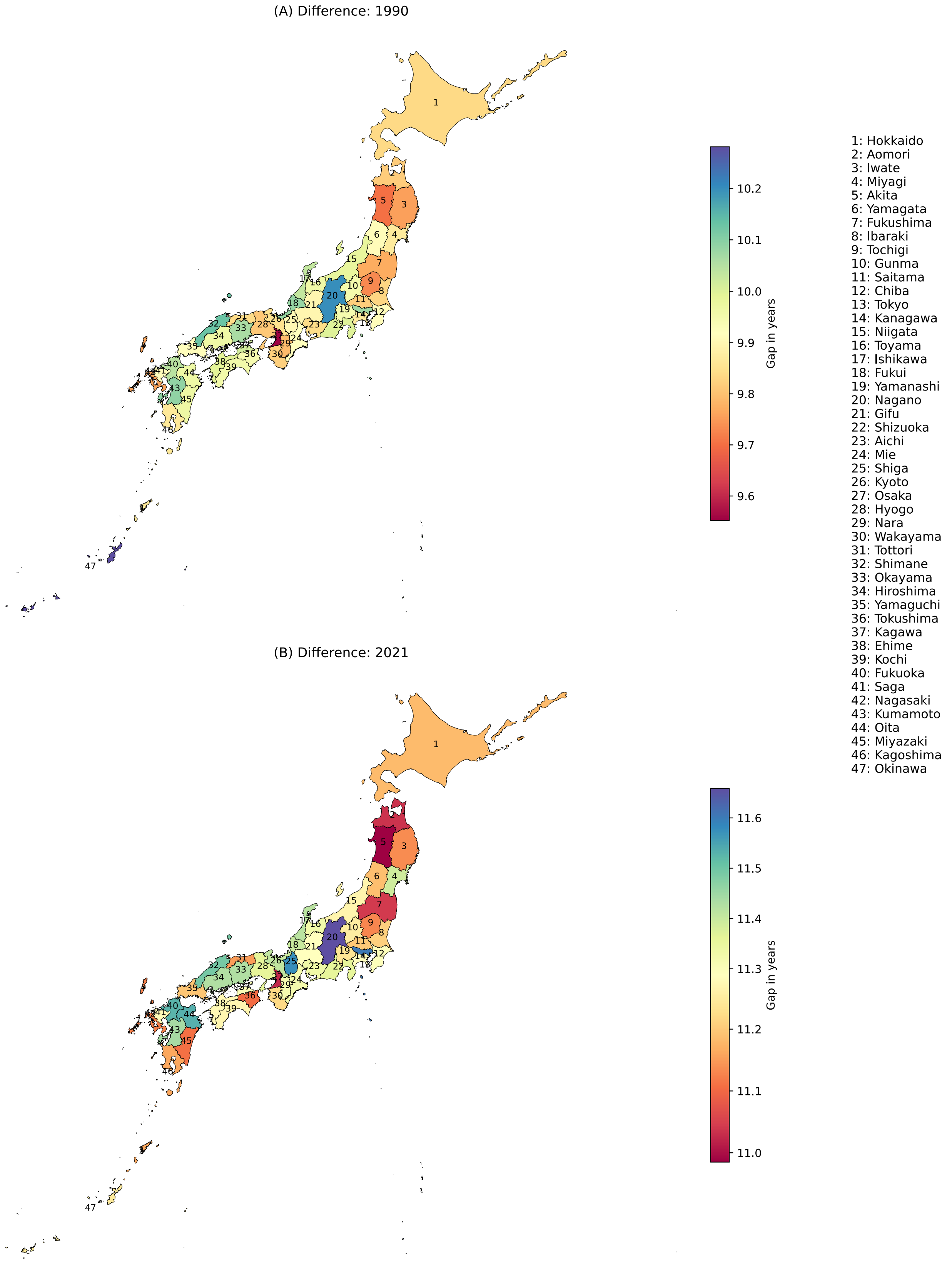

Pefectures are numbered from 1 to 47 according to their ISO codes.

Figure S5: Maps of life expectancy and healthy life expectancy across 47 prefectures in Japan in 1990 and 2021 for both sexes

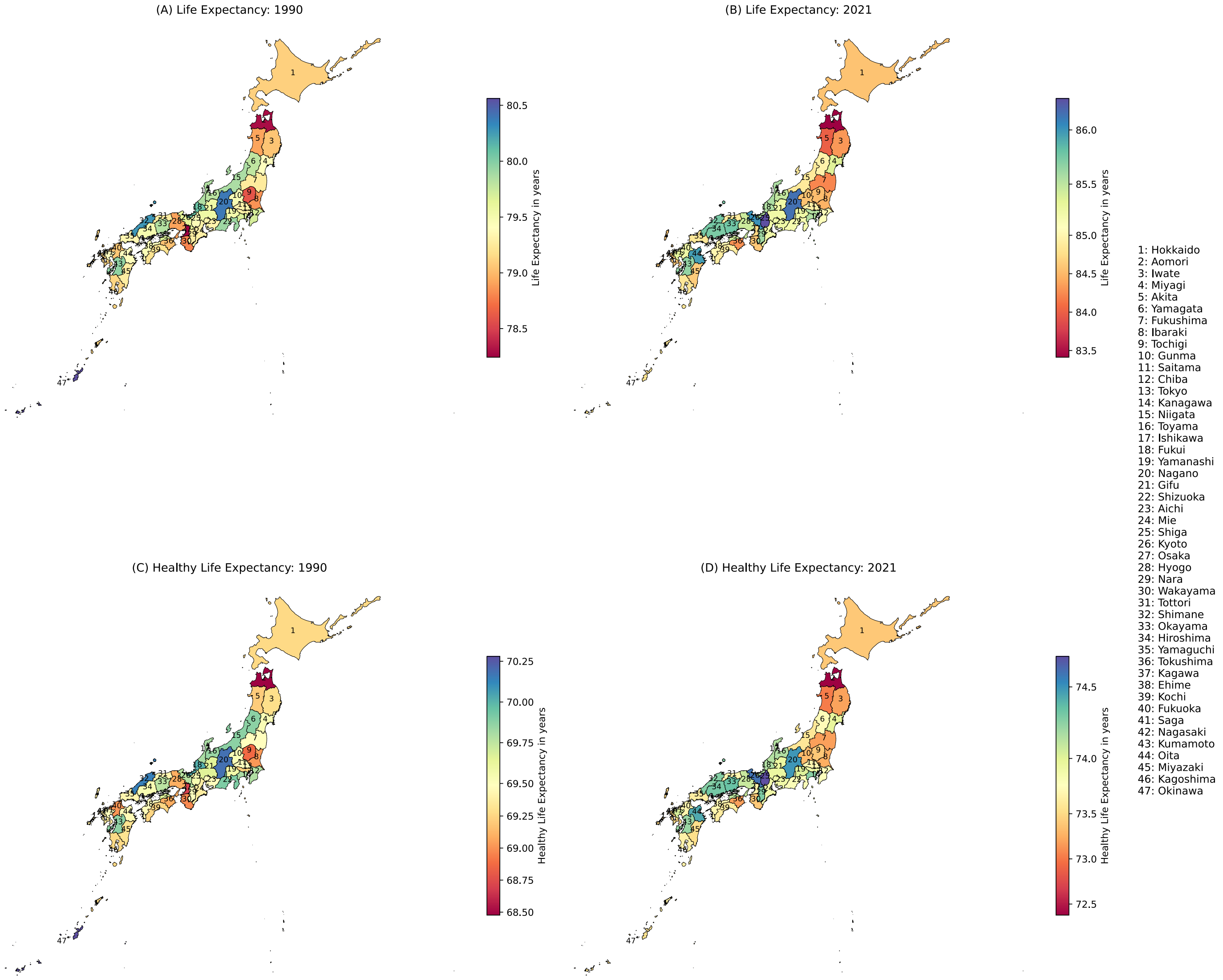

Pefectures are numbered from 1 to 47 according to their ISO codes.

Figure S6: Maps of life expectancy and healthy life expectancy across 47 prefectures in Japan in 1990 and 2021 for females

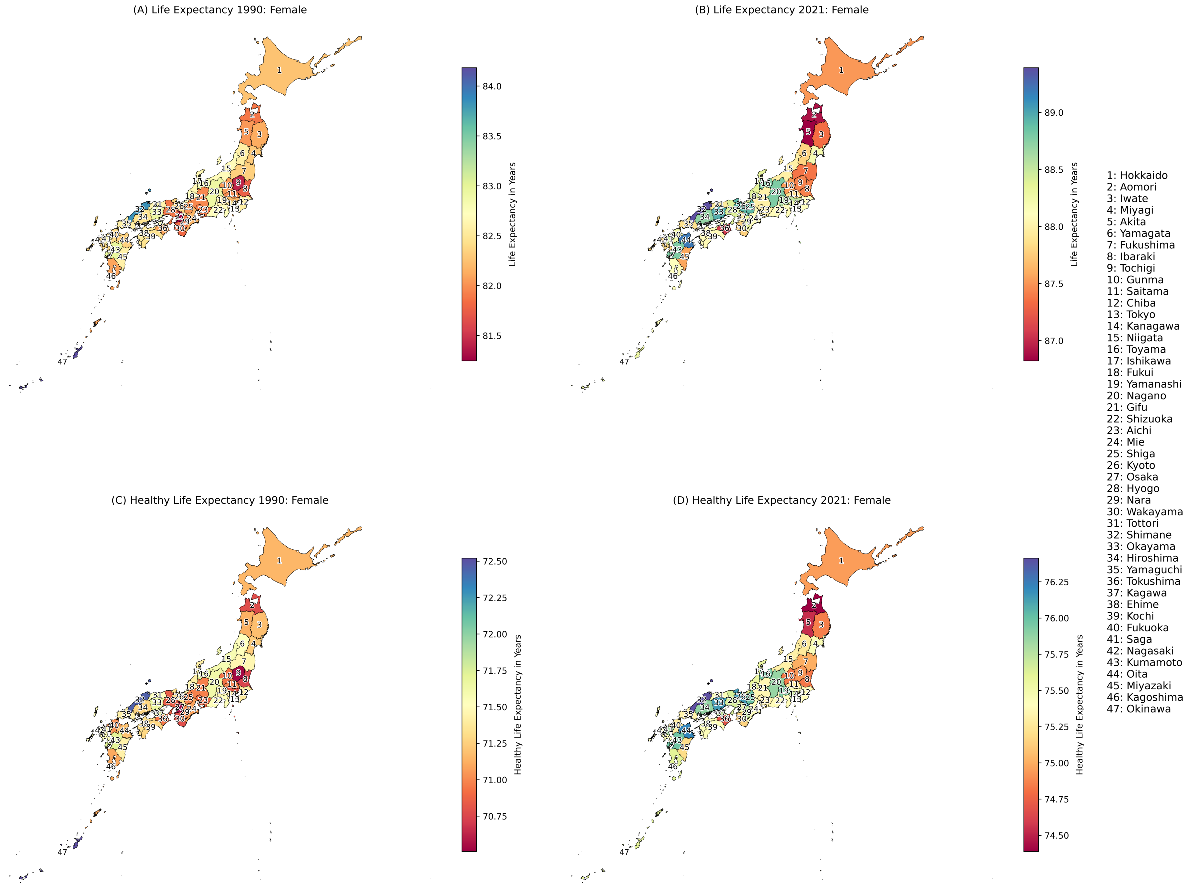

Pefectures are numbered from 1 to 47 according to their ISO codes.

Figure S7: Maps of life expectancy and healthy life expectancy across 47 prefectures in Japan in 1990 and 2021 for males

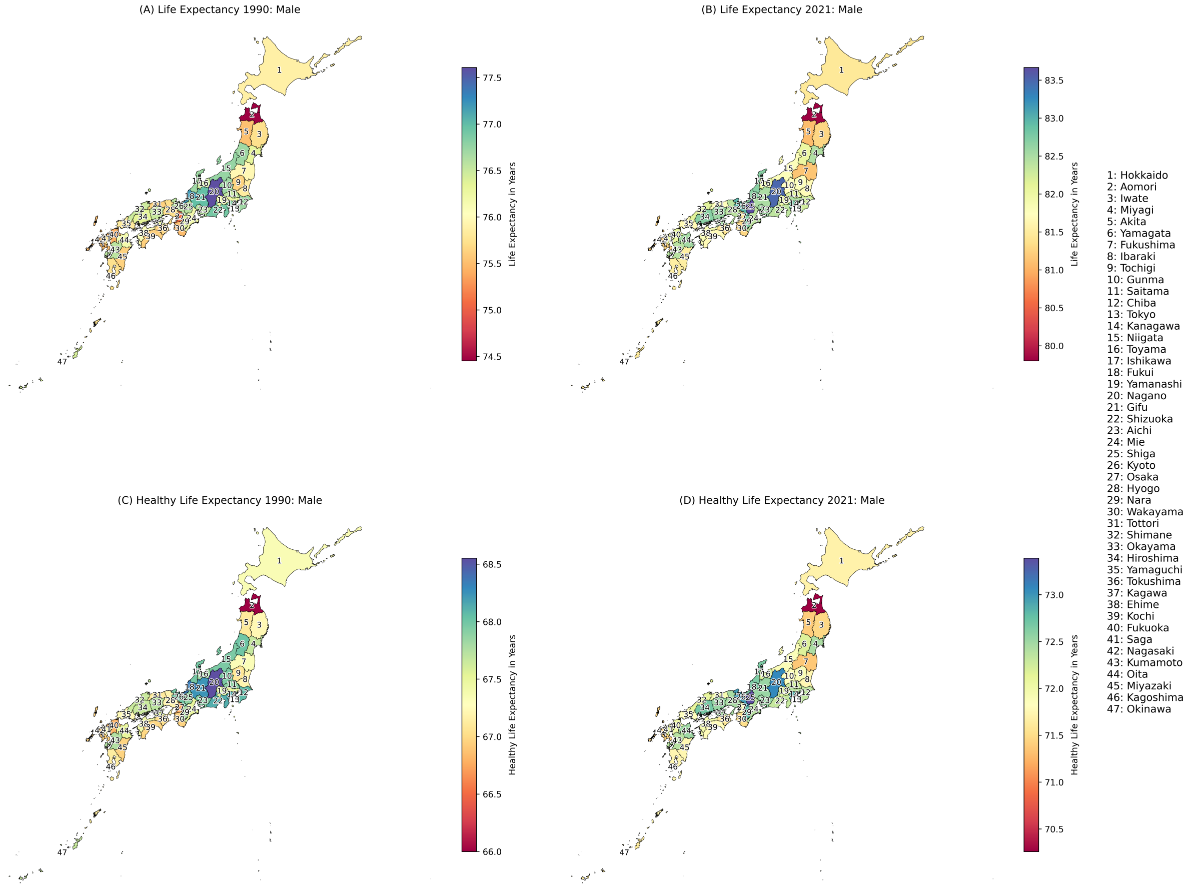

Pefectures are numbered from 1 to 47 according to their ISO codes.

Figure S8: Comparison of all-cause age-standardised mortality rates across 47 prefectures in Japan in 1990 and 2021 for both sexes

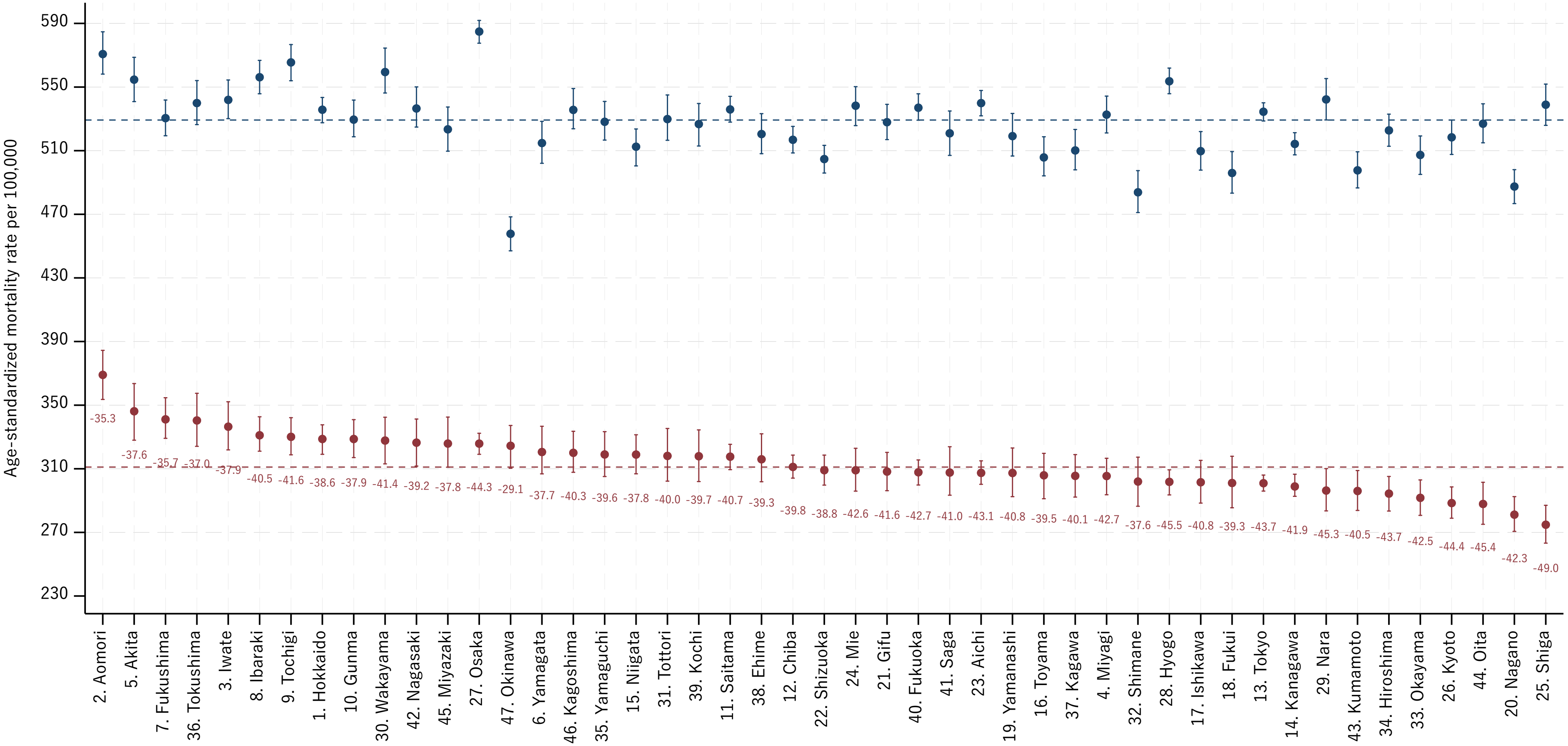

The error bars represent 95% uncertainty intervals. The red line indicates data for 2021, while the blue line represents 1990. The dotted lines show the national age-standardised mortality rate (529.3 per 100,000 population in 1990 and 311.1 in 2021). The prefectures are ordered based on their 2021 age-standardised mortality rates, from lowest to highest, and are numbered from 1 to 47 according to their ISO codes. The negative values within the figure represent the percentage dufference (%) between 1990 and 2021.

Figure S9: GBD level 3 causes of death in Japan for the years 1990, 2005, 2015, and 2021 for all ages and females, with annualised rates of change (ARC) for age-standardised mortality rates

1990

Rank	Leading causes
1	Stroke
2	Ischemic heart disease
3	Alzheimer's disease and other dementias
4	Lower respiratory infections
5	Stomach cancer
6	Colon and rectum cancer
7	Lung cancer
8	Chronic kidney disease
9	Hypertensive heart disease
10	Self-harm
11	Cirrhosis and other chronic liver diseases
12	Gallbladder and biliary tract cancer
13	Pancreatic cancer
14	Breast cancer
15	Chronic obstructive pulmonary disease
16	Diabetes mellitus
17	Non-rheumatic valvular heart disease
18	Liver cancer
19	Cardiomyopathy and myocarditis
20	Road injuries
21	Ovarian cancer
27	Parkinson's disease
29	Atrial fibrillation and flutter
32	Interstitial lung disease
34	Aortic aneurysm

2005

Rank	Leading causes	ARC (%) 1990-2005
1	Stroke	-4.8
2	Alzheimer's disease and other dementias	-0.2
3	Ischemic heart disease	-4.8
4	Lower respiratory infections	-3.0
5	Colon and rectum cancer	-0.9
6	Stomach cancer	-3.6
7	Lung cancer	-0.2
8	Chronic kidney disease	-1.0
9	Liver cancer	1.9
10	Pancreatic cancer	0.1
11	Breast cancer	1.9
12	Gallbladder and biliary tract cancer	-2.4
13	Self-harm	-0.3
14	Cirrhosis and other chronic liver diseases	-2.7
15	Chronic obstructive pulmonary disease	-4.6
16	Non-rheumatic valvular heart disease	-4.0
17	Hypertensive heart disease	-8.5
18	Aortic aneurysm	3.1
19	Parkinson's disease	0.7
20	Ovarian cancer	-0.2
21	Interstitial lung disease	2.1
23	Diabetes mellitus	-7.3
25	Atrial fibrillation and flutter	-2.1
29	Cardiomyopathy and myocarditis	-6.7
30	Road injuries	-4.3

2015

Rank	Leading causes	ARC (%) 2005-2015
1	Alzheimer's disease and other dementias	0.2
2	Stroke	-4.0
3	Ischemic heart disease	-3.3
4	Lower respiratory infections	-2.7
5	Colon and rectum cancer	-0.5
6	Lung cancer	-0.4
7	Chronic kidney disease	-1.6
8	Stomach cancer	-3.3
9	Pancreatic cancer	1.4
10	Breast cancer	0.6
11	Non-rheumatic valvular heart disease	0.6
12	Liver cancer	-4.1
13	Gallbladder and biliary tract cancer	-2.5
14	Aortic aneurysm	2.3
15	Cirrhosis and other chronic liver diseases	-2.3
16	Chronic obstructive pulmonary disease	-2.7
17	Hypertensive heart disease	-1.7
18	Self-harm	-1.9
19	Parkinson's disease	0.4
20	Interstitial lung disease	0.8
22	Atrial fibrillation and flutter	0.0
23	Ovarian cancer	-0.7
29	Diabetes mellitus	-3.2
30	Cardiomyopathy and myocarditis	-1.5
46	Road injuries	-7.0

2021

Rank	Leading causes	ARC (%) 2015-2021
1	Alzheimer's disease and other dementias	-0.4
2	Stroke	-2.3
3	Ischemic heart disease	-2.3
4	Colon and rectum cancer	-0.8
5	Lower respiratory infections	-8.8
6	Lung cancer	-1.0
7	Chronic kidney disease	-0.7
8	Pancreatic cancer	0.7
9	Stomach cancer	-2.8
10	Breast cancer	-0.2
11	Non-rheumatic valvular heart disease	-0.3
12	Aortic aneurysm	0.6
13	Gallbladder and biliary tract cancer	-2.6
14	Hypertensive heart disease	1.6
15	Liver cancer	-3.5
16	Chronic obstructive pulmonary disease	-1.3
17	Interstitial lung disease	-0.2
18	Cirrhosis and other chronic liver diseases	-2.4
19	Parkinson's disease	-0.7
20	Atrial fibrillation and flutter	0.4
24	Self-harm	-1.6
27	Ovarian cancer	-0.9
28	COVID-19	
30	Diabetes mellitus	-0.2
31	Cardiomyopathy and myocarditis	-2.1
54	Road injuries	-3.7

Legend:
Communicable, maternal, neonatal, and nutritional diseases
Non-communicable diseases
Injuries

Rankings are based on the number of deaths from each cause.

Figure S10: GBD level 3 causes of death in Japan for the years 1990, 2005, 2015, and 2021 for all ages and males, with annualised rates of change (ARC) for age-standardised mortality rates

Rank	1990 Leading causes	Rank	2005 Leading causes	ARC (%) 1990-2005	Rank	2015 Leading causes	ARC (%) 2005-2015	Rank	2021 Leading causes	ARC (%) 2015-2021
1	Stroke	1	Stroke	-3.3	1	Stroke	-3.8	1	Stroke	-1.8
2	Ischemic heart disease	2	Ischemic heart disease	-3.4	2	Lower respiratory infections	-2.7	2	Ischemic heart disease	-1.5
3	Lower respiratory infections	3	Lower respiratory infections	-1.7	3	Lung cancer	-1.1	3	Lung cancer	-1.4
4	Stomach cancer	4	Lung cancer	-0.2	4	Ischemic heart disease	-2.5	4	Lower respiratory infections	-7.9
5	Lung cancer	5	Stomach cancer	-2.8	5	Stomach cancer	-3.0	5	Alzheimer's disease and other dementias	0.0
6	Colon and rectum cancer	6	Colon and rectum cancer	-0.4	6	Alzheimer's disease and other dementias	0.1	6	Stomach cancer	-2.8
7	Cirrhosis and other chronic liver diseases	7	Liver cancer	0.5	7	Colon and rectum cancer	-0.6	7	Colon and rectum cancer	-1.0
8	Liver cancer	8	Self-harm	2.5	8	Chronic kidney disease	-1.0	8	Chronic kidney disease	0.1
9	Self-harm	9	Alzheimer's disease and other dementias	-0.1	9	Liver cancer	-4.3	9	Chronic obstructive pulmonary disease	0.2
10	Chronic obstructive pulmonary disease	10	Chronic kidney disease	-0.1	10	Chronic obstructive pulmonary disease	-2.2	10	Pancreatic cancer	0.3
11	Road injuries	11	Chronic obstructive pulmonary disease	-3.0	11	Pancreatic cancer	0.5	11	Liver cancer	-2.7
12	Alzheimer's disease and other dementias	12	Cirrhosis and other chronic liver diseases	-2.8	12	Self-harm	-2.7	12	Prostate cancer	-0.6
13	Chronic kidney disease	13	Pancreatic cancer	-0.0	13	Prostate cancer	-1.3	13	Self-harm	-2.3
14	Pancreatic cancer	14	Esophageal cancer	-0.3	14	Cirrhosis and other chronic liver diseases	-2.7	14	Interstitial lung disease	0.6
15	Esophageal cancer	15	Prostate cancer	1.6	15	Interstitial lung disease	0.9	15	Cirrhosis and other chronic liver diseases	-0.2
16	Diabetes mellitus	16	Gallbladder and biliary tract cancer	-0.9	16	Esophageal cancer	-1.6	16	Esophageal cancer	-1.5
17	Gallbladder and biliary tract cancer	17	Interstitial lung disease	3.1	17	Gallbladder and biliary tract cancer	-1.5	17	Aortic aneurysm	0.1
18	Cardiomyopathy and myocarditis	18	Aortic aneurysm	2.1	18	Aortic aneurysm	0.5	18	Gallbladder and biliary tract cancer	-1.3
19	Hypertensive heart disease	19	Road injuries	-4.6	19	Parkinson's disease	0.4	19	Parkinson's disease	-0.5
20	Prostate cancer	20	Falls	0.9	20	Non-Hodgkin lymphoma	0.2	20	Falls	0.6
23	Non-Hodgkin lymphoma	21	Non-Hodgkin lymphoma	-0.1	21	Falls	-1.1	21	COVID-19	
26	Falls	22	Parkinson's disease	0.2	31	Cardiomyopathy and myocarditis	-1.9	22	Non-Hodgkin lymphoma	-0.5
27	Aortic aneurysm	26	Diabetes mellitus	-5.2	32	Diabetes mellitus	-2.6	31	Diabetes mellitus	1.2
28	Interstitial lung disease	27	Cardiomyopathy and myocarditis	-4.8	33	Road injuries	-6.5	34	Hypertensive heart disease	4.2
29	Parkinson's disease	35	Hypertensive heart disease	-8.2	36	Hypertensive heart disease	-2.1	36	Cardiomyopathy and myocarditis	-1.9
								40	Road injuries	-4.0

Legend:
Communicable, maternal, neonatal, and nutritional diseases
Non-communicable diseases
Injuries

Rankings are based on the number of deaths from each cause.

Figure S11: Comparison of COVID-19 age-standardised mortality rates across 47 prefectures in Japan in (A) 2020 and (B) 2021 for both sexes

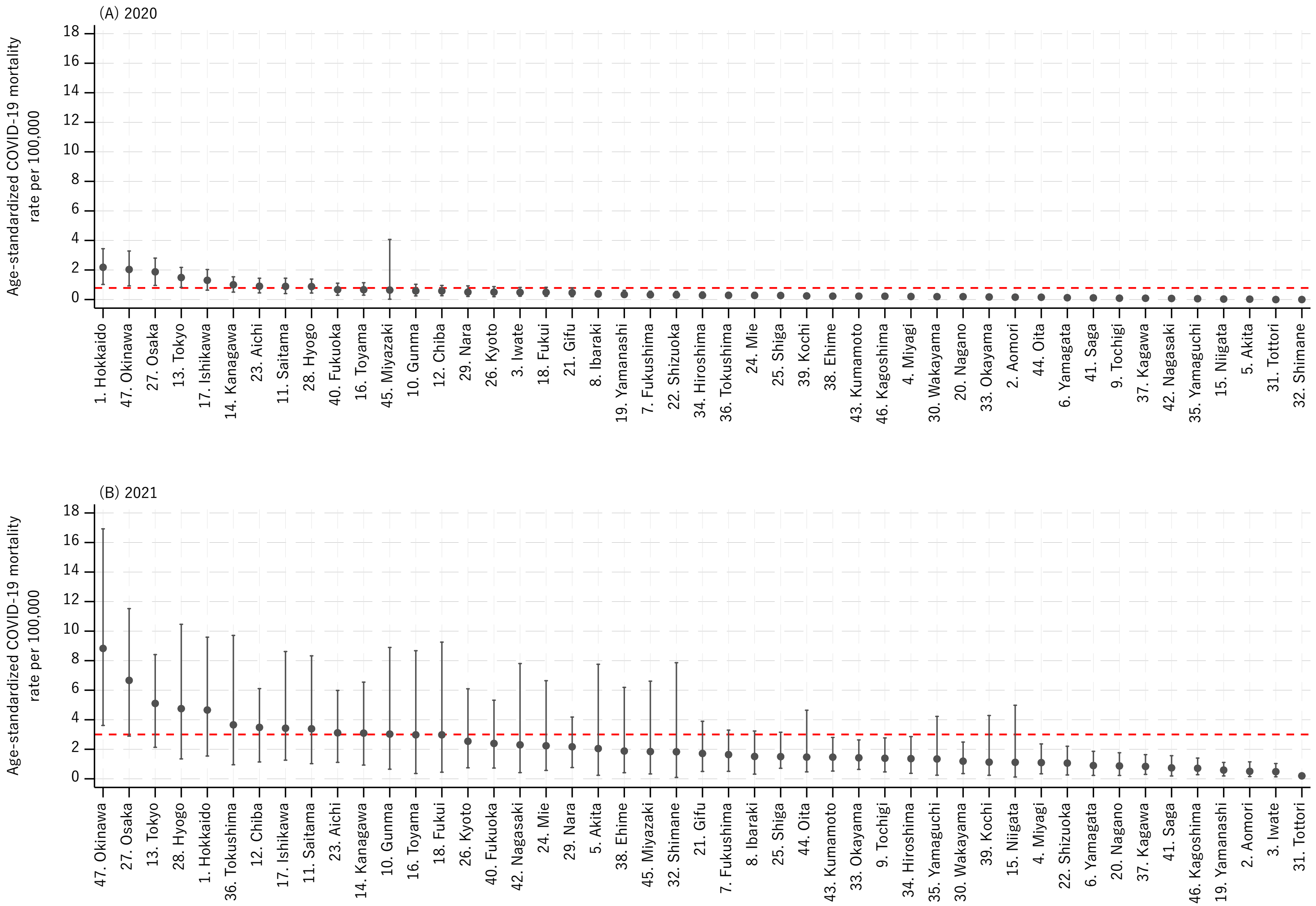

The error bars represent 95% uncertainty intervals; the red dotted lines indicate the national age-standardised mortality rate (0.8 per 100,000 population in 2020 and 3.0 in 2021). Prefectures are numbered from 1 to 47 according to their ISO codes.

Figure S12: GBD level 3 causes of disability-adjusted life years (DALYs) in Japan for the years 1990, 2005, 2015, and 2021 for all ages and both sexes, with annualised rates of change (ARC) for age-standardised DALY rate

Rank	1990 Leading causes	Rank	2005 Leading causes	ARC (%) 1990-2005	Rank	2015 Leading causes	ARC (%) 2005-2015	Rank	2021 Leading causes	ARC (%) 2015-2021
1	Stroke	1	Stroke	-3.1	1	Stroke	-3.0	1	Stroke	-1.3
2	Low back pain	2	Low back pain	-0.3	2	Low back pain	-0.0	2	Alzheimer's disease and other dementias	-0.5
3	Ischemic heart disease	3	Ischemic heart disease	-3.0	3	Alzheimer's disease and other dementias	0.1	3	Low back pain	-0.7
4	Stomach cancer	4	Alzheimer's disease and other dementias	0.0	4	Ischemic heart disease	-2.6	4	Ischemic heart disease	-1.6
5	Lower respiratory infections	5	Lung cancer	-0.4	5	Lung cancer	-1.1	5	Lung cancer	-1.7
6	Road injuries	6	Self-harm	2.4	6	Other musculoskeletal disorders	0.6	6	Other musculoskeletal disorders	0.3
7	Other musculoskeletal disorders	7	Other musculoskeletal disorders	0.6	7	Lower respiratory infections	-2.5	7	Diabetes mellitus	2.2
8	Lung cancer	8	Lower respiratory infections	-2.4	8	Diabetes mellitus	0.1	8	Colon and rectum cancer	-1.1
9	Self-harm	9	Stomach cancer	-3.4	9	Colon and rectum cancer	-0.5	9	Age-related and other hearing loss	-0.1
10	Colon and rectum cancer	10	Diabetes mellitus	0.4	10	Osteoarthritis	2.0	10	Lower respiratory infections	-8.2
11	Diabetes mellitus	11	Colon and rectum cancer	-0.7	11	Age-related and other hearing loss	0.0	11	Osteoarthritis	-2.9
12	Alzheimer's disease and other dementias	12	Falls	0.1	12	Stomach cancer	-3.5	12	Stomach cancer	-3.2
13	Headache disorders	13	Age-related and other hearing loss	0.0	13	Self-harm	-2.1	13	Chronic kidney disease	-0.8
14	Falls	14	Liver cancer	-0.1	14	Chronic kidney disease	-0.9	14	Falls	-1.0
15	Cirrhosis and other chronic liver diseases	15	Osteoarthritis	0.3	15	Falls	-2.0	15	Self-harm	-1.7
16	Asthma	16	Chronic kidney disease	-0.4	16	Headache disorders	0.3	16	Pancreatic cancer	0.3
17	Age-related and other hearing loss	17	Headache disorders	-0.0	17	Pancreatic cancer	0.6	17	Chronic obstructive pulmonary disease	-1.1
18	Liver cancer	18	Depressive disorders	0.8	18	Chronic obstructive pulmonary disease	-1.4	18	Depressive disorders	3.0
19	Depressive disorders	19	Cirrhosis and other chronic liver diseases	-2.6	19	Depressive disorders	-0.7	19	Headache disorders	-0.0
20	Osteoarthritis	20	Road injuries	-4.5	20	Liver cancer	-5.1	20	Liver cancer	-3.5
22	Chronic kidney disease	21	Chronic obstructive pulmonary disease	-2.4	21	Cirrhosis and other chronic liver diseases	-2.9	23	Cirrhosis and other chronic liver diseases	-0.8
23	Chronic obstructive pulmonary disease	22	Pancreatic cancer	0.1	30	Road injuries	-6.2	39	Road injuries	-3.3
28	Pancreatic cancer	30	Asthma	-5.1	47	Asthma	-3.6	41	COVID-19	
								51	Asthma	-0.4

Legend:
Communicable, maternal, neonatal, and nutritional diseases
Non-communicable diseases
Injuries

Rankings are based on the number of DALYs from each cause.

Figure S13: GBD level 3 causes of disability-adjusted life years (DALYs) in Japan for the years 1990, 2005, 2015, and 2021 for all ages and females, with annualised rates of change (ARC) for age-standardised DALY rate

1990

Rank	Leading causes
1	Stroke
2	Low back pain
3	Ischemic heart disease
4	Other musculoskeletal disorders
5	Alzheimer's disease and other dementias
6	Stomach cancer
7	Headache disorders
8	Gynecological diseases
9	Lower respiratory infections
10	Colon and rectum cancer
11	Asthma
12	Osteoarthritis
13	Diabetes mellitus
14	Self-harm
15	Depressive disorders
16	Age-related and other hearing loss
17	Falls
18	Anxiety disorders
19	Road injuries
20	Lung cancer
21	Breast cancer
23	Chronic kidney disease
30	Pancreatic cancer

2005

Rank	Leading causes	ARC (%) 1990-2005
1	Low back pain	-0.4
2	Stroke	-3.8
3	Alzheimer's disease and other dementias	0.0
4	Other musculoskeletal disorders	0.7
5	Ischemic heart disease	-4.3
6	Osteoarthritis	0.5
7	Diabetes mellitus	0.1
8	Age-related and other hearing loss	0.0
9	Lower respiratory infections	-3.1
10	Colon and rectum cancer	-0.9
11	Headache disorders	-0.1
12	Falls	0.4
13	Stomach cancer	-4.0
14	Depressive disorders	0.9
15	Breast cancer	1.8
16	Lung cancer	-0.5
17	Self-harm	0.9
18	Chronic kidney disease	-0.9
19	Gynecological diseases	-1.0
20	Anxiety disorders	-0.1
23	Pancreatic cancer	0.1
28	Asthma	-4.8
29	Road injuries	-4.1

2015

Rank	Leading causes	ARC (%) 2005-2015
1	Alzheimer's disease and other dementias	0.2
2	Low back pain	-0.2
3	Stroke	-3.3
4	Other musculoskeletal disorders	0.2
5	Osteoarthritis	3.0
6	Ischemic heart disease	-3.4
7	Age-related and other hearing loss	-0.0
8	Diabetes mellitus	0.3
9	Colon and rectum cancer	-0.7
10	Lower respiratory infections	-3.0
11	Headache disorders	0.2
12	Breast cancer	0.2
13	Lung cancer	-0.8
14	Falls	-1.8
15	Chronic kidney disease	-1.2
16	Depressive disorders	-0.9
17	Stomach cancer	-3.8
18	Pancreatic cancer	1.0
19	Gynecological diseases	0.1
20	Self-harm	-1.8
21	Anxiety disorders	-1.3
35	Asthma	-1.8
44	Road injuries	-6.3

2021

Rank	Leading causes	ARC (%) 2015-2021
1	Alzheimer's disease and other dementias	-0.4
2	Low back pain	-0.0
3	Stroke	-1.4
4	Other musculoskeletal disorders	0.1
5	Ischemic heart disease	-2.1
6	Diabetes mellitus	2.3
7	Osteoarthritis	-4.3
8	Age-related and other hearing loss	-0.1
9	Colon and rectum cancer	-1.0
10	Breast cancer	-0.5
11	Chronic kidney disease	-1.3
12	Falls	-0.9
13	Lung cancer	-1.6
14	Headache disorders	-0.0
15	Depressive disorders	3.3
16	Pancreatic cancer	0.6
17	Lower respiratory infections	-9.2
18	Stomach cancer	-3.3
19	Anxiety disorders	5.5
20	Gynecological diseases	0.3
22	Self-harm	-0.7
38	Asthma	-1.7
46	COVID-19	
53	Road injuries	-2.8

Legend:
Communicable, maternal, neonatal, and nutritional diseases
Non-communicable diseases
Injuries

Rankings are based on the number of DALYs from each cause.

Figure S14: GBD level 3 causes of disability-adjusted life years (DALYs) in Japan for the years 1990, 2005, 2015, and 2021 for all ages and males, with annualised rates of change (ARC) for age-standardised DALY rate

Rank	1990 Leading causes
1	Stroke
2	Ischemic heart disease
3	Stomach cancer
4	Low back pain
5	Road injuries
6	Lung cancer
7	Lower respiratory infections
8	Self-harm
9	Cirrhosis and other chronic liver diseases
10	Colon and rectum cancer
11	Liver cancer
12	Diabetes mellitus
13	Falls
14	Other musculoskeletal disorders
15	Asthma
16	Chronic obstructive pulmonary disease
17	Age-related and other hearing loss
18	Chronic kidney disease
19	Congenital birth defects
20	Autism spectrum disorders
22	Depressive disorders
23	Alzheimer's disease and other dementias
24	Pancreatic cancer
29	Osteoarthritis
31	Oral disorders

Rank	2005 Leading causes	ARC (%) 1990-2005
1	Stroke	-2.6
2	Ischemic heart disease	-2.5
3	Lung cancer	-0.5
4	Self-harm	3.0
5	Low back pain	-0.1
6	Stomach cancer	-3.2
7	Lower respiratory infections	-2.0
8	Colon and rectum cancer	-0.5
9	Diabetes mellitus	0.5
10	Liver cancer	-0.5
11	Falls	-0.0
12	Other musculoskeletal disorders	0.4
13	Age-related and other hearing loss	-0.0
14	Cirrhosis and other chronic liver diseases	-2.7
15	Alzheimer's disease and other dementias	0.0
16	Road injuries	-4.6
17	Chronic kidney disease	-0.0
18	Chronic obstructive pulmonary disease	-2.1
19	Pancreatic cancer	-0.1
20	Oral disorders	0.9
21	Osteoarthritis	0.1
22	Autism spectrum disorders	0.3
23	Depressive disorders	0.7
33	Congenital birth defects	-2.7
35	Asthma	-5.5

Rank	2015 Leading causes	ARC (%) 2005-2015
1	Stroke	-3.0
2	Ischemic heart disease	-2.4
3	Lung cancer	-1.3
4	Low back pain	0.2
5	Lower respiratory infections	-2.6
6	Self-harm	-2.2
7	Stomach cancer	-3.4
8	Colon and rectum cancer	-0.5
9	Diabetes mellitus	-0.0
10	Alzheimer's disease and other dementias	0.2
11	Other musculoskeletal disorders	1.3
12	Age-related and other hearing loss	0.1
13	Chronic kidney disease	-0.7
14	Falls	-2.1
15	Chronic obstructive pulmonary disease	-1.6
16	Liver cancer	-5.2
17	Pancreatic cancer	0.2
18	Cirrhosis and other chronic liver diseases	-3.0
19	Osteoarthritis	0.3
20	Autism spectrum disorders	0.5
22	Depressive disorders	-0.4
26	Road injuries	-6.2
28	Oral disorders	-3.8
40	Congenital birth defects	-1.7
54	Asthma	-5.7

Rank	2021 Leading causes	ARC (%) 2015-2021
1	Stroke	-1.3
2	Ischemic heart disease	-1.5
3	Lung cancer	-1.9
4	Low back pain	-1.6
5	Diabetes mellitus	2.0
6	Alzheimer's disease and other dementias	-0.2
7	Colon and rectum cancer	-1.2
8	Lower respiratory infections	-8.0
9	Stomach cancer	-3.3
10	Self-harm	-2.1
11	Other musculoskeletal disorders	0.5
12	Age-related and other hearing loss	-0.1
13	Chronic kidney disease	-0.5
14	Chronic obstructive pulmonary disease	-1.1
15	Falls	-1.1
16	Pancreatic cancer	0.1
17	Liver cancer	-3.3
18	Osteoarthritis	0.0
19	Cirrhosis and other chronic liver diseases	-0.4
20	Depressive disorders	2.7
23	Autism spectrum disorders	-0.3
24	Oral disorders	2.9
30	Road injuries	-3.5
37	COVID-19	
47	Congenital birth defects	-1.7
57	Asthma	1.3

Legend:
Communicable, maternal, neonatal, and nutritional diseases
Non-communicable diseases
Injuries

Rankings are based on the number of DALYs from each cause.

Figure S15: GBD level 3 risk factors for (A) mortality and (B) DALYs in Japan for the years 1990, 2005, 2015, and 2021 for all ages and both sexes, with annualised rates of change (ARC) for age-standardised rates

(A) Mortality

Rank	1990 Leading risks	2005 Leading risks	ARC 1990-2005	2015 Leading risks	ARC 2005-2015	2021 Leading risks	ARC 2015-2021
1	High systolic blood pressure	High systolic blood pressure	-4.6	High systolic blood pressure	-3.5	High systolic blood pressure	-1.0
2	Tobacco	Tobacco	-2.8	Tobacco	-3.2	Tobacco	-2.1
3	Dietary risks	Dietary risks	-3.5	Dietary risks	-2.4	High fasting plasma glucose	-0.6
4	High fasting plasma glucose	High fasting plasma glucose	-2.6	High fasting plasma glucose	-1.9	Dietary risks	-1.3
5	High LDL cholesterol	Kidney dysfunction	-2.7	Kidney dysfunction	-2.0	Kidney dysfunction	-0.8
6	Kidney dysfunction	High LDL cholesterol	-4.0	High LDL cholesterol	-3.3	High LDL cholesterol	-2.0
7	Air pollution	Air pollution	-3.7	Air pollution	-1.9	Air pollution	-2.6
8	Alcohol use	Non-optimal temperature	-3.6	Non-optimal temperature	-3.5	High body-mass index	0.7
9	Non-optimal temperature	Alcohol use	-1.9	High body-mass index	-0.7	Non-optimal temperature	-3.4
10	High body-mass index	High body-mass index	-2.3	Alcohol use	-3.2	Alcohol use	-1.0

(B) DALYs

Rank	1990 Leading risks	2005 Leading risks	ARC 1990-2005	2015 Leading risks	ARC 2005-2015	2021 Leading risks	ARC 2015-2021
1	Tobacco	Tobacco	-2.1	Tobacco	-2.9	High fasting plasma glucose	0.8
2	High systolic blood pressure	High systolic blood pressure	-3.9	High fasting plasma glucose	-0.8	Tobacco	-2.1
3	Dietary risks	High fasting plasma glucose	-0.7	High systolic blood pressure	-3.0	High systolic blood pressure	-0.7
4	High fasting plasma glucose	Dietary risks	-2.3	Dietary risks	-1.8	Dietary risks	-0.8
5	Occupational risks	Alcohol use	-1.3	High body-mass index	0.2	High body-mass index	1.4
6	Alcohol use	Kidney dysfunction	-2.0	Kidney dysfunction	-1.5	Kidney dysfunction	-1.0
7	High LDL cholesterol	High body-mass index	-0.3	Occupational risks	-1.1	Occupational risks	-0.5
8	Kidney dysfunction	Occupational risks	-2.3	Alcohol use	-2.8	Alcohol use	-0.9
9	High body-mass index	High LDL cholesterol	-2.9	Air pollution	-1.4	Air pollution	-1.6
10	Air pollution	Air pollution	-2.6	High LDL cholesterol	-2.8	High LDL cholesterol	-1.7

Legend:
Metabolic risks
Environmental/occupational risks
Behavioral risks

Rankings are based on the number of deaths and DALYs attributable to each risk factor, respectively.

Supplementary Tables

Table S1: GATHER checklist

Item#	Checklist item	Reporting location
**Objectives and funding**		
1	Define the indicator(s), populations (including age, sex, and geographic entities), and time period(s) for which estimates were made.	Main text methods
2	List the funding sources for the work.	Main text (acknowledgement section); primary funder given in the "funding" section of the summary
**Data Inputs**		
For all data inputs from multiple sources that are synthesized as part of the study:		
3	Describe how the data were identified and how the data were accessed.	Main text methods, with references provided for access to full details
4	Specify the inclusion and exclusion criteria. Identify all ad-hoc exclusions	References provided in the main text methods for access to full details
5	Provide information on all included data sources and their main characteristics. For each data source used, report reference information or contact name/institution, population represented, data collection method, year(s) of data collection, sex and age range, diagnostic criteria or measurement method, and sample size, as relevant.	Overview in main text methods; citations for all references available at https://ghdx.healthdata.org/gbd-2021/sources
6	Identify and describe any categories of input data that have potentially important biases (e.g., based on characteristics listed in item 5).	Main text limitations section
For data inputs that contribute to the analysis but were not synthesized as part of the study:		
7	Describe and give sources for any other data inputs.	NA
For all data inputs:		
8	Provide all data inputs in a file format from which data can be efficiently extracted (e.g., a spreadsheet rather than a PDF), including all relevant meta-data listed in item 5. For any data inputs that cannot be shared because of ethical or legal reasons, such as third-party ownership, provide a contact name or the name of the institution that retains the right to the data.	http://ghdx.healthdata.org/gbd-2021/sources<br>Availability of data is dependent on data use agreements; contact information provided when not directly available
**Data analysis**		
9	Provide a conceptual overview of the data analysis method. A diagram may be helpful.	Main text methods, with references provided for access to full details

10	Provide a detailed description of all steps of the analysis, including mathematical formulae. This description should cover, as relevant, data cleaning, data pre-processing, data adjustments and weighting of data sources, and mathematical or statistical model(s).	Main text methods, with references provided for access to full details
11	Describe how candidate models were evaluated and how the final model(s) were selected.	References provided in the main text methods for access to full details
12	Provide the results of an evaluation of model performance, if done, as well as the results of any relevant sensitivity analysis.	NA
13	Describe methods for calculating uncertainty of the estimates. State which sources of uncertainty were, and were not, accounted for in the uncertainty analysis.	Main text methods
14	State how analytic or statistical source code used to generate estimates can be accessed.	https://ghdx.healthdata.org/gbd-2021/code
**Results and Discussion**		
15	Provide published estimates in a file format from which data can be efficiently extracted.	All estimates are available for download at https://vizhub.healthdata.org/gbd-results/ and https://vizhub.healthdata.org/gbd-compare/
16	Report a quantitative measure of the uncertainty of the estimates (e.g. uncertainty intervals).	UIs provided for all estimates throughout the main text (summary, results, and discussion sections)
17	Interpret results in light of existing evidence. If updating a previous set of estimates, describe the reasons for changes in estimates.	Main text discussion
18	Discuss limitations of the estimates. Include a discussion of any modelling assumptions or data limitations that affect interpretation of the estimates.	Main text limitations section

Table S2: Life expectancy and healthy life expectancy at birth (years) in Japan and 47 prefectures, 1990 and 2021, for both sexes

	Life expectancy (95% UI)						Healthy life expectancy (95% UI)					
Prefecture	1990	Rank	2021	Rank	Increase	Rank	1990	Rank	2021	Rank	Increase	Rank
**Japan**	**79.4 (79.3–79.4)**		**85.2 (85.1–85.2)**		**5.8**		**69.4 (66.6–72.0)**		**73.8 (70.5–76.7)**		**4.4**	
Hokkaido	79.1 (78.9–79.3)	37	84.6 (84.3–84.9)	39	5.5	34	69.3 (66.4–71.7)	35	73.4 (70.2–76.1)	39	4.1	35
Aomori	78.3 (78.0–78.6)	46	83.4 (82.9–83.9)	47	5.1	42	68.5 (65.6–70.9)	47	72.4 (69.2–75.1)	47	3.9	40
Iwate	79.0 (78.8–79.3)	39	84.3 (83.8–84.8)	43	5.3	39	69.3 (66.4–71.6)	34	73.2 (70.0–75.9)	43	3.9	41
Miyagi	79.4 (79.2–79.6)	23	85.4 (85.0–85.8)	17	6.0	10	69.5 (66.7–72.0)	20	74.0 (70.8–76.8)	18	4.4	18
Akita	78.9 (78.6–79.2)	41	84.0 (83.5–84.5)	46	5.1	43	69.2 (66.4–71.6)	39	73.0 (69.8–75.8)	46	3.8	43
Yamagata	79.8 (79.5–80.1)	11	85.0 (84.5–85.4)	27	5.2	41	69.9 (67.0–72.3)	7	73.8 (70.5–76.5)	25	3.9	42
Fukushima	79.3 (79.0–79.5)	33	84.2 (83.8–84.6)	45	5.0	46	69.5 (66.7–72.0)	24	73.2 (70.0–76.0)	44	3.7	46
Ibaraki	78.8 (78.6–79.1)	43	84.5 (84.1–84.8)	42	5.6	28	69.0 (66.1–71.4)	42	73.2 (70.0–76.1)	42	4.2	30
Tochigi	78.6 (78.4–78.8)	45	84.5 (84.1–84.9)	41	5.9	14	68.9 (66.1–71.3)	45	73.4 (70.2–76.1)	38	4.5	13
Gunma	79.5 (79.2–79.7)	19	84.6 (84.2–84.9)	40	5.1	44	69.5 (66.6–72.0)	21	73.3 (70.0–76.1)	41	3.8	44
Saitama	79.3 (79.1–79.5)	27	84.9 (84.7–85.2)	28	5.6	27	69.5 (66.7–72.0)	22	73.7 (70.5–76.5)	26	4.2	29
Chiba	79.7 (79.5–79.9)	12	85.1 (84.9–85.4)	25	5.4	36	69.8 (66.8–72.3)	12	73.8 (70.6–76.6)	24	4.0	38
Tokyo	79.3 (79.2–79.4)	28	85.5 (85.4–85.7)	13	6.2	7	69.2 (66.3–71.7)	38	73.9 (70.5–76.9)	20	4.7	9
Kanagawa	79.7 (79.5–79.8)	14	85.5 (85.3–85.8)	12	5.9	16	69.8 (66.9–72.3)	10	74.3 (70.9–77.0)	9	4.4	15
Niigata	79.9 (79.6–80.1)	7	84.9 (84.5–85.3)	31	5.0	45	69.9 (67.0–72.3)	6	73.6 (70.3–76.4)	34	3.7	45
Toyama	79.8 (79.5–80.1)	10	85.5 (85.0–86.0)	14	5.7	26	69.8 (66.9–72.3)	9	74.1 (71.0–77.0)	12	4.3	24
Ishikawa	79.8 (79.6–80.1)	8	85.6 (85.1–86.0)	10	5.7	22	69.8 (66.9–72.3)	11	74.1 (70.9–76.9)	13	4.3	23
Fukui	80.1 (79.8–80.4)	4	85.6 (85.0–86.1)	11	5.5	35	70.0 (67.1–72.6)	4	74.2 (70.9–77.0)	11	4.1	33
Yamanashi	79.5 (79.2–79.8)	17	85.3 (84.8–85.7)	19	5.7	20	69.6 (66.7–72.0)	17	74.0 (70.8–76.8)	17	4.4	16
Nagano	80.4 (80.1–80.6)	2	86.1 (85.7–86.5)	2	5.8	18	70.2 (67.3–72.7)	2	74.5 (71.2–77.3)	3	4.3	25
Gifu	79.6 (79.3–79.8)	16	85.2 (84.9–85.6)	22	5.7	25	69.7 (66.7–72.2)	16	73.9 (70.7–76.8)	19	4.3	26
Shizuoka	79.9 (79.7–80.1)	6	85.2 (84.9–85.5)	24	5.3	38	69.9 (67.0–72.4)	5	73.8 (70.6–76.7)	23	3.9	39
Aichi	79.3 (79.1–79.5)	26	85.2 (85.0–85.5)	20	5.9	13	69.5 (66.6–72.0)	26	73.9 (70.6–76.8)	21	4.4	17
Mie	79.3 (79.1–79.6)	29	85.2 (84.8–85.7)	23	5.9	12	69.4 (66.5–71.9)	31	73.9 (70.7–76.8)	22	4.5	12
Shiga	79.4 (79.2–79.7)	21	86.3 (85.9–86.7)	1	6.8	1	69.5 (66.6–72.0)	18	74.7 (71.3–77.6)	1	5.2	1
Kyoto	79.6 (79.4–79.9)	15	86.0 (85.6–86.4)	3	6.4	5	69.8 (66.9–72.2)	13	74.6 (71.3–77.4)	2	4.8	5
Osaka	78.2 (78.1–78.4)	47	84.7 (84.5–84.9)	35	6.5	4	68.7 (65.9–71.1)	46	73.7 (70.5–76.5)	30	5.0	2
Hyogo	78.9 (78.7–79.1)	42	85.4 (85.2–85.7)	15	6.5	3	69.1 (66.3–71.5)	40	74.1 (70.8–77.0)	15	5.0	3
Nara	79.3 (79.0–79.5)	32	85.6 (85.1–86.1)	9	6.3	6	69.5 (66.6–72.0)	25	74.3 (71.0–77.0)	7	4.8	6

Wakayama	78.8 (78.5–79.1)	44	84.6 (84.1–85.1)	38	5.8	17	69.0 (66.1–71.4)	44	73.4 (70.1–76.2)	40	4.4	20
Tottori	79.3 (78.9–79.6)	30	84.9 (84.3–85.4)	30	5.6	29	69.4 (66.6–71.9)	29	73.7 (70.4–76.6)	27	4.3	27
Shimane	80.3 (79.9–80.6)	3	85.8 (85.2–86.3)	6	5.5	32	70.1 (67.2–72.6)	3	74.3 (70.9–77.2)	8	4.1	34
Okayama	79.8 (79.6–80.1)	9	85.8 (85.4–86.2)	5	5.9	11	69.8 (66.8–72.3)	14	74.3 (71.1–77.2)	5	4.6	10
Hiroshima	79.5 (79.3–79.7)	18	85.7 (85.3–86.1)	7	6.2	8	69.5 (66.7–72.1)	19	74.3 (71.0–77.2)	6	4.8	7
Yamaguchi	79.3 (79.1–79.6)	25	84.8 (84.4–85.3)	33	5.5	31	69.4 (66.5–71.9)	30	73.6 (70.3–76.4)	32	4.2	31
Tokushima	79.0 (78.7–79.3)	40	84.3 (83.8–84.8)	44	5.3	40	69.1 (66.2–71.5)	41	73.2 (69.8–76.0)	45	4.1	37
Kagawa	79.7 (79.4–80.0)	13	85.4 (85.0–85.9)	16	5.7	19	69.7 (66.8–72.3)	15	74.1 (70.7–77.0)	14	4.5	14
Ehime	79.5 (79.2–79.7)	20	85.0 (84.5–85.5)	26	5.5	30	69.5 (66.5–72.0)	27	73.7 (70.5–76.5)	28	4.3	28
Kochi	79.2 (78.9–79.5)	34	84.9 (84.4–85.4)	29	5.7	23	69.2 (66.3–71.7)	37	73.6 (70.4–76.4)	33	4.4	21
Fukuoka	79.1 (78.9–79.2)	38	85.2 (85.0–85.5)	21	6.2	9	69.0 (66.1–71.5)	43	73.7 (70.3–76.5)	29	4.7	8
Saga	79.4 (79.1–79.7)	24	85.3 (84.8–85.8)	18	5.9	15	69.5 (66.6–72.1)	23	74.0 (70.7–76.9)	16	4.6	11
Nagasaki	79.1 (78.8–79.4)	35	84.6 (84.1–85.1)	37	5.5	33	69.4 (66.5–71.9)	32	73.5 (70.3–76.3)	37	4.1	36
Kumamoto	80.0 (79.7–80.2)	5	85.7 (85.2–86.1)	8	5.7	24	69.9 (66.9–72.4)	8	74.2 (70.9–77.2)	10	4.4	22
Oita	79.4 (79.1–79.7)	22	86.0 (85.5–86.4)	4	6.5	2	69.4 (66.6–71.9)	28	74.4 (71.1–77.4)	4	5.0	4
Miyazaki	79.3 (79.0–79.6)	31	84.6 (84.1–85.1)	36	5.4	37	69.3 (66.4–71.8)	33	73.5 (70.3–76.3)	36	4.2	32
Kagoshima	79.1 (78.8–79.4)	36	84.8 (84.4–85.3)	32	5.7	21	69.3 (66.4–71.8)	36	73.7 (70.5–76.6)	31	4.4	19
Okinawa	80.6 (80.3–80.8)	1	84.8 (84.3–85.3)	34	4.2	47	70.3 (67.3–73.0)	1	73.6 (70.3–76.4)	35	3.3	47

UI: uncertainty interval. Prefectures are ordered according to their ISO codes. The rankings are assigned among the 47 prefectures, with values allocated in descending order from the highest to the lowest.

Table S3: Life expectancy and healthy life expectancy at birth (years) in Japan and 47 prefectures, 1990 and 2021, for females

	Life expectancy (95% UI)						Healthy life expectancy (95% UI)					
Prefecture	1990	Rank	2021	Rank	Increase	Rank	1990	Rank	2021	Rank	Increase	Rank
**Japan**	**82.3 (82.2–82.3)**		**88.1 (88.0–88.2)**		**5.8**		**71.2 (67.9–74.0)**		**75.4 (71.6–78.7)**		**4.3**	
Hokkaido	82.2 (82.0–82.5)	29	87.5 (87.1–87.9)	40	5.3	39	71.2 (67.8–74.0)	29	74.9 (71.4–78.1)	40	3.8	38
Aomori	81.9 (81.5–82.2)	43	86.9 (86.3–87.5)	46	5.0	43	70.8 (67.6–73.6)	44	74.4 (70.7–77.4)	47	3.6	44
Iwate	82.1 (81.8–82.5)	32	87.3 (86.8–87.9)	44	5.2	41	71.2 (67.9–73.9)	28	74.9 (71.2–78.0)	42	3.7	43
Miyagi	82.3 (82.0–82.6)	28	88.2 (87.7–88.7)	21	5.9	17	71.2 (68.0–74.0)	26	75.4 (71.6–78.6)	22	4.2	24
Akita	82.0 (81.7–82.3)	36	86.8 (86.2–87.4)	47	4.8	46	71.1 (67.9–73.8)	30	74.5 (70.9–77.7)	46	3.4	46
Yamagata	82.6 (82.2–82.9)	16	87.8 (87.2–88.4)	36	5.2	40	71.5 (68.2–74.4)	11	75.3 (71.6–78.3)	36	3.7	41
Fukushima	82.3 (82.0–82.7)	27	87.4 (86.8–87.9)	43	5.0	42	71.5 (68.2–74.2)	14	75.0 (71.4–78.1)	39	3.5	45
Ibaraki	81.8 (81.4–82.0)	45	87.4 (86.9–87.9)	42	5.7	27	70.7 (67.4–73.6)	45	74.8 (71.2–78.0)	44	4.1	30
Tochigi	81.4 (81.1–81.7)	46	87.4 (86.9–87.9)	41	6.0	14	70.5 (67.3–73.4)	46	74.9 (71.3–78.1)	41	4.4	13
Gunma	82.1 (81.7–82.4)	35	87.5 (87.0–88.0)	39	5.5	32	70.9 (67.7–73.7)	39	74.8 (71.0–78.0)	43	3.9	36
Saitama	82.0 (81.7–82.2)	38	87.8 (87.5–88.2)	34	5.9	20	71.0 (67.7–73.8)	35	75.3 (71.6–78.4)	35	4.3	20
Chiba	82.6 (82.3–82.8)	18	88.1 (87.8–88.5)	22	5.6	29	71.4 (68.0–74.2)	20	75.4 (71.6–78.6)	29	4.0	31
Tokyo	82.2 (82.0–82.4)	30	88.5 (88.3–88.8)	11	6.3	7	70.8 (67.5–73.7)	42	75.4 (71.5–78.8)	23	4.6	7
Kanagawa	82.5 (82.3–82.7)	20	88.3 (88.1–88.6)	15	5.9	19	71.4 (68.0–74.2)	21	75.7 (71.9–78.8)	14	4.3	18
Niigata	82.7 (82.4–83.0)	10	88.0 (87.4–88.5)	30	5.3	38	71.6 (68.3–74.3)	8	75.3 (71.4–78.5)	32	3.7	42
Toyama	82.9 (82.5–83.2)	6	88.8 (88.1–89.4)	8	5.9	18	71.7 (68.3–74.5)	4	76.0 (72.2–79.2)	7	4.3	19
Ishikawa	82.7 (82.3–83.0)	12	88.3 (87.7–88.9)	16	5.7	25	71.4 (68.2–74.3)	19	75.5 (71.8–78.7)	19	4.1	26
Fukui	82.7 (82.3–83.2)	8	88.4 (87.7–89.1)	13	5.7	26	71.5 (68.1–74.4)	15	75.6 (71.9–78.9)	17	4.1	27
Yamanashi	82.6 (82.2–82.9)	14	88.6 (88.0–89.2)	10	6.0	13	71.4 (68.0–74.2)	16	75.9 (72.3–79.0)	8	4.5	10
Nagano	82.9 (82.6–83.2)	5	88.8 (88.3–89.2)	6	5.9	21	71.6 (68.3–74.5)	6	75.9 (72.1–79.2)	10	4.2	23
Gifu	82.0 (81.7–82.3)	39	88.0 (87.5–88.4)	29	6.0	10	71.0 (67.7–73.9)	37	75.4 (71.8–78.5)	30	4.4	14
Shizuoka	82.7 (82.4–83.0)	9	88.1 (87.6–88.6)	25	5.4	35	71.5 (68.2–74.4)	9	75.4 (71.8–78.5)	28	3.8	37
Aichi	81.9 (81.7–82.1)	40	88.0 (87.7–88.3)	28	6.1	9	70.9 (67.6–73.7)	40	75.3 (71.6–78.6)	33	4.4	12
Mie	82.2 (81.9–82.5)	31	88.1 (87.6–88.6)	24	5.9	16	71.1 (67.8–73.9)	32	75.4 (71.8–78.7)	27	4.3	16
Shiga	82.0 (81.7–82.3)	37	88.9 (88.4–89.4)	5	6.9	1	71.0 (67.7–73.7)	36	76.0 (72.1–79.4)	5	5.0	1
Kyoto	82.4 (82.0–82.7)	25	88.9 (88.4–89.4)	4	6.6	4	71.3 (68.0–74.2)	23	76.1 (72.4–79.3)	4	4.8	4
Osaka	81.2 (81.1–81.4)	47	88.0 (87.7–88.2)	31	6.7	3	70.5 (67.3–73.3)	47	75.5 (72.0–78.8)	20	5.0	3
Hyogo	81.9 (81.6–82.1)	42	88.4 (88.0–88.8)	14	6.5	5	70.8 (67.7–73.6)	41	75.7 (71.9–78.9)	15	4.8	5
Nara	81.9 (81.6–82.2)	41	88.3 (87.8–88.9)	17	6.4	6	71.0 (67.7–73.8)	34	75.7 (72.1–78.9)	12	4.7	6

Wakayama	81.8 (81.4–82.2)	44	87.8 (87.2–88.4)	37	6.0	15	70.8 (67.5–73.6)	43	75.2 (71.4–78.4)	38	4.4	15
Tottori	82.6 (82.2–83.0)	13	87.9 (87.3–88.6)	32	5.3	36	71.5 (68.3–74.4)	10	75.4 (71.6–78.7)	21	3.9	33
Shimane	83.8 (83.4–84.3)	2	89.4 (88.7–90.1)	1	5.6	30	72.5 (69.1–75.3)	2	76.4 (72.5–79.7)	1	4.0	32
Okayama	82.9 (82.6–83.3)	4	89.0 (88.4–89.5)	3	6.0	11	71.7 (68.4–74.5)	5	76.1 (72.4–79.3)	3	4.5	11
Hiroshima	82.5 (82.2–82.8)	21	88.7 (88.2–89.2)	9	6.3	8	71.3 (68.0–74.2)	25	75.9 (72.1–79.0)	9	4.6	8
Yamaguchi	82.6 (82.2–82.9)	17	87.9 (87.3–88.4)	33	5.3	37	71.4 (68.1–74.2)	17	75.3 (71.7–78.6)	31	3.9	35
Tokushima	82.1 (81.7–82.5)	33	87.1 (86.4–87.8)	45	5.0	44	70.9 (67.6–73.7)	38	74.7 (70.8–77.9)	45	3.8	40
Kagawa	82.8 (82.4–83.2)	7	88.3 (87.6–88.9)	19	5.5	33	71.6 (68.3–74.5)	7	75.7 (71.8–79.0)	13	4.1	28
Ehime	82.5 (82.2–82.9)	19	88.1 (87.4–88.7)	26	5.5	31	71.3 (68.0–74.2)	22	75.4 (71.8–78.5)	25	4.1	29
Kochi	82.5 (82.0–82.8)	22	88.1 (87.4–88.8)	27	5.6	28	71.3 (67.9–74.1)	24	75.4 (71.7–78.8)	26	4.1	25
Fukuoka	82.4 (82.2–82.7)	23	88.2 (87.8–88.6)	20	5.8	22	71.0 (67.7–73.9)	33	75.3 (71.4–78.6)	34	4.3	21
Saga	82.7 (82.2–83.1)	11	88.5 (87.8–89.1)	12	5.8	24	71.5 (68.2–74.4)	13	75.8 (72.0–79.2)	11	4.3	17
Nagasaki	82.4 (82.1–82.7)	24	87.8 (87.3–88.4)	35	5.4	34	71.5 (68.3–74.2)	12	75.4 (71.7–78.8)	24	3.9	34
Kumamoto	83.0 (82.6–83.3)	3	88.8 (88.2–89.3)	7	5.8	23	71.7 (68.4–74.5)	3	76.0 (72.2–79.2)	6	4.3	22
Oita	82.3 (82.0–82.7)	26	89.2 (88.6–89.8)	2	6.9	2	71.2 (67.9–74.1)	27	76.2 (72.3–79.7)	2	5.0	2
Miyazaki	82.6 (82.2–82.9)	15	87.6 (87.0–88.2)	38	5.0	45	71.4 (68.1–74.3)	18	75.2 (71.5–78.4)	37	3.8	39
Kagoshima	82.1 (81.8–82.4)	34	88.1 (87.6–88.7)	23	6.0	12	71.1 (67.8–73.9)	31	75.6 (72.0–78.7)	16	4.5	9
Okinawa	84.2 (83.9–84.5)	1	88.3 (87.7–88.9)	18	4.1	47	72.5 (69.0–75.6)	1	75.6 (71.7–78.9)	18	3.1	47

UI: uncertainty interval. Prefectures are ordered according to their ISO codes. The rankings are assigned among the 47 prefectures, with values allocated in descending order from the highest to the lowest.

Table S4: Life expectancy and healthy life expectancy at birth (years) in Japan and 47 prefectures, 1990 and 2021, for males

	Life expectancy (95% UI)						Healthy life expectancy (95% UI)					
Prefecture	1990	Rank	2021	Rank	Increase	Rank	1990	Rank	2021	Rank	Increase	Rank
**Japan**	**76.2 (76.2–76.3)**		**82.2 (82.1–82.2)**		**5.9**		**67.6 (65.1–69.7)**		**72.2 (69.4–74.7)**		**4.7**	
Hokkaido	75.9 (75.7–76.1)	30	81.5 (81.1–81.9)	39	5.6	35	67.3 (64.9–69.4)	29	71.7 (68.9–74.1)	39	4.4	35
Aomori	74.5 (74.1–74.8)	47	79.8 (79.1–80.4)	47	5.3	42	66.0 (63.7–68.0)	47	70.3 (67.5–72.6)	47	4.3	40
Iwate	75.7 (75.3–76.1)	35	81.3 (80.6–81.9)	43	5.5	37	67.2 (64.8–69.3)	33	71.5 (68.7–73.9)	43	4.2	41
Miyagi	76.3 (76.0–76.6)	23	82.5 (81.9–83.0)	15	6.1	13	67.7 (65.2–69.8)	19	72.4 (69.7–75.0)	15	4.8	18
Akita	75.5 (75.1–75.9)	42	81.1 (80.2–81.8)	46	5.6	36	67.1 (64.8–69.2)	38	71.4 (68.5–73.9)	45	4.3	37
Yamagata	76.7 (76.3–77.1)	9	82.0 (81.3–82.7)	26	5.3	43	68.0 (65.5–70.1)	7	72.2 (69.4–74.7)	23	4.2	43
Fukushima	76.0 (75.6–76.3)	29	81.1 (80.6–81.7)	45	5.2	44	67.3 (64.8–69.5)	30	71.4 (68.5–73.8)	46	4.0	44
Ibaraki	75.8 (75.6–76.1)	31	81.7 (81.2–82.1)	34	5.8	25	67.2 (64.7–69.3)	34	71.7 (68.9–74.3)	37	4.6	28
Tochigi	75.6 (75.3–76.0)	38	81.8 (81.2–82.3)	30	6.1	15	67.1 (64.6–69.2)	39	71.9 (69.1–74.4)	30	4.8	14
Gunma	76.7 (76.4–77.1)	10	81.8 (81.2–82.3)	31	5.0	46	68.0 (65.5–70.1)	11	71.9 (69.0–74.4)	32	3.9	47
Saitama	76.5 (76.3–76.8)	15	82.2 (81.8–82.5)	21	5.6	33	67.9 (65.4–70.0)	13	72.3 (69.4–74.7)	21	4.4	34
Chiba	76.7 (76.5–77.0)	8	82.3 (81.9–82.6)	20	5.5	38	68.1 (65.6–70.2)	6	72.3 (69.5–74.7)	19	4.3	39
Tokyo	76.3 (76.1–76.4)	25	82.5 (82.2–82.7)	14	6.2	10	67.5 (65.0–69.7)	27	72.4 (69.4–74.9)	18	4.9	12
Kanagawa	76.8 (76.6–77.0)	5	82.8 (82.5–83.1)	4	6.0	18	68.2 (65.7–70.3)	4	72.9 (70.0–75.2)	4	4.7	19
Niigata	76.7 (76.4–77.1)	6	81.8 (81.2–82.3)	29	5.0	47	68.0 (65.4–70.1)	10	71.9 (68.9–74.4)	31	3.9	46
Toyama	76.5 (76.1–76.9)	18	82.1 (81.4–82.8)	24	5.6	34	67.8 (65.3–70.0)	15	72.2 (69.6–74.7)	22	4.4	33
Ishikawa	76.7 (76.4–77.1)	7	82.7 (82.1–83.3)	6	6.0	19	68.0 (65.5–70.1)	9	72.6 (69.9–75.1)	8	4.7	20
Fukui	77.2 (76.7–77.6)	2	82.7 (81.9–83.4)	8	5.5	40	68.3 (65.8–70.5)	2	72.7 (69.9–75.1)	7	4.3	36
Yamanashi	76.3 (75.9–76.7)	24	82.1 (81.3–82.9)	23	5.8	29	67.6 (65.1–69.8)	23	72.2 (69.4–74.8)	24	4.6	25
Nagano	77.6 (77.3–77.9)	1	83.5 (82.9–84.1)	2	5.9	22	68.6 (66.0–70.8)	1	73.1 (70.1–75.6)	2	4.5	30
Gifu	77.0 (76.6–77.3)	3	82.5 (81.9–83.1)	13	5.5	39	68.2 (65.7–70.4)	3	72.5 (69.7–75.1)	9	4.3	38
Shizuoka	76.9 (76.6–77.2)	4	82.4 (81.9–82.8)	18	5.5	41	68.1 (65.7–70.2)	5	72.3 (69.5–74.9)	20	4.2	42
Aichi	76.6 (76.4–76.8)	12	82.6 (82.3–82.9)	10	6.0	20	67.9 (65.4–70.0)	12	72.5 (69.7–75.0)	11	4.6	22
Mie	76.2 (75.8–76.5)	27	82.4 (81.8–82.9)	19	6.2	11	67.5 (65.1–69.6)	25	72.4 (69.5–75.0)	16	4.9	11
Shiga	76.6 (76.2–76.9)	11	83.7 (83.0–84.3)	1	7.1	1	67.9 (65.4–70.0)	14	73.4 (70.4–75.9)	1	5.5	1
Kyoto	76.6 (76.2–76.9)	13	83.0 (82.4–83.5)	3	6.4	4	68.0 (65.6–70.1)	8	73.0 (70.2–75.4)	3	5.0	8
Osaka	75.1 (74.9–75.3)	46	81.4 (81.2–81.8)	40	6.4	5	66.7 (64.4–68.8)	46	71.8 (69.1–74.2)	34	5.1	4
Hyogo	75.7 (75.5–75.9)	36	82.4 (82.0–82.8)	17	6.7	2	67.2 (64.7–69.3)	35	72.5 (69.7–75.0)	14	5.3	2
Nara	76.4 (76.0–76.8)	20	82.7 (82.1–83.4)	5	6.4	7	67.8 (65.3–70.0)	16	72.8 (69.9–75.4)	5	5.0	6

Wakayama	75.5 (75.1–75.9)	44	81.3 (80.6–82.0)	42	5.8	26	67.0 (64.6–69.1)	42	71.5 (68.6–74.0)	42	4.5	29
Tottori	75.6 (75.2–76.0)	39	81.8 (81.0–82.6)	32	6.1	14	67.1 (64.7–69.2)	37	71.9 (69.0–74.4)	29	4.8	13
Shimane	76.5 (76.0–76.9)	17	82.1 (81.4–83.0)	22	5.7	32	67.6 (65.1–69.8)	22	72.1 (69.3–74.7)	26	4.5	32
Okayama	76.5 (76.1–76.8)	16	82.6 (82.0–83.2)	9	6.1	16	67.7 (65.1–69.9)	18	72.5 (69.8–75.1)	10	4.8	15
Hiroshima	76.3 (76.0–76.7)	21	82.7 (82.2–83.2)	7	6.3	8	67.6 (65.2–69.8)	21	72.7 (69.8–75.2)	6	5.0	5
Yamaguchi	75.8 (75.4–76.2)	32	81.7 (81.0–82.3)	35	5.8	24	67.2 (64.7–69.4)	32	71.8 (68.9–74.4)	33	4.6	23
Tokushima	75.7 (75.2–76.1)	37	81.4 (80.7–82.1)	41	5.7	31	67.0 (64.6–69.0)	40	71.6 (68.8–74.2)	41	4.6	26
Kagawa	76.3 (75.9–76.7)	22	82.5 (81.8–83.2)	12	6.2	12	67.6 (65.0–69.8)	24	72.5 (69.5–74.9)	13	4.9	9
Ehime	76.1 (75.7–76.5)	28	81.8 (81.1–82.5)	28	5.7	30	67.4 (64.9–69.6)	28	72.0 (69.1–74.5)	28	4.6	27
Kochi	75.6 (75.2–76.1)	41	81.7 (80.9–82.4)	33	6.1	17	67.0 (64.6–69.1)	41	71.8 (69.0–74.2)	35	4.8	16
Fukuoka	75.4 (75.2–75.6)	45	82.0 (81.7–82.4)	25	6.6	3	66.8 (64.4–68.9)	45	72.0 (69.2–74.4)	27	5.2	3
Saga	75.8 (75.3–76.2)	33	82.0 (81.2–82.8)	27	6.2	9	67.2 (64.7–69.5)	31	72.2 (69.3–74.7)	25	4.9	10
Nagasaki	75.5 (75.1–75.9)	43	81.3 (80.6–82.0)	44	5.8	28	67.0 (64.5–69.1)	44	71.5 (68.7–73.8)	44	4.5	31
Kumamoto	76.6 (76.2–76.9)	14	82.4 (81.8–83.0)	16	5.9	23	67.7 (65.2–69.9)	17	72.4 (69.5–74.9)	17	4.6	21
Oita	76.2 (75.8–76.6)	26	82.5 (81.9–83.2)	11	6.4	6	67.5 (65.0–69.6)	26	72.5 (69.7–75.0)	12	5.0	7
Miyazaki	75.6 (75.2–76.1)	40	81.5 (80.8–82.2)	36	5.9	21	67.0 (64.6–69.1)	43	71.7 (69.1–74.2)	36	4.8	17
Kagoshima	75.7 (75.4–76.1)	34	81.5 (80.9–82.2)	37	5.8	27	67.1 (64.7–69.5)	36	71.7 (69.0–74.3)	38	4.6	24
Okinawa	76.4 (76.0–76.9)	19	81.5 (80.9–82.1)	38	5.1	45	67.7 (65.1–70.0)	20	71.6 (68.8–74.1)	40	4.0	45

UI: uncertainty interval. Prefectures are ordered according to their ISO codes. The rankings are assigned among the 47 prefectures, with values allocated in descending order from the highest to the lowest.

Table S5: Age-standardised mortality and DALY rates per 100,000 population in Japan and 47 Prefectures: 1990 and 2021 values with percent change (%), for both sexes

	Age-standardised mortality rate per 100,000 (95% UI)						Age-standardised DALY rate per 100,000 (95% UI)					
Prefecture	1990	Rank	2021	Rank	Percent change between 1990 and 2021 (%)	Rank	1990	Rank	2021	Rank	Percent change between 1990 and 2021 (%)	Rank
**Japan**	**529.3 (527.5–531.0)**		**311.1 (309.5–312.6)**		**-41.2**		**21449.6 (19032.9–24332.4)**		**16186.7 (13729.3–18997.2)**		**-24.5**	
Hokkaido	535.7 (527.6–543.5)	31	328.7 (319.1–337.6)	40	-38.6	36	21788.5 (19419.8–24661.9)	34	16820.3 (14491.1–19561.2)	40	-22.8	37
Aomori	570.7 (558.2–584.7)	46	369.1 (353.6–384.4)	47	-35.3	46	23128.7 (20739.2–26004.1)	47	17935.0 (15519.0–20797.4)	47	-22.5	39
Iwate	542.0 (530.2–554.4)	39	336.5 (321.9–352.1)	43	-37.9	38	21851.3 (19502.1–24828.4)	35	16927.3 (14497.8–19684.7)	43	-22.5	38
Miyagi	532.7 (521.2–544.3)	28	305.4 (293.7–316.6)	15	-42.7	11	21278.2 (18845.8–24118.7)	19	16154.1 (13708.5–18928.8)	21	-24.1	28
Akita	554.7 (540.9–568.7)	42	346.2 (328.0–363.6)	46	-37.6	42	21923.9 (19508.7–24642.8)	38	17144.8 (14742.0–19967.5)	46	-21.8	43
Yamagata	514.8 (502.1–528.4)	13	320.5 (306.8–336.7)	33	-37.7	41	20770.6 (18436.9–23627.3)	3	16502.6 (14096.2–19319.3)	32	-20.5	46
Fukushima	530.5 (519.4–541.8)	27	341.1 (329.1–354.7)	45	-35.7	45	21653.9 (19209.7–24479.6)	30	17038.1 (14614.2–19790.9)	45	-21.3	44
Ibaraki	556.2 (545.8–566.8)	43	331.1 (321.1–342.7)	42	-40.5	24	22148.7 (19713.7–25106.6)	43	16838.1 (14357.9–19773.7)	41	-24.0	29
Tochigi	565.5 (554.0–576.7)	45	330.1 (318.8–342.1)	41	-41.6	16	22394.3 (19960.7–25208.4)	45	16710.5 (14309.3–19471.6)	38	-25.4	13
Gunma	529.5 (518.7–541.8)	25	328.7 (317.0–340.9)	39	-37.9	37	21291.9 (18864.2–24125.3)	20	16631.4 (14131.9–19506.8)	36	-21.9	42
Saitama	535.9 (528.0–544.2)	32	317.6 (309.5–325.4)	27	-40.7	22	21195.8 (18773.6–24028.0)	16	16166.6 (13779.6–18954.7)	23	-23.7	32
Chiba	516.9 (508.6–525.3)	14	311.1 (304.1–318.6)	25	-39.8	28	20822.9 (18395.8–23702.0)	6	16165.8 (13809.8–18955.3)	22	-22.4	40
Tokyo	534.5 (528.7–540.1)	29	300.9 (296.0–306.0)	10	-43.7	7	21612.6 (19157.5–24488.8)	28	16053.1 (13516.6–18934.3)	18	-25.7	10
Kanagawa	514.2 (507.4–521.3)	12	298.9 (292.7–306.6)	9	-41.9	15	20842.8 (18400.0–23651.1)	7	15740.3 (13391.5–18586.7)	4	-24.5	19
Niigata	512.5 (500.4–523.6)	11	318.9 (306.8–331.4)	30	-37.8	39	20812.1 (18433.3–23708.4)	5	16379.2 (13926.6–19258.8)	28	-21.3	45
Toyama	505.8 (494.2–518.7)	7	305.9 (291.2–319.7)	17	-39.5	31	21091.1 (18635.4–23956.7)	12	15976.8 (13544.6–18606.4)	15	-24.2	23
Ishikawa	509.7 (497.8–522.0)	9	301.5 (288.4–315.3)	12	-40.8	20	20947.9 (18535.7–23786.4)	9	15788.1 (13411.1–18472.1)	6	-24.6	18
Fukui	496.0 (483.3–509.4)	4	301.1 (285.5–317.9)	11	-39.3	32	20732.9 (18255.4–23676.7)	2	15843.6 (13489.3–18622.4)	10	-23.6	33
Yamanashi	519.2 (506.6–533.4)	16	307.3 (292.5–323.1)	18	-40.8	21	21446.3 (19082.0–24341.4)	24	16133.7 (13671.5–18800.6)	20	-24.8	16
Nagano	487.5 (476.7–498.1)	3	281.1 (270.6–292.5)	2	-42.3	14	20392.0 (17928.2–23177.0)	1	15662.6 (13199.7–18391.2)	3	-23.2	35
Gifu	527.9 (517.0–539.1)	23	308.2 (296.2–320.3)	22	-41.6	17	21019.3 (18506.4–23878.5)	10	15929.1 (13426.0–18674.9)	13	-24.2	26
Shizuoka	504.7 (496.0–513.4)	6	309.1 (299.7–318.6)	24	-38.8	35	20792.4 (18384.2–23596.0)	4	16172.0 (13753.4–18951.1)	24	-22.2	41
Aichi	539.9 (531.9–547.8)	37	307.4 (300.2–315.0)	19	-43.1	9	21232.0 (18826.8–24053.0)	17	15891.2 (13457.1–18709.5)	11	-25.2	15

Mie	538.3 (525.7–550.3)	35	309.1 (295.9–322.9)	23	-42.6	12	21560.2 (19005.4–24382.7)	25	15995.9 (13514.6–18755.4)	16	-25.8	9
Shiga	538.9 (525.9–551.9)	36	274.8 (263.2–287.0)	1	-49.0	1	21267.4 (18805.7–24156.1)	18	15378.1 (12922.9–18253.5)	1	-27.7	1
Kyoto	518.4 (507.6–529.3)	15	288.5 (278.9–298.6)	4	-44.4	5	21093.1 (18680.6–23930.8)	13	15614.4 (13230.1–18372.0)	2	-26.0	7
Osaka	584.9 (577.6–591.9)	47	325.8 (319.1–332.3)	35	-44.3	6	22564.1 (20130.9–25386.0)	46	16380.2 (13977.7–19200.8)	29	-27.4	2
Hyogo	553.7 (545.8–561.9)	41	301.8 (293.6–309.3)	13	-45.5	2	21920.8 (19530.1–24727.0)	37	15924.0 (13489.7–18633.8)	12	-27.4	3
Nara	542.2 (529.3–555.3)	40	296.3 (283.5–310.1)	8	-45.4	4	21333.8 (18809.3–24176.8)	21	15818.5 (13422.9–18646.2)	8	-25.9	8
Wakayama	559.5 (546.3–574.5)	44	327.7 (313.1–342.4)	38	-41.4	18	22222.0 (19723.6–25118.6)	44	16573.0 (14054.1–19515.9)	33	-25.4	11
Tottori	529.9 (516.5–545.0)	26	318.1 (302.2–335.3)	29	-40.0	27	21644.6 (19215.4–24502.5)	29	16358.3 (13943.1–19246.8)	27	-24.4	20
Shimane	483.9 (471.1–497.5)	2	302.0 (286.4–317.3)	14	-37.6	43	20921.3 (18519.5–23804.1)	8	16033.0 (13568.1–18818.0)	17	-23.4	34
Okayama	507.3 (495.1–519.3)	8	291.7 (280.7–303.0)	5	-42.5	13	21149.3 (18687.7–24071.5)	15	15794.9 (13413.5–18561.1)	7	-25.3	14
Hiroshima	522.8 (512.8–533.0)	19	294.4 (283.4–305.2)	6	-43.7	8	21421.4 (18916.0–24192.0)	23	15755.6 (13318.4–18584.6)	5	-26.4	5
Yamaguchi	528.2 (516.7–541.0)	24	319.0 (305.1–333.3)	31	-39.6	30	21669.1 (19223.7–24553.4)	31	16435.9 (14063.6–19272.9)	31	-24.2	27
Tokushima	540.0 (526.4–554.1)	38	340.4 (324.1–357.4)	44	-37.0	44	22120.3 (19693.6–24986.1)	41	16854.2 (14309.5–19846.0)	42	-23.8	31
Kagawa	510.2 (498.0–523.3)	10	305.5 (292.2–318.9)	16	-40.1	26	21349.5 (18766.4–24187.0)	22	16085.3 (13730.4–18964.1)	19	-24.7	17
Ehime	520.5 (508.1–533.3)	17	316.0 (301.9–332.0)	26	-39.3	33	21599.4 (19089.0–24433.4)	27	16421.6 (13973.8–19158.6)	30	-24.0	30
Kochi	526.7 (512.9–539.6)	21	317.9 (302.0–334.4)	28	-39.7	29	22101.2 (19669.3–24978.4)	40	16718.5 (14313.8–19523.7)	39	-24.4	21
Fukuoka	537.0 (529.2–545.7)	34	307.8 (299.8–315.6)	21	-42.7	10	22130.3 (19671.1–25044.6)	42	16307.9 (13881.0–19246.5)	26	-26.3	6
Saga	521.0 (507.0–535.0)	18	307.6 (293.4–323.9)	20	-40.9	19	21697.3 (19119.7–24554.3)	32	16185.4 (13676.9–19061.1)	25	-25.4	12
Nagasaki	536.5 (524.9–550.1)	33	326.4 (311.7–341.3)	37	-39.2	34	21721.9 (19297.1–24613.7)	33	16691.4 (14289.6–19457.1)	37	-23.2	36
Kumamoto	497.7 (486.6–509.3)	5	296.0 (283.8–308.9)	7	-40.5	23	21029.6 (18618.2–23970.7)	11	15931.5 (13427.6–18750.8)	14	-24.2	24
Oita	527.0 (515.0–539.5)	22	287.9 (275.1–301.5)	3	-45.4	3	21560.6 (19089.9–24428.7)	26	15835.0 (13368.6–18681.7)	9	-26.6	4
Miyazaki	523.4 (509.7–537.4)	20	325.8 (311.0–342.5)	36	-37.8	40	21945.9 (19521.6–24782.4)	39	16626.0 (14215.3–19288.5)	35	-24.2	25
Kagoshima	535.7 (523.8–549.1)	30	320.0 (307.8–333.6)	32	-40.3	25	21908.8 (19354.5–24868.7)	36	16585.1 (14107.1–19347.0)	34	-24.3	22
Okinawa	457.8 (447.0–468.4)	1	324.5 (310.3–337.2)	34	-29.1	47	21110.4 (18562.9–23998.1)	14	16967.8 (14550.1–19789.5)	44	-19.6	47

UI: uncertainty interval. Prefectures are ordered according to their ISO codes.The rankings are assigned among the 47 prefectures, with values allocated in ascending order from the lowest to the highest.

Table S6: COVID-19 mortality rate, percentage of total deaths, and COVID-19 age-standardised mortality rate in Japan and 47 prefectures, 2020 and 2021, for both sexes

	2020			2021		
Prefecture	Mortality rate per 100,000 (95% UI)	Percent of total deaths (%) (95% UI)	Age-standardised mortality rate per 100,000 (95% UI)	Mortality rate per 100,000 (95% UI)	Percent of total deaths (%) (95% UI)	Age-standardised mortality rate per 100,000 (95% UI)
**Japan**	2.7 (2.4–3.1)	0.3 (0.2–0.3)	0.8 (0.7–0.9)	11.7 (9.8–13.9)	1.0 (0.9–1.2)	3.0 (2.5–3.7)
Hokkaido	8.3 (3.8–13.1)	0.7 (0.3–1.1)	2.2 (1.0–3.4)	19.9 (7.1–39.7)	1.5 (0.6–3.1)	4.7 (1.5–9.6)
Aomori	0.6 (0.2–1.1)	0.0 (0.0–0.1)	0.2 (0.1–0.3)	2.3 (0.7–4.6)	0.2 (0.0–0.3)	0.5 (0.2–1.1)
Iwate	2.0 (0.9–3.4)	0.1 (0.1–0.2)	0.5 (0.2–0.8)	2.2 (0.6–4.4)	0.1 (0.0–0.3)	0.5 (0.1–1.0)
Miyagi	0.7 (0.3–1.2)	0.1 (0.0–0.1)	0.2 (0.1–0.3)	3.9 (1.2–8.2)	0.4 (0.1–0.7)	1.1 (0.3–2.4)
Akita	0.1 (0.0–0.2)	0.0 (0.0–0.0)	0.0 (0.0–0.0)	11.5 (1.5–42.3)	0.7 (0.1–2.5)	2.0 (0.2–7.8)
Yamagata	0.6 (0.2–1.0)	0.0 (0.0–0.1)	0.1 (0.1–0.2)	5.0 (1.8–9.4)	0.3 (0.1–0.6)	0.9 (0.2–1.9)
Fukushima	1.2 (0.6–2.2)	0.1 (0.0–0.2)	0.3 (0.1–0.6)	7.8 (2.6–15.2)	0.6 (0.2–1.1)	1.6 (0.5–3.3)
Ibaraki	1.2 (0.6–2.0)	0.1 (0.1–0.2)	0.4 (0.2–0.6)	5.8 (1.3–11.6)	0.5 (0.1–1.0)	1.5 (0.3–3.2)
Tochigi	0.3 (0.1–0.5)	0.0 (0.0–0.0)	0.1 (0.0–0.2)	5.2 (1.8–9.9)	0.4 (0.2–0.8)	1.4 (0.5–2.8)
Gunma	2.3 (1.0–3.8)	0.2 (0.1–0.3)	0.6 (0.2–1.0)	12.4 (2.9–36.6)	1.0 (0.2–2.9)	3.0 (0.7–8.9)
Saitama	2.8 (1.2–4.4)	0.3 (0.1–0.5)	0.9 (0.4–1.4)	11.8 (3.9–28.0)	1.2 (0.4–2.8)	3.4 (1.0–8.3)
Chiba	1.8 (0.8–3.0)	0.2 (0.1–0.3)	0.6 (0.3–1.0)	13.1 (4.6–22.4)	1.3 (0.4–2.2)	3.5 (1.1–6.1)
Tokyo	4.3 (2.4–6.2)	0.5 (0.3–0.7)	1.5 (0.8–2.2)	15.9 (7.1–25.4)	1.8 (0.8–2.9)	5.1 (2.1–8.4)
Kanagawa	2.9 (1.4–4.5)	0.3 (0.2–0.5)	1.0 (0.5–1.5)	10.2 (3.1–21.3)	1.1 (0.3–2.2)	3.1 (0.9–6.5)
Niigata	0.1 (0.0–0.2)	0.0 (0.0–0.0)	0.0 (0.0–0.1)	5.9 (0.7–24.1)	0.4 (0.1–1.7)	1.1 (0.1–5.0)
Toyama	3.1 (1.5–5.1)	0.3 (0.1–0.4)	0.7 (0.3–1.1)	14.9 (1.9–42.5)	1.1 (0.1–3.2)	3.0 (0.4–8.7)
Ishikawa	5.9 (3.3–8.8)	0.5 (0.3–0.8)	1.3 (0.6–2.0)	16.6 (6.6–39.4)	1.4 (0.6–3.3)	3.4 (1.3–8.6)
Fukui	2.0 (0.9–3.3)	0.2 (0.1–0.3)	0.5 (0.2–0.8)	13.8 (2.3–42.8)	1.1 (0.2–3.3)	3.0 (0.4–9.3)
Yamanashi	1.4 (0.7–2.4)	0.1 (0.1–0.2)	0.3 (0.2–0.6)	2.4 (0.9–4.3)	0.2 (0.1–0.3)	0.6 (0.2–1.1)
Nagano	0.7 (0.3–1.2)	0.1 (0.0–0.1)	0.2 (0.1–0.3)	3.6 (1.1–7.4)	0.3 (0.1–0.6)	0.9 (0.2–1.8)
Gifu	1.7 (0.7–2.9)	0.1 (0.1–0.3)	0.5 (0.2–0.8)	8.4 (2.7–18.4)	0.7 (0.2–1.5)	1.7 (0.5–3.9)
Shizuoka	1.1 (0.5–1.9)	0.1 (0.0–0.2)	0.3 (0.1–0.5)	4.2 (1.0–8.3)	0.4 (0.1–0.7)	1.1 (0.3–2.2)
Aichi	2.7 (1.4–4.3)	0.3 (0.2–0.5)	0.9 (0.4–1.4)	11.1 (4.3–20.5)	1.1 (0.4–2.1)	3.1 (1.1–6.0)
Mie	1.0 (0.5–1.8)	0.1 (0.0–0.2)	0.3 (0.1–0.5)	9.8 (2.9–28.4)	0.8 (0.2–2.3)	2.2 (0.6–6.6)
Shiga	0.9 (0.4–1.4)	0.1 (0.0–0.1)	0.3 (0.1–0.5)	6.1 (3.0–12.8)	0.6 (0.3–1.3)	1.5 (0.7–3.2)
Kyoto	1.8 (0.7–3.1)	0.2 (0.1–0.3)	0.5 (0.2–0.9)	11.1 (3.7–26.9)	1.0 (0.3–2.5)	2.5 (0.7–6.1)
Osaka	6.3 (3.3–9.3)	0.6 (0.3–0.9)	1.9 (1.0–2.8)	24.1 (11.0–39.5)	2.2 (1.0–3.7)	6.7 (2.9–11.5)
Hyogo	3.8 (2.0–5.8)	0.4 (0.2–0.5)	0.9 (0.4–1.4)	18.3 (5.9–38.5)	1.7 (0.5–3.4)	4.8 (1.3–10.5)
Nara	1.8 (0.7–3.3)	0.2 (0.1–0.3)	0.5 (0.2–0.9)	8.6 (3.1–15.8)	0.7 (0.3–1.3)	2.2 (0.8–4.2)
Wakayama	0.8 (0.4–1.5)	0.1 (0.0–0.1)	0.2 (0.1–0.4)	5.5 (1.8–10.8)	0.4 (0.1–0.7)	1.2 (0.4–2.5)
Tottori	0.0 (0.0–0.0)	0.0 (0.0–0.0)	0.0 (0.0–0.0)	0.9 (0.3–1.7)	0.1 (0.0–0.1)	0.2 (0.1–0.4)
Shimane	0.0 (0.0–0.0)	0.0 (0.0–0.0)	0.0 (0.0–0.0)	10.2 (0.6–41.6)	0.7 (0.0–2.9)	1.8 (0.1–7.9)
Okayama	0.7 (0.3–1.2)	0.1 (0.0–0.1)	0.2 (0.1–0.3)	5.9 (2.7–10.3)	0.5 (0.2–0.9)	1.4 (0.6–2.6)
Hiroshima	1.0 (0.4–1.9)	0.1 (0.0–0.2)	0.3 (0.1–0.5)	5.3 (1.5–11.0)	0.5 (0.1–1.0)	1.4 (0.4–2.9)
Yamaguchi	0.2 (0.1–0.4)	0.0 (0.0–0.0)	0.1 (0.0–0.1)	7.4 (1.6–22.8)	0.5 (0.1–1.6)	1.3 (0.2–4.2)
Tokushima	1.3 (0.7–2.0)	0.1 (0.1–0.2)	0.3 (0.2–0.5)	20.1 (6.4–50.4)	1.3 (0.4–3.3)	3.7 (1.0–9.7)
Kagawa	0.4 (0.2–0.6)	0.0 (0.0–0.0)	0.1 (0.0–0.2)	3.9 (1.7–7.1)	0.3 (0.1–0.5)	0.8 (0.3–1.6)
Ehime	1.0 (0.4–1.8)	0.1 (0.0–0.1)	0.2 (0.1–0.4)	9.8 (2.3–30.5)	0.7 (0.2–2.2)	1.9 (0.4–6.2)
Kochi	1.0 (0.4–1.7)	0.1 (0.0–0.1)	0.2 (0.1–0.4)	6.6 (1.5–24.5)	0.4 (0.1–1.6)	1.1 (0.2–4.3)
Fukuoka	2.3 (1.0–3.8)	0.2 (0.1–0.4)	0.7 (0.3–1.1)	9.8 (3.4–20.9)	0.9 (0.3–1.9)	2.4 (0.7–5.3)
Saga	0.4 (0.2–0.7)	0.0 (0.0–0.1)	0.1 (0.1–0.2)	3.0 (0.9–6.4)	0.2 (0.1–0.5)	0.7 (0.2–1.6)
Nagasaki	0.3 (0.1–0.5)	0.0 (0.0–0.0)	0.1 (0.0–0.1)	11.3 (2.4–36.8)	0.8 (0.2–2.7)	2.3 (0.4–7.8)
Kumamoto	0.9 (0.4–1.6)	0.1 (0.0–0.1)	0.2 (0.1–0.4)	7.3 (3.0–13.1)	0.6 (0.2–1.0)	1.5 (0.5–2.8)
Oita	0.7 (0.3–1.2)	0.1 (0.0–0.1)	0.2 (0.1–0.3)	6.0 (1.9–18.2)	0.5 (0.1–1.4)	1.5 (0.5–4.6)
Miyazaki	2.7 (0.1–17.2)	0.2 (0.0–1.4)	0.6 (0.0–4.1)	8.7 (1.6–30.3)	0.6 (0.1–2.2)	1.8 (0.3–6.6)
Kagoshima	0.9 (0.4–1.5)	0.1 (0.0–0.1)	0.2 (0.1–0.4)	3.0 (1.2–5.7)	0.2 (0.1–0.4)	0.7 (0.3–1.4)
Okinawa	5.5 (2.6–8.9)	0.7 (0.3–1.1)	2.0 (0.9–3.3)	24.7 (10.6–45.7)	2.8 (1.2–5.1)	8.8 (3.6–16.9)

UI: uncertainty interval. Prefectures are ordered according to their ISO codes.

Table S7: COVID-19 DALY rate, percentage of total DALYs, and COVID-19 age-standardised DALY rate in Japan and 47 prefectures, 2020 and 2021, for both sexes

	2020			2021		
Prefecture	DALY rate per 100,000 (95% UI)	Percent of total DALY (%) (95% UI)	Age-standardised DALY rate per 100,000 (95% UI)	DALY rate per 100,000 (95% UI)	Percent of total DALYs (%) (95% UI)	Age-standardised DALY rate per 100,000 (95% UI)
**Japan**	45.1 (40.0–51.9)	0.2 (0.1–0.2)	19.2 (16.5–23.4)	190.2 (155.6–235.0)	0.6 (0.5–0.8)	79.4 (59.8–113.0)
Hokkaido	130.4 (62.2–202.3)	0.4 (0.2–0.6)	48.1 (24.2–75.1)	306.9 (119.9–611.0)	0.9 (0.4–1.8)	112.5 (47.7–214.1)
Aomori	13.4 (6.1–22.0)	0.0 (0.0–0.1)	6.8 (3.5–11.8)	61.9 (30.9–119.0)	0.2 (0.1–0.3)	35.9 (15.9–73.4)
Iwate	33.1 (16.6–53.6)	0.1 (0.0–0.2)	13.6 (7.5–21.5)	60.2 (29.9–104.4)	0.2 (0.1–0.3)	34.5 (16.3–68.1)
Miyagi	14.4 (7.3–23.3)	0.0 (0.0–0.1)	7.3 (3.8–12.3)	86.4 (38.8–154.7)	0.3 (0.1–0.5)	44.9 (19.7–81.2)
Akita	5.7 (3.0–10.1)	0.0 (0.0–0.0)	3.7 (1.7–7.7)	177.3 (40.2–576.2)	0.5 (0.1–1.5)	62.2 (19.0–164.7)
Yamagata	11.7 (6.2–19.2)	0.0 (0.0–0.1)	6.2 (3.3–10.6)	90.3 (41.6–157.5)	0.3 (0.1–0.5)	43.1 (19.6–81.9)
Fukushima	22.8 (12.1–37.4)	0.1 (0.0–0.1)	10.2 (5.7–16.6)	127.7 (54.2–228.4)	0.4 (0.2–0.7)	54.5 (24.9–96.2)
Ibaraki	23.6 (12.7–36.2)	0.1 (0.0–0.1)	11.8 (6.9–17.6)	111.0 (35.2–197.3)	0.3 (0.1–0.6)	53.0 (21.1–99.3)
Tochigi	8.5 (4.5–14.1)	0.0 (0.0–0.0)	5.1 (2.6–9.3)	104.5 (48.4–183.0)	0.3 (0.2–0.6)	51.0 (24.4–90.2)
Gunma	35.4 (16.8–59.7)	0.1 (0.1–0.2)	15.0 (7.7–24.2)	195.8 (61.4–529.3)	0.6 (0.2–1.6)	82.2 (30.5–194.5)
Saitama	47.0 (23.0–73.5)	0.2 (0.1–0.3)	21.1 (10.6–32.4)	195.3 (76.2–438.0)	0.7 (0.3–1.5)	81.1 (34.7–179.8)
Chiba	33.6 (17.1–52.0)	0.1 (0.1–0.2)	14.8 (7.5–23.6)	204.1 (82.9–344.5)	0.7 (0.3–1.2)	80.0 (36.0–134.4)
Tokyo	70.4 (40.4–102.9)	0.3 (0.2–0.4)	32.2 (19.0–47.9)	256.2 (117.8–407.6)	1.0 (0.4–1.5)	117.3 (53.6–187.0)
Kanagawa	52.9 (28.8–79.9)	0.2 (0.1–0.3)	24.4 (13.8–36.1)	178.8 (65.5–340.2)	0.6 (0.2–1.2)	81.6 (33.2–153.2)
Niigata	5.9 (3.1–10.6)	0.0 (0.0–0.0)	3.9 (1.8–8.1)	100.0 (31.0–349.1)	0.3 (0.1–1.0)	42.8 (14.9–108.2)
Toyama	44.8 (22.9–73.1)	0.1 (0.1–0.2)	15.8 (8.3–24.8)	218.0 (47.3–568.9)	0.7 (0.1–1.7)	77.7 (22.3–187.5)
Ishikawa	78.9 (40.4–118.6)	0.3 (0.1–0.4)	27.8 (14.0–43.7)	229.9 (97.8–526.4)	0.7 (0.3–1.7)	80.5 (35.6–168.0)
Fukui	31.7 (16.0–51.6)	0.1 (0.1–0.2)	13.6 (7.7–21.2)	205.9 (51.0–576.0)	0.6 (0.2–1.9)	79.3 (24.8–194.8)
Yamanashi	23.5 (12.4–40.2)	0.1 (0.0–0.1)	11.0 (6.4–18.1)	64.5 (30.5–112.7)	0.2 (0.1–0.3)	36.7 (17.8–71.9)
Nagano	15.3 (8.2–24.0)	0.0 (0.0–0.1)	7.5 (4.4–12.5)	81.8 (34.9–146.6)	0.3 (0.1–0.5)	41.8 (19.1–79.2)
Gifu	28.5 (13.3–46.6)	0.1 (0.0–0.2)	13.6 (7.6–21.0)	127.3 (55.1–255.8)	0.4 (0.2–0.8)	48.6 (22.5–97.0)
Shizuoka	20.8 (9.9–33.4)	0.1 (0.0–0.1)	9.9 (5.2–15.6)	88.7 (37.2–162.7)	0.3 (0.1–0.5)	44.3 (19.7–86.8)
Aichi	46.9 (25.2–72.4)	0.2 (0.1–0.3)	21.7 (12.1–32.8)	174.7 (75.5–316.0)	0.6 (0.3–1.1)	74.3 (32.3–131.3)
Mie	19.1 (10.3–31.2)	0.1 (0.0–0.1)	9.3 (5.2–15.3)	155.8 (57.3–398.9)	0.5 (0.2–1.2)	63.7 (27.7–134.0)
Shiga	16.9 (8.9–26.1)	0.1 (0.0–0.1)	8.9 (5.0–14.0)	101.5 (52.8–186.8)	0.4 (0.2–0.7)	50.6 (26.5–92.4)
Kyoto	31.5 (14.3–52.1)	0.1 (0.1–0.2)	14.2 (7.3–23.5)	173.9 (65.3–370.8)	0.6 (0.2–1.2)	69.7 (29.5–137.9)
Osaka	99.4 (52.3–146.2)	0.3 (0.2–0.5)	40.7 (21.7–59.7)	370.4 (169.6–616.5)	1.2 (0.5–2.1)	145.2 (65.2–245.0)
Hyogo	50.3 (26.9–77.8)	0.2 (0.1–0.3)	16.5 (9.2–26.1)	290.8 (103.1–601.3)	1.0 (0.3–1.9)	117.0 (45.8–234.5)
Nara	31.8 (15.2–54.7)	0.1 (0.1–0.2)	14.4 (8.2–23.3)	154.4 (66.9–267.0)	0.5 (0.2–0.9)	68.6 (33.3–115.4)
Wakayama	15.7 (7.8–26.3)	0.0 (0.0–0.1)	7.3 (3.9–12.4)	103.6 (45.0–200.8)	0.3 (0.1–0.6)	47.8 (21.9–91.7)
Tottori	3.8 (1.6–8.6)	0.0 (0.0–0.0)	3.1 (1.2–7.5)	41.8 (21.6–84.3)	0.1 (0.1–0.2)	28.1 (12.2–64.9)
Shimane	3.9 (1.7–8.5)	0.0 (0.0–0.0)	3.2 (1.2–7.5)	157.5 (25.3–566.9)	0.5 (0.1–1.5)	60.4 (13.6–176.8)
Okayama	13.6 (6.8–22.1)	0.0 (0.0–0.1)	6.8 (3.7–11.9)	110.7 (57.2–185.3)	0.4 (0.2–0.6)	52.8 (27.6–93.4)
Hiroshima	19.3 (9.0–32.6)	0.1 (0.0–0.1)	9.2 (4.6–14.9)	104.6 (39.7–195.4)	0.3 (0.1–0.6)	50.7 (20.6–89.5)
Yamaguchi	7.4 (3.8–12.4)	0.0 (0.0–0.0)	4.6 (2.3–8.8)	119.1 (44.3–313.5)	0.3 (0.1–0.9)	47.1 (20.5–105.0)
Tokushima	21.5 (12.7–34.0)	0.1 (0.0–0.1)	9.7 (5.7–15.1)	274.1 (99.1–672.9)	0.8 (0.3–1.8)	91.1 (34.9–209.4)
Kagawa	9.1 (5.3–14.4)	0.0 (0.0–0.0)	5.0 (2.7–9.2)	80.6 (40.2–142.4)	0.2 (0.1–0.4)	40.3 (19.3–78.7)
Ehime	17.7 (8.6–29.5)	0.1 (0.0–0.1)	7.9 (4.0–13.2)	150.2 (47.3–424.3)	0.4 (0.1–1.2)	58.0 (21.4–138.3)
Kochi	19.2 (10.5–29.8)	0.1 (0.0–0.1)	8.6 (5.0–13.6)	109.2 (40.5–324.6)	0.3 (0.1–0.9)	43.6 (15.9–99.2)
Fukuoka	37.8 (18.0–60.4)	0.1 (0.1–0.2)	16.9 (8.8–26.5)	154.1 (63.1–311.8)	0.5 (0.2–1.0)	63.0 (27.3–117.9)
Saga	10.2 (5.7–15.6)	0.0 (0.0–0.1)	5.4 (2.9–9.4)	71.5 (34.7–130.9)	0.2 (0.1–0.4)	39.7 (19.8–77.3)
Nagasaki	8.2 (4.5–13.8)	0.0 (0.0–0.0)	4.9 (2.5–9.2)	175.6 (51.6–523.0)	0.5 (0.2–1.5)	68.1 (25.5–177.6)
Kumamoto	16.9 (8.5–28.3)	0.1 (0.0–0.1)	8.1 (4.4–13.4)	120.4 (57.2–204.0)	0.4 (0.2–0.6)	50.2 (23.6–89.8)
Oita	13.3 (7.0–22.3)	0.0 (0.0–0.1)	6.5 (3.6–11.7)	118.8 (50.3–310.1)	0.4 (0.2–0.9)	58.4 (27.5–127.5)
Miyazaki	40.8 (4.3–237.6)	0.1 (0.0–0.8)	17.5 (2.6–89.5)	142.4 (43.8–449.1)	0.4 (0.1–1.3)	59.8 (21.2–165.8)
Kagoshima	16.9 (9.2–27.6)	0.1 (0.0–0.1)	8.3 (4.9–13.7)	72.7 (36.5–128.9)	0.2 (0.1–0.4)	39.7 (19.2–80.1)
Okinawa	92.7 (45.0–147.9)	0.4 (0.2–0.5)	45.5 (22.1–72.2)	425.3 (182.6–783.2)	1.6 (0.7–2.8)	209.0 (89.6–375.9)

UI: uncertainty interval. Prefectures are ordered according to their ISO codes.
