# Supplementary material for: Three decades of population health changes in Japan, 1990–2021: a subnational analysis for the Global Burden of Disease Study 2021
Source: Lancet Public Health. 2025 Mar 20;10(4):e321–32. doi: 10.1016/S2468-2667(25)00044-1 (PMC11959113; doi:10.1016/S2468-2667(25)00044-1)
Supplement: Supplementary appendix 2 [file mmc2.pdf]

THE LANCET
Public Health

Supplementary appendix 2

This appendix formed part of the original submission and has been peer reviewed. We post it as supplied by the authors.

Supplement to: GBD 2021 Japan Collaborators. Three decades of population health changes in Japan, 1990–2021: a subnational analysis for the Global Burden of Disease Study 2021. *Lancet Public Health* 2025; published online March 20. https://doi.org/10.1016/S2468-2667(25)00044-1.

Appendix 2: Authorship appendix to “Three decades of population health changes in Japan, 1990-2021: a subnational analysis for the Global Burden of Disease Study 2021”

This appendix provides further authorship detail for “Three decades of population health changes in Japan, 1990-2021: a subnational analysis for the Global Burden of Disease Study 2021”

Table of Contents

Appendix 2: Authorship appendix to “Three decades of population health changes in Japan, 1990-2021: a subnational analysis for the Global Burden of Disease Study 2021”

GBD 2021 Japan BoD Collaborators 2

Affiliations 2

Authors’ Contributions 6

Providing data or critical feedback on data sources 6

Developing methods or computational machinery 6

Providing critical feedback on methods or results 6

Drafting the work or revising it critically for important intellectual content 7

Managing the estimation or publications process 7

GBD 2021 Japan BoD Collaborators

Shuhei Nomura, Michio Murakami, Santosh Kumar Rauniyar, Naoki Kondo, Takahiro Tabuchi, Haruka Sakamoto, Yasuharu Tokuda, Nishali Patel, Jose Navarro de Pablo, Joseph L Dieleman, Angela Y Chang, Vegard Skirbekk, Sarah K Abe, Norito Kawakami, Erika Ota, Scott D Glenn, Chimedsuren Ochir, Hiroaki Miyata, Manami Inoue, Kenji Shibuya, Isaac Yeboah Addo, Mohammed Ahmed Akkaif, Syed Mahfuz Al Hasan, Waad Ali, Mohammad Al-Wardat, Hany Aly, Anayochukwu Edward Anyasodor, Jalal Arabloo, Ahmed Y. Azzam, Kavita Batra, Sonu Bhaskar, Samuel Adolf Bosoka, Ester Cerin, Vijay Kumar Chattu, Dong-Woo Choi, Bryan Chong, Samuel Demissie Darcho, Nicole Davis Weaver, Kuldeep Dhama, Robert Kokou Dowou, Temitope Cyrus Ekundayo, Ibrahim Farahat El Bayoumy, Pietro Ferrara, Nuno Ferreira, Takeshi Fukumoto, Xiang Gao, Samer Hamidi, Simon I Hay, Yuta Hiraike, Mehdi Hosseinzadeh, Nayu Ikeda, Arit Inok, Md. Rabiul Islam, Masao Iwagami, Ammar Abdulrahman Jairoun, Mihajlo Jakovljevic, Inn Kynn Khaing, Mohammad Jobair Khan, Atulya Aman Khosla, Tea Lallukka, Thao Thi Thu Le, Munjae Lee, Seung Won Lee, Wei-Chen Lee, Raimundas Lunevicius, Medha Mathur, Hadush Negash Meles, Mohammadreza Mobayen, Jama Mohamed, Abdollah Mohammadian-Hafshejani, Yanjinlkham Munkhsaikhan, Christopher J L Murray, Ganesh R Naik, Samidi Nirasha Kumari Navaratna, Phuong The Nguyen, Dieta Nurrika, Bogdan Oancea, Michael Safo Oduro, Takayoshi Ohkubo, Osaretin Christabel Okonji, Sok King Ong, Mahesh Padukudru P A, Jagadish Rao Padubidri, Romil R Parikh, Sungchul Park, Mahmoud Mohammed Ramadan, Shakthi Kumaran Ramasamy, Sheena Ramazanu, Elrashdy M. Moustafa Mohamed Redwan, Taeho Gregory Rhee, Cameron John Sabet, Vijaya Paul Samuel, Jennifer Saulam, Mohammad Ali Shamshirgaran, Premalatha K Shetty, Mika Shigematsu, Aminu Shittu, Emmanuel Edwar Siddig, Zhong Sun, Chandan Kumar Swain, Ruri Syailendrawati, Sree Sudha T Y, Jabeen Taiba, Masayuki Teramoto, Ngoc Ha Tran, Nguyen Tran Minh Duc, Dominique Vervoort, Muhammad Waqas, Kazumasa Yamagishi, Yuichiro Yano, Yuichi Yasufuku, Dong Keon Yon, Naohiro Yonemoto, Iman Zare, Zhiqiang Zhang, Hanqing Zhao, Claire Chenwen Zhong, and Mohsen Naghavi*

*Senior author

Affiliations

Global Research Institute (Prof S Nomura PhD), Keio University, Tokyo, Japan; Department of Global Health Policy (Prof S Nomura PhD, S K Rauniyar PhD), Department of Digital Mental Health (Prof N Kawakami PhD), Graduate School of Medicine (Y Hiraike PhD), University of Tokyo, Tokyo, Japan; Center for Infectious Disease Education and Research (Prof M Murakami PhD), Department of Biostatistics and Data Science (Y Yasufuku MSc), Osaka University, Suita, Japan; Department of Social Epidemiology (Prof N Kondo PhD), Kyoto University, Kyoto, Japan; Division of Epidemiology (T Tabuchi MD), Tohoku University, Sendai, Japan; Graduate School of Public Health (H Sakamoto PhD), St. Luke's International University, Tokyo, Japan; Department of Medicine (Y Tokuda MD), University of Tsukuba, Urasoe, Japan; Institute for Health Metrics and Evaluation (N Patel MSc, J L Dieleman PhD, S D Glenn MSc, N Davis Weaver MPH, Prof S I Hay FMedSci, Prof C J L Murray DPhil, Prof M Naghavi PhD, R Syailendrawati MA), Department of Health Metrics Sciences, School of Medicine (N Patel MSc, J L Dieleman PhD, Prof S I Hay FMedSci, Prof C J L Murray DPhil, Prof M Naghavi PhD), University of Washington, Seattle, WA, USA; Department of Research (J Navarro de Pablo PhD), Asian Development Bank, Tokyo, Japan; Danish Centre for Health Economics (A Y Chang DSc), University of Southern Denmark, Odense, Denmark; Danish Institute for Advanced

Study (A Y Chang DSc), University of Southern Denmark, Copenhagen, Denmark; Centre for Fertility and Health (Prof V Skirbekk PhD), Norwegian Institute of Public Health, Oslo, Norway; Healthcare Unit (S K Abe PhD), Economic Research Institute for ASEAN and East Asia, Jakarta, Indonesia; Institute for Cancer Control (S K Abe PhD), National Cancer Center, Tokyo, Japan; Department of Global Health Nursing (Prof E Ota PhD), St. Luke's International University, Chuo-ku, Japan; Department of Public Health and Health Policy (I Khaing MPH), Hiroshima University, Hiroshima, Japan; Advisory Board (Prof C Ochir PhD), Ministry of Health, Ulaanbaatar, Mongolia; Administration Department (B Lkhagvaa PhD), Mongolian National University of Medical Sciences, Ulaanbaatar, Mongolia; Department of International Cyber Education (Prof C Ochir PhD), Health Sciences University of Mongolia, Ulaanbaatar, Mongolia; School of Medicine (Prof H Miyata PhD), Keio University, Tokyo, Japan; Center for Public Health Sciences (M Inoue MD), Institute for Cancer Control (P T Nguyen DrPH), National Cancer Center, Chuo-ku, Japan; Medical Excellence JAPAN, Tokyo, Japan (Prof K Shibuya MD); School of Medicine (I Y Addo PhD), University of Sydney, Sydney, NSW, Australia; Centre for Social Research in Health (I Y Addo PhD), University of New South Wales, Sydney, NSW, Australia; Department of Cardiology (M Akkaif PhD), Fudan University, Shanghai, China; Division of Public Health Sciences, Department of Surgery (S Al Hasan PhD), Washington University in St. Louis, St. Louis, MO, USA; Department of Geography (W Ali PhD), Sultan Qaboos University, Muscat, Oman; Department of Rehabilitation Sciences (M Al-Wardat PhD), Jordan University of Science and Technology, Irbid, Jordan; Department of Pediatrics (Prof H Aly MD), Cleveland Clinic, Cleveland, OH, USA; Rural Health Research Institute (A E Anyasodor PhD), Charles Sturt University, Orange, NSW, Australia; Health Management and Economics Research Center (J Arabloo PhD), Iran University of Medical Sciences, Tehran, Iran; ASIDE Healthcare, Lewes, DE, USA (A Azzam MD); Faculty of Medicine (A Azzam MD), October 6 University, 6th of October City, Egypt; Department of Medical Education (K Batra PhD), University of Nevada Las Vegas, Las Vegas, NV, USA; Global Health Neurology Lab (S Bhaskar MD), NSW Brain Clot Bank, Sydney, NSW, Australia; Division of Cerebrovascular Medicine and Neurology (S Bhaskar MD), National Cerebral and Cardiovascular Center, Suita, Japan; Disease Surveillance Department (S A Bosoka MPhil), Ghana Health Service, Ho, Ghana; Department of Epidemiology and Biostatistics (S A Bosoka MPhil, R K Dowou MPhil), University of Health and Allied Sciences, Ho, Ghana; Mary MacKillop Institute for Health Research (Prof E Cerin PhD), Australian Catholic University, Melbourne, VIC, Australia; School of Public Health (Prof E Cerin PhD), University of Hong Kong, Hong Kong, China; Temerty Faculty of Medicine (V Chattu MD), University of Toronto, Toronto, ON, Canada; Department of Community Medicine (V Chattu MD), Datta Meghe Institute of Medical Sciences, Sawangi, India; Cancer Big Data Center (D Choi PhD), National Cancer Center, Goyang, South Korea; Department of Medicine (B Chong MBBS), Saw Swee Hock School of Public Health (S Ramazanu PhD), National University of Singapore, Singapore, Singapore; Department of Public Health (S D Darcho MPH), Haramaya University, Harar, Ethiopia; Division of Pathology (K Dhama PhD), ICAR-Indian Veterinary Research Institute, Bareilly, India; Department of Microbiology (T C Ekundayo PhD), University of Medical Sciences, Ondo, Ondo, Nigeria; Department of Public Health and Community Medicine (Prof I F El Bayoumy DrPH), Tanta University, Tanta City, Egypt; School of Public Health (Prof I F El Bayoumy DrPH), Texila American University, Guyana, Guyana; Center for Public Health Research (P Ferrara PhD), University of Milan Bicocca, Monza, Italy; Laboratory of Public Health (P Ferrara PhD), IRCCS Istituto Auxologico Italiano, Milan, Italy; Department of Social Sciences (Prof N Ferreira PhD), University of Nicosia, Nicosia, Cyprus; Department of Dermatology (T Fukumoto PhD), Kobe

University, Kobe, Japan; Department of Biostatistics (Prof X Gao PhD), Key Lab of Environment and Health (Prof X Gao PhD), Xuzhou Medical University, Xuzhou, China; School of Health and Environmental Studies (Prof S Hamidi DrPH), Hamdan Bin Mohammed Smart University, Dubai, United Arab Emirates; School of Computer Science (Prof M Hosseinzadeh PhD), Duy Tan University, Da Nang, Viet Nam; Jadara Research Center (Prof M Hosseinzadeh PhD), Jadara University, Irbid, Jordan; Center for Nutritional Epidemiology and Policy Research (N Ikeda PhD), National Institutes of Biomedical Innovation, Health and Nutrition, Osaka, Japan; Faculty of Health and Life Sciences (A Inok PhD), University of Exeter, Exeter, UK; School of Pharmacy (M Islam PhD), BRAC University, Dhaka, Bangladesh; Department of Health Services Research (M Iwagami PhD), Department of Public Health Medicine (Prof K Yamagishi MD), University of Tsukuba, Tsukuba, Japan; Department of Non-Communicable Disease Epidemiology (M Iwagami PhD), London School of Hygiene & Tropical Medicine, London, UK; Department of Health and Safety (A A Jairoun PhD), Dubai Municipality, Dubai, United Arab Emirates; The World Academy of Sciences UNESCO, Trieste, Italy (Prof M Jakovljevic PhD); Shaanxi University of Technology, Hanzhong, China (Prof M Jakovljevic PhD); Department of Rehabilitation Sciences (M Khan MPH), Hong Kong Polytechnic University, Hong Kong, China; Department of Internal Medicine (A A Khosla MD), Corewell Health East William Beaumont University Hospital, Royal Oak, MI, USA; Department of Medical Oncology (A A Khosla MD), Miami Cancer Institute, Miami, FL, USA; Department of Public Health (Prof T Lallukka PhD), University of Helsinki, Helsinki, Finland; University of Medicine and Pharmacy at Ho Chi Minh City, Ho Chi Minh City, Viet Nam (T T Le MD); Department of Medical Science (M Lee PhD), Ajou University School of Medicine, Suwon, South Korea; Department of Precision Medicine (Prof S Lee MD), Sungkyunkwan University, Suwon, South Korea; Department of Family Medicine (W Lee PhD), University of Texas Medical Branch, Galveston, TX, USA; Department of Emergency General and Trauma Surgery (Prof R Lunevicius DSc), Liverpool University Hospitals NHS Foundation Trust, Liverpool, UK; Department of Surgery (Prof R Lunevicius DSc), University of Liverpool, Liverpool, UK; Department of Community Medicine (M Mathur MD), Geetanjali Medical College and Hospital, Udaipur, India; Department of Medical Laboratory Sciences (H N Meles MSc), Adigrat University, Adigrat, Ethiopia; Burn and Regenerative Medicine Research Center (Prof M Mobayen MD), Guilan University of Medical Sciences, Tasht, Iran; College of Applied and Natural Science (J Mohamed MSc), University of Hargeisa, Hargeisa, Somalia; Modeling in Health Research Center (A Mohammadian-Hafshejani PhD), Shahrekord University of Medical Sciences, Shahrekord, Iran; Department of Community and Global Health (Y Munkhsaikhan MD), The University of Tokyo, Tokyo, Japan; College of Medicine and Public Health (G R Naik PhD), Flinders University, Adelaide, SA, Australia; Department of Engineering (G R Naik PhD), Western Sydney University, Sydney, NSW, Australia; Department of Community Medicine (S N K Navaratna MD), University of Peradeniya, Kandy, Sri Lanka; Postgraduate Institute of Medicine (S N K Navaratna MD), University of Colombo, Colombo, Sri Lanka; Hitotsubashi Institute for Advanced Study (HIAS) (P T Nguyen DrPH), Hitotsubashi University, Tokyo, Japan; Department of Public Health (D Nurrika PhD), Banten School of Health Science, South Tangerang, Indonesia; Ministry of Research, Technology and Higher Education (D Nurrika PhD), Higher Education Service Institutions (LL-DIKTI) Region IV, Bandung, Indonesia; Department of Applied Economics and Quantitative Analysis (Prof B Oancea PhD), University of Bucharest, Bucharest, Romania; Bioinformatics Department (Prof B Oancea PhD), National Institute of Research and Development for Biological Sciences, Bucharest, Romania; PSSM Data Sciences, Pfizer Research & Development (M Oduro PhD), Pfizer Inc., Groton, CT, USA; Department of Hygiene

and Public Health (Prof T Ohkubo MD), Teikyo University School of Medicine, Tokyo, Japan; School of Pharmacy (O C Okonji MSc), University of the Western Cape, Cape Town, South Africa; Department of Public Health (S Ong FAMS), Ministry of Health, Bandar Seri Begawan, Brunei; Institute of Health Sciences (S Ong FAMS), Universiti Brunei Darussalam, Bandar Seri Begawan, Brunei; Department of Respiratory Medicine (Prof M P P A DNB), Jagadguru Sri Shivarathreeswara University, Mysore, India; Department of Forensic Medicine and Toxicology (Prof J Padubidri MD), Manipal College of Dental Sciences, Mangalore (Prof P K Shetty MDS), Manipal Academy of Higher Education, Mangalore, India; Division of Health Policy and Management (R R Parikh MD), University of Minnesota, Minneapolis, MN, USA; Department of Health Policy and Management (S Park PhD), Korea University, Seoul, South Korea; Department of Clinical Sciences (Prof M M Ramadan PhD), University of Sharjah, Sharjah, United Arab Emirates; Department of Cardiology (Prof M M Ramadan PhD), Mansoura University, Mansoura, Egypt; Department of Radiology (S Ramasamy MD), Stanford University, Stanford, CA, USA; School of Nursing & Health Sciences (S Ramazanu PhD), Hong Kong Metropolitan University, Hong Kong, China; Department of Biological Sciences (Prof E M M Redwan PhD), King Abdulaziz University, Jeddah, Egypt; Department of Protein Research (Prof E M M Redwan PhD), Research and Academic Institution, Alexandria, Egypt; Department of Public Health Sciences (T Rhee PhD), University of Connecticut, Farmington, CT, USA; Department of Psychiatry (T Rhee PhD), Yale University, New Haven, CT, USA; Department of Medicine (C J Sabet MA), Georgetown University, Washington, DC, USA; Department of Anatomy (Prof V P Samuel PhD), Ras Al Khaimah Medical and Health Sciences University, Ras Al Khaimah, United Arab Emirates; Department of Medical Informatics (J Saulam MSc), Kagawa University, Miki-cho, Japan; Food Processing and Nutrition (J Saulam MSc), Karnataka State Akkamahadevi Women's University, Vijayapura, India; Department of Pathobiology (M Shamshirgaran PhD), Shahid Bahonar University of Kerman, Kerman, Iran; National Institute of Infectious Diseases, Tokyo, Japan (M Shigematsu PhD); Department of Veterinary Public Health and Preventive Medicine (A Shittu MSc), Usmanu Danfodiyo University, Sokoto, Sokoto, Nigeria; Unit of Basic Medical Sciences (E E Siddig MD), University of Khartoum, Khartoum, Sudan; Department of Medical Microbiology and Infectious Diseases (E E Siddig MD), Erasmus University, Rotterdam, Netherlands; Department of Biomedical Sciences (Z Sun PhD), Universiti Putra Malaysia, Selangor, Malaysia; Department of Analytical and Applied Economics (C Swain MPhil), Utkal University, Bhubaneswar, India; Department of Pharmacology (S T Y MD), All India Institute of Medical Sciences, Deoghar, India; Department of Environmental, Agricultural and Occupational Health (J Taiba PhD), University of Nebraska Medical Center, Omaha, NE, USA; Sri Ramachandra Medical College and Research Institute, Chennai, India (J Taiba PhD); Department of Preventive Medicine (M Teramoto MD), Northwestern University, Chicago, IL, USA; School of Biomedical Engineering (N Tran MD), University of Technology Sydney, Sydney, NSW, Australia; Molecular Neuroscience Research Center (N Tran Minh Duc MD), Shiga University of Medical Science, Shiga, Japan; Department of Health Policy and Management (D Vervoort MD), Johns Hopkins University, Baltimore, MD, USA; Key Laboratory of Computer-Aided Drug Design (M Waqas PhD), Guangdong Medical University, Dongguan, China; Department of Biotechnology and Genetic Engineering (M Waqas PhD), Hazara University Mansehra, Mansehra, Pakistan; Department of Public Health (Prof K Yamagishi MD, Prof N Yonemoto PhD), Faculty of Medicine (Y Yano MD), Juntendo University, Tokyo, Japan; Department of Pediatrics (Prof D Yon MD), Kyung Hee University, Seoul, South Korea; Department of Biostatistics (Prof N Yonemoto PhD), University of Toyama, Toyama, Japan; Research and Development Department (I Zare BSc), Sina Medical Biochemistry Technologies,

Shiraz, Iran; Tianjin Medical University General Hospital (Z Zhang MD), Tianjin Centers for Disease Control and Prevention, Tianjin, China; College of Traditional Chinese Medicine (H Zhao MD), Hebei University, Baoding, China; Jockey Club School of Public Health and Primary Care (C Zhong PhD), The Chinese University of Hong Kong, Hong Kong, China

Authors' Contributions

Providing data or critical feedback on data sources

Mohammad Al-Wardat, Jalal Arabloo, Ahmed Y. Azzam, Sonu Bhaskar, Vijay Kumar Chattu, Dong-Woo Choi, Bryan Chong, Samuel Demissie Darcho, Robert Kokou Dowou, Temitope Cyrus Ekundayo, Ibrahim Farahat El Bayoumy, Takeshi Fukumoto, Xiang Gao, Mehdi Hosseinzadeh, Nayu Ikeda, Md. Rabiul Islam, Mihajlo Jakovljevic, Norito Kawakami, Atulya Aman Khosla, Thao Thi Thu Le, Munjae Lee, Seung Won Lee, Wei-Chen Lee, Medha Mathur, Mohammadreza Mobayen, Abdollah Mohammadian-Hafshejani, Yanjinlkham Munkhsaikhan, Christopher J L Murray, Ganesh R Naik, Shuhei Nomura, Dieta Nurrika, Bogdan Oancea, Chimedsuren Ochir, Michael Safo Oduro, Sok King Ong, Mahesh Padukudru P A, Jagadish Rao Padubidri, Romil R Parikh, Sungchul Park, Mahmoud Mohammed Ramadan, Shakthi Kumaran Ramasamy, Sheena Ramazanu, Santosh Kumar Rauniyar, Taeho Gregory Rhee, Cameron John Sabet, Vijaya Paul Samuel, Premalatha K Shetty, Aminu Shittu, Chandan Kumar Swain, Sree Sudha T Y, Takahiro Tabuchi, Jabeen Taiba, Naohiro Yonemoto, Iman Zare, and Hanqing Zhao

Developing methods or computational machinery

Simon I Hay, Christopher J L Murray, and Shuhei Nomura

Providing critical feedback on methods or results

Isaac Yeboah Addo, Syed Mahfuz Al Hasan, Waad Ali, Mohammad Al-Wardat, Hany Aly, Anayochukwu Edward Anyasodor, Jalal Arabloo, Ahmed Y. Azzam, Kavita Batra, Sonu Bhaskar, Samuel Adolf Bosoka, Ester Cerin, Angela Y Chang, Vijay Kumar Chattu, Dong-Woo Choi, Bryan Chong, Samuel Demissie Darcho, Kuldeep Dhama, Robert Kokou Dowou, Temitope Cyrus Ekundayo, Ibrahim Farahat El Bayoumy, Takeshi Fukumoto, Xiang Gao, Samer Hamidi, Simon I Hay, Yuta Hiraike, Mehdi Hosseinzadeh, Arit Inok, Manami Inoue, Masao Iwagami, Ammar Abdulrahman Jairoun, Mihajlo Jakovljevic, Norito Kawakami, Inn Kynn Khaing, Mohammad Jobair Khan, Atulya Aman Khosla, Tea Lallukka, Thao Thi Thu Le, Munjae Lee, Seung Won Lee, Wei-Chen Lee, Medha Mathur, Hadush Negash Meles, HIROAKI MIYATA, Mohammadreza Mobayen, Jama Mohamed, Abdollah Mohammadian-Hafshejani, Yanjinlkham Munkhsaikhan, Michio Murakami, Christopher J L Murray, Ganesh R Naik, Jose Navarro de Pablo, Phuong The Nguyen, Shuhei Nomura, Dieta Nurrika, Bogdan Oancea, Michael Safo Oduro, Osaretin Christabel Okonji, Erika Ota, Mahesh Padukudru P A, Jagadish Rao Padubidri, Romil R Parikh, Sungchul Park, Nishali Patel, Mahmoud Mohammed Ramadan, Shakthi Kumaran Ramasamy, Santosh Kumar Rauniyar, Elrashdy M. Mohamed Moustafa Redwan, Taeho Gregory Rhee, Cameron John Sabet, Haruka Sakamoto, Vijaya Paul Samuel, Jennifer Saulam, Mohammad Ali Shamshirgaran, Kenji Shibuya, Aminu Shittu, Emmanuel Edwar Siddig, Zhong Sun, Chandan Kumar Swain, Sree Sudha T Y, Takahiro Tabuchi, Jabeen

Taiba, Masayuki Teramoto, Yasuharu Tokuda, Ngoc Ha Tran, Nguyen Tran Minh Duc, Dominique Vervoort, Muhammad Waqas, Kazumasa Yamagishi, Yuichi Yasufuku, Dong Keon Yon, Naohiro Yonemoto, Zhiqiang Zhang, and Claire Chenwen Zhong

Drafting the work or revising it critically for important intellectual content

Sarah K Abe, Isaac Yeboah Addo, Mohammed Ahmed Akkaif, Waad Ali, Mohammad Al-Wardat, Hany Aly, Anayochukwu Edward Anyasodor, Jalal Arabloo, Ahmed Y. Azzam, Sonu Bhaskar, Samuel Adolf Bosoka, Ester Cerin, Angela Y Chang, Vijay Kumar Chattu, Bryan Chong, Samuel Demissie Darcho, Nicole Davis Weaver, Joseph L Dieleman, Robert Kokou Dowou, Ibrahim Farahat El Bayoumy, Pietro Ferrara, Nuno Ferreira, Takeshi Fukumoto, Scott D Glenn, Simon I Hay, Yuta Hiraike, Mehdi Hosseinzadeh, Arit Inok, Manami Inoue, Md. Rabiul Islam, Mihajlo Jakovljevic, Norito Kawakami, Mohammad Jobair Khan, Atulya Aman Khosla, Naoki Kondo, Tea Lallukka, Thao Thi Thu Le, Wei-Chen Lee, Raimundas Lunevicius, Medha Mathur, Hadush Negash Meles, HIROAKI MIYATA, Abdollah Mohammadian-Hafshejani, Yanjinlkham Munkhsaikhan, Michio Murakami, Christopher J L Murray, Mohsen Naghavi, Samidi Nirasha Kumari Navaratna, Jose Navarro de Pablo, Shuhei Nomura, Bogdan Oancea, Takayoshi Ohkubo, Osaretin Christabel Okonji, Erika Ota, Mahesh Padukudru P A, Jagadish Rao Padubidri, Romil R Parikh, Nishali Patel, Mahmoud Mohammed Ramadan, Shakthi Kumaran Ramasamy, Santosh Kumar Rauniyar, Elrashdy M. Mohamed Moustafa Redwan, Cameron John Sabet, Haruka Sakamoto, Premalatha K Shetty, Kenji Shibuya, Mika Shigematsu, Aminu Shittu, Emmanuel Edwar Siddig, Vegard Skirbekk, Chandan Kumar Swain, Sree Sudha T Y, Takahiro Tabuchi, Yasuharu Tokuda, Nguyen Tran Minh Duc, Dominique Vervoort, Kazumasa Yamagishi, Yuichiro Yano, Yuichi Yasufuku, Dong Keon Yon, Naohiro Yonemoto, Iman Zare, Zhiqiang Zhang, and Claire Chenwen Zhong

Managing the estimation or publications process

Simon I Hay, Christopher J L Murray, Shuhei Nomura, and Ruri Syailendrawati
